# Assembling Shape-Persistent High-Order Sierpiński Triangular Fractals

**DOI:** 10.1016/j.isci.2020.101064

**Published:** 2020-04-16

**Authors:** Zhilong Jiang, Die Liu, Mingzhao Chen, Jun Wang, He Zhao, Yiming Li, Zhe Zhang, Tingzheng Xie, Feng Wang, Xiaopeng Li, George R. Newkome, Pingshan Wang

**Affiliations:** 1 Institute of Environmental Research at Greater Bay Area; Key Laboratory for Water Quality and Conservation of the Pearl River Delta, Ministry of Education; Guangzhou Key Laboratory for Clean Energy and Materials, Guangzhou University, Guangzhou-510006, China; 2 Hunan Key Laboratory of Micro & Nano Materials Interface Science; College of Chemistry and Chemical Engineering, Central South University, Changsha, Hunan-410083, China; 3 Department of Chemistry, University of South Florida, Tampa, FL 33620, USA; 4 Center for Molecular Biology and Biotechnology, Florida Atlantic University, Jupiter, FL 33428, USA

**Keywords:** Supramolecular Technologies, Supramolecular Materials, Molecular Self-Assembly, Materials Characterization Techniques

## Abstract

Fractals are a series of intricate patterns with aesthetic, mathematic, and philosophic significance. The Sierpiński triangles have been known for more than one hundred years, but only recently discrete shape-persistent low-generation (mainly ST-1) fractal supramolecules have been realized. Herein, we report a retro-assembly pathway to the nanometer-scale, supra-macromolecular second-generation Sierpiński triangle and its third-generation saturated counterpart (Pascal's triangle). These gigantic triangular assemblies are unambiguously confirmed by NMR, DOSY, ESI-MS, TWIM-MS, TEM, and AFM analyses. Notably, the dense-packed counterions of these discrete triangular architectures could further form supramolecular hydro-gels in water. This work not only provides a fundamental chemical pathway to explore various giant supramolecular constructs and further overcome the synthetic limitation of complicated molecular fractals, but also presents a new type of supramolecular hydro-gels with potential in controlled release applications.

## Highlights

• Giant supramacromolecular Sierpiński and Pascal's triangles
• The design and use of small synthons to assemble high-order molecular fractals
• A retro-assembly modular construction strategy for complicated molecular fractals
• The dense-packed counterions of architectures could form supramolecular hydro-gels

## Graphical Abstract

**Figure:**
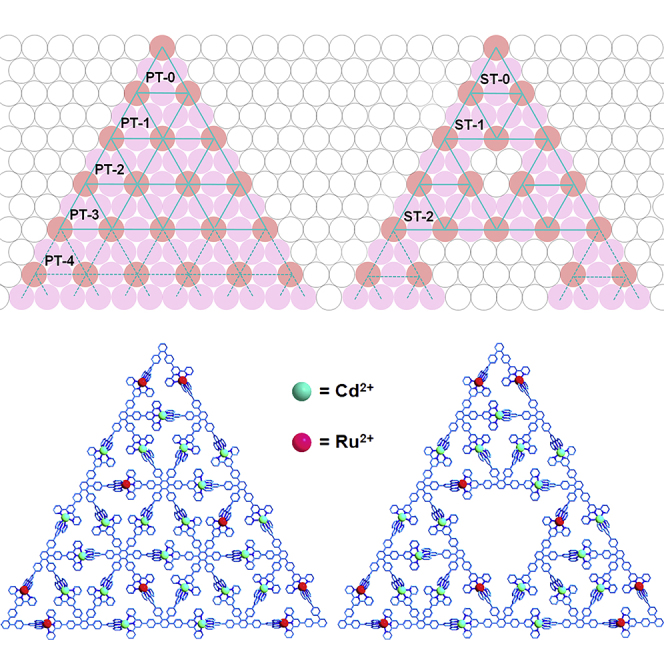

## Introduction

**Figure 1:**
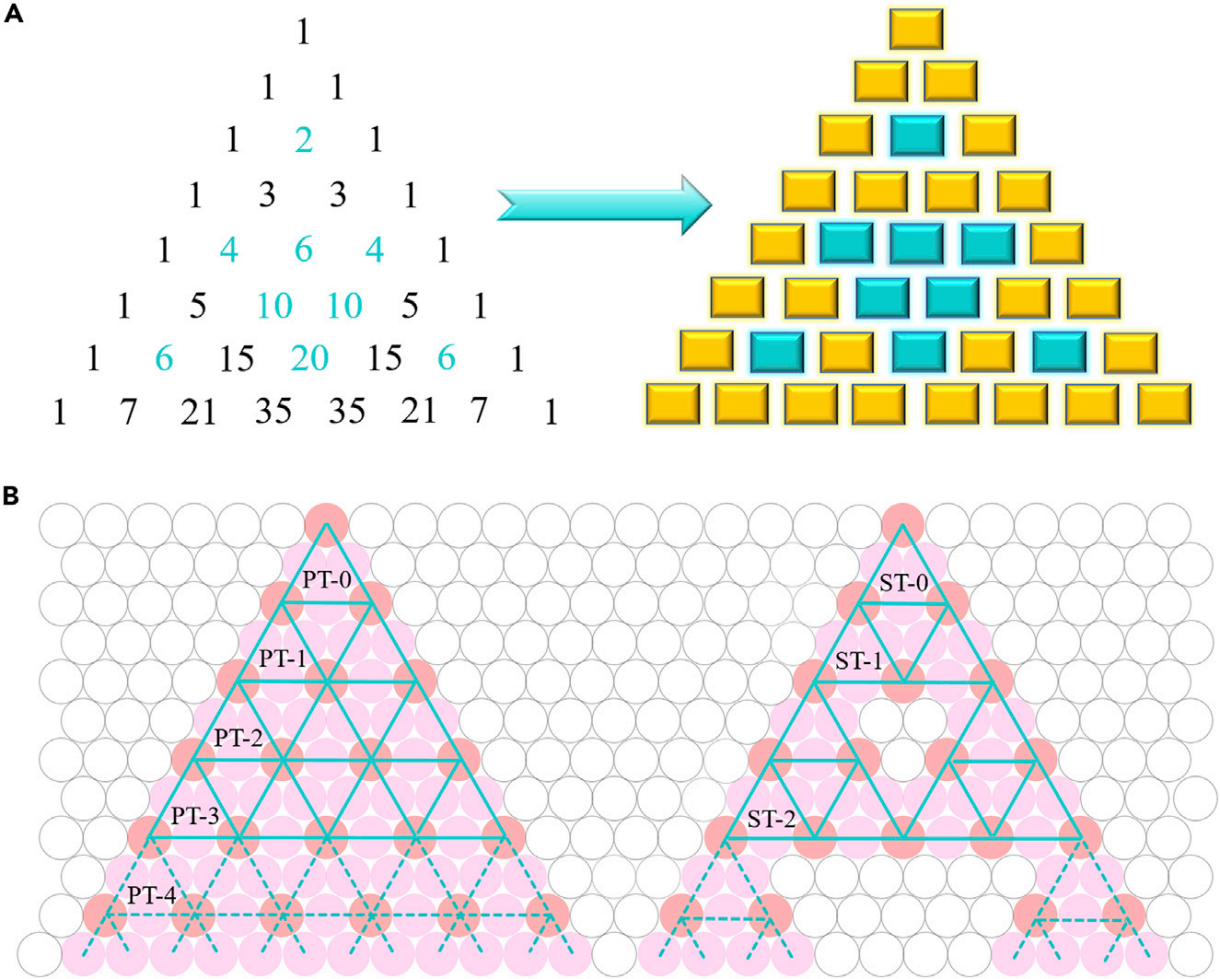
Mathematical Triangular Array of the Binomial Coefficients (A) The first few lines of binomial coefficients (yellow rectangles indicate the odd numbers and blue rectangles indicate the even numbers, the result is an approximation to the Sierpiński triangle). (B) The Pascal triangle (**PT**) and Sierpiński triangle (**ST**) pattern.

Self-similar fractals were described as “exactly the same at every scale or nearly the same at different scales” by Mandelbrot in 1975 (Mandelbrot, 1982, Peitgen et al., 1992). Examples of such fractal patterns are apparent in Nature, such as clouds, trees, and coast lines, but chemists need to find new one-step routes traditional and yet unknown fractal structures by simple one-step chemical methods (Lehn, 1985, Wang et al., 2015, Wang et al., 2018, Newkome et al., 2006; Newkome and Moorefield, 2015, Grayson and Fréchet, 2001, Sugiura et al., 1999, Rousseaux et al., 2015, Kempkes et al., 2018). As a basic example of self-similar fractal set, the Sierpiński triangle (**ST**; Figure 1) is a mathematically generated pattern that possesses the overall shape of an equilateral triangle and subdivided infinitely into smaller equilateral triangles. It was mathematically defined by the Polish mathematician Waclaw Sierpiński in 1916 via a series of interrelated equilateral triangles (Sierpinski, 1916). For Pascal's triangle (**PT**; Figure 1), it is a triangular array of the binomial coefficients, which is named after the French mathematician Blaise Pascal (Wolfram, 1984). If coloring the Pascal's triangle with 2n rows the even numbers blue and the odd numbers yellow, the result is an approximation to the Sierpiński triangle (Figure 1).

The earliest molecular-scale STs in experimental systems were achieved by self-assembly of DNA tiles (Brune et al., 1994). In recent years, efforts have been made to construct molecular **STs**, especially via different self-assembly strategy on surfaces (Shang et al., 2015, Li et al., 2017, Qiang et al., 2015, Rastgoo-Lahrood et al., 2016, Zhang et al., 2015, Nieckarz and Szabelski, 2016). For example, Wu's group and Wang's group have demonstrated a series of defect-free **STs** on noble metal surface at strictly low temperatures and under high-vacuum conditions from the 120° backbone of building blocks (Shang et al., 2015, Li et al., 2017). Wang and co-workers recently reported that covalent **STs** based on dynamic covalent chemistry could be prepared in solution on highly oriented pyrolytic graphite (HOPG) surface (Mo et al., 2019). In addition, driving forces including hydrogen bond and metal-ligand interaction were also used to prepare ordered STs on different surfaces (Qiang et al., 2015, Rastgoo-Lahrood et al., 2016, Cai et al., 2017).

It is worth noting that the on-surface construction of **STs** tends to form a mixture of different generations of **STs** rather than a discrete shape-persistent architecture. Alternatively, the self-assembly of supramolecular architectures through coordination of metal ions and predesigned ligands is an important part of supramolecular chemistry (Safont-Sempere et al., 2011, Swiegers and Malefetse, 2000, Yam et al., 2015, Holliday and Mirkin, 2001, De et al., 2010). Since Pt(II), Pd(II), and Fe(II) have been used as vertices to link different ligands leading to the construction of complicated 2D and 3D architectures, the use of precise organic-based vertices expands on this concept in which the ligand-metal-ligand component is used to appear as the sides of the construct (Byrne et al., 2017, Chakrabarty et al., 2011, Chichak et al., 2004, Cullen et al., 2016, Danon et al., 2017, Fujita et al., 2016a, Fujita et al., 2016b, Li et al., 2014a, Li et al., 2014b, Lu et al., 2017, Ronson et al., 2017). Among these coordination-driven constructs, fractal macromolecules are regarded as the most aesthetical design. However, it has been synthetically challenging especially those with multi asymmetric heterogeneous ligands, and thus limited examples have thus far been reported (Sarkar et al., 2014, Jiang et al., 2017a, Jiang et al., 2017b, Wang et al., 2014, Wu et al., 2017).

Recently, the low generation of **ST** and **PT** were synthesized in solution by Newkome group and our group (Sarkar et al., 2014, Jiang et al., 2017a, Jiang et al., 2017b, Schultz et al., 2012), but the construction of their next-generation architectures by employing similar methods carries significant challenges (Figures S1–S3). To the best of our knowledge, the single defect-free G2 **ST** and G3 **PT** have not been reported yet. Herein, we present the extremely challenging classical structures by molecular self-assembly based on three predesigned < tpy-Ru^2+^-tpy > monomers in solution for the first time (tpy = 2,2′:6′,2″-terpyridine), which consist of sixteen and twelve small triangles, respectively. The resultant giant architectures with a fractal feature can be assumed as a recursive mathematical form that possesses a self-similar structure, i.e., triangles of different sizes or levels.

**Figure 2:**
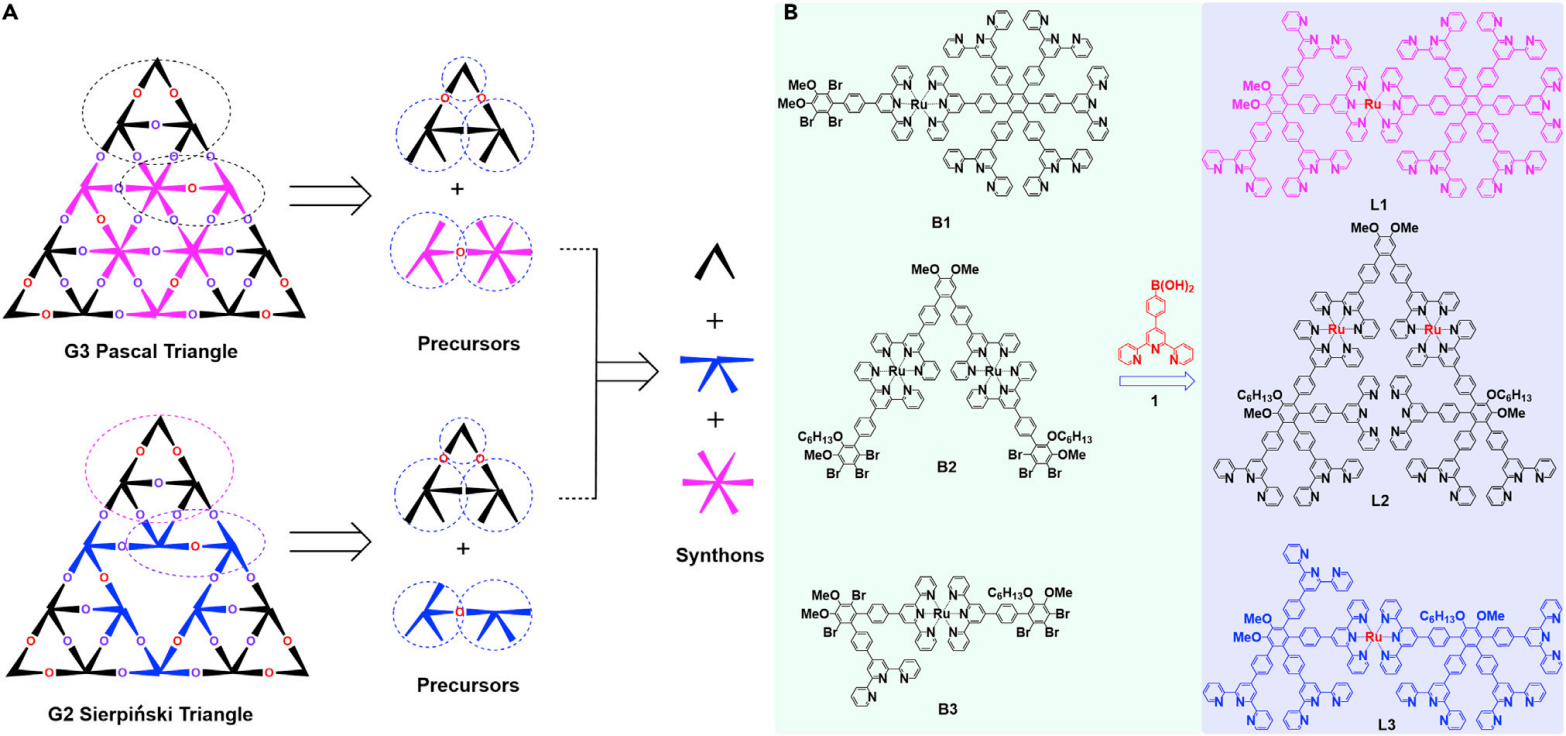
Retrosynthetic Analysis and Ligands Synthesis (A) Illustrating the disconnection of G3 **PT** and G2 **ST** to three precursors and three simplified synthons by the retrosynthetic analysis. (B) Synthesis of modular Metallo-organic Ligands through a multi-fold Pd^0^-catalyzed Suzuki coupling reaction on Ru-complexes. Reagents and conditions: CH_3_CN, Pd(PPh_3_)_4_, K_2_CO_3_, reflux.

Motivated by the retrosynthetic path devised by Corey Corey, 1991, an assembly strategy has been applied to analyze the geometrical patterns of target G2 **ST** and G3 **PT** (Figure 2A). Thus, the disconnections of metallo-terpyridinyl G2 **ST** and G3 **PT** fractals are the coordinated metal ions, instead of disconnecting the covalent bonds in a conventional retrosynthesis. Based on the triangular connections, the precursor for G2 **ST** and G3 **PT** could be distinguished as separate key metallo-organic ligands **L1**, **L2**, and **L3**, which utilize stable < tpy-Ru^2+^-tpy > as a linker. Thus, using three key expanded synthons, “V”-shaped, “K”-shaped, and “Star”-shaped ligands (Figure 2A), the desired multi-direct precursors could be specifically constructed by the Suzuki coupling reactions on complexes using the stable designer < tpy-Ru^2+^-tpy > derivatives. These pre-organized modular units may help the precursors and metal ions accurately assemble to generate the ultimate desirable architectures overcoming the entropy effect, as a result of thermodynamic control.

## Results and Discussion

### Ligands Synthesis

In terpyridyl chemistry, Ru(II) has been regarded as one of the most important metal ions to coordinate with terpyridine moieties to generate the inert <tpy-Ru^2+^-tpy> bonds. In our previous work, the synthetic route to <tpy-Ru^2+^-tpy> complexes containing uncoordinated terpyridine moieties utilized Suzuki coupling reactions (Jiang et al., 2017a, Jiang et al., 2017b, Chakraborty and Newkome, 2018). Based on this premise, the key metallo-organic ligands **L1**, **L2**, and **L3** were designed and successfully synthesized by multiple Suzuki-coupling of corresponding Br-substituent Ru^2+^-terpyridine complexes **B1**, **B2**, **B3** and 4'-(4-boronophenyl)-terpyridine **1** in a mixed solvent system with Pd(0) as catalyst, respectively (Figure 2B). The initial organic ligands were synthesized via known procedures (Jiang et al., 2017a, Jiang et al., 2017b, Chen et al., 2018a, Chen et al., 2018b). Those multibromo-substituted complexes were prepared by the coordination of the tpy-RuCl_3_ adducts with the “Star”-shaped hexa-tepyridine, “V”-shaped bisterpyridines under reducing conditions and simple column chromatography, respectively (Scheme S1). The detailed synthetic routes and procedures and characterizations are available in the Supporting Information (Figures S4–S40 and S51–S58). All the metallo-organic ligands and their precursors were fully characterized by nuclear magnetic resonance (NMR), including ^1^H, ^13^C, Homonuclear Chemical Shift Correlation Spectroscopy (COSY), and Nuclear Overhauser Effect Spectroscopy (NOESY).

The ^1^H NMR spectrum of **L2** features two peaks at 9.41 and 9.02 ppm with identified integral could be assigned to tpyH^3',5'^ of <tpy-Ru^2+^-tpy> moieties, singlets and doublets appeared at 8.77–8.54 ppm were assigned to the tpyH^3',5'^, tpyH^3,3″^, and tpyH^6,6″^ for the free terpyridine ligands (Figure S30). In addition, two sharp OCH_3_ singlets at 4.05 and 3.75 ppm and one triplet at 3.99 ppm for the OCH_2_ with a 3:3:2 integral ratio also confirmed the expected structure. The ^1^H NMR spectrum of **L1** and **L3** was complicated owing to several chemical environments of the terpyridine protons within the asymmetrical structure; there were ten and eight different terpyridine moieties in **L1** and **L3**, respectively. As for the ^1^H NMR spectrum of **L1** (Figure S21), it is clear that there were two groups of representative tpyH^3',5'^ singlets at above 9.0 ppm, which were assigned to tpyH^3',5'^ of the <tpy-Ru^2+^-tpy> moieties and four singlets below 9.0 ppm corresponding to free tpyH^3',5'^. Double singlets with an integration ratio 1:1 were found at 4.07, 4.05 ppm, which were assigned to the different methoxy groups. The ^1^H NMR spectrum of **L3** displayed two singlets at 8.87 and 9.07 ppm with identified integral could be assigned to tpyH^3',5'^ of <tpy-Ru^2+^-tpy> moieties, and the resonance absorption peaks ranging from 8.45 to 8.75 ppm correspond to protons of free terpyridine groups (Figure S38). Three sharp singlets around 3.75 ppm and one triplet at 3.97 ppm were in accordance with methoxyl and methylene groups of desired compound **L3**. All peaks assignments were confirmed based on 2D COSY and NOESY NMR spectra. To further establish the structure of these modules, electrospray ionization mass spectrometry (ESI-MS) was employed to show that all experimental values of ligands **L1–L3** are consistent with the theoretical values (Figures S54, S56, and S58).

### Self-Assembly of the Third-Generation Pascal's Triangle

**Figure 3:**
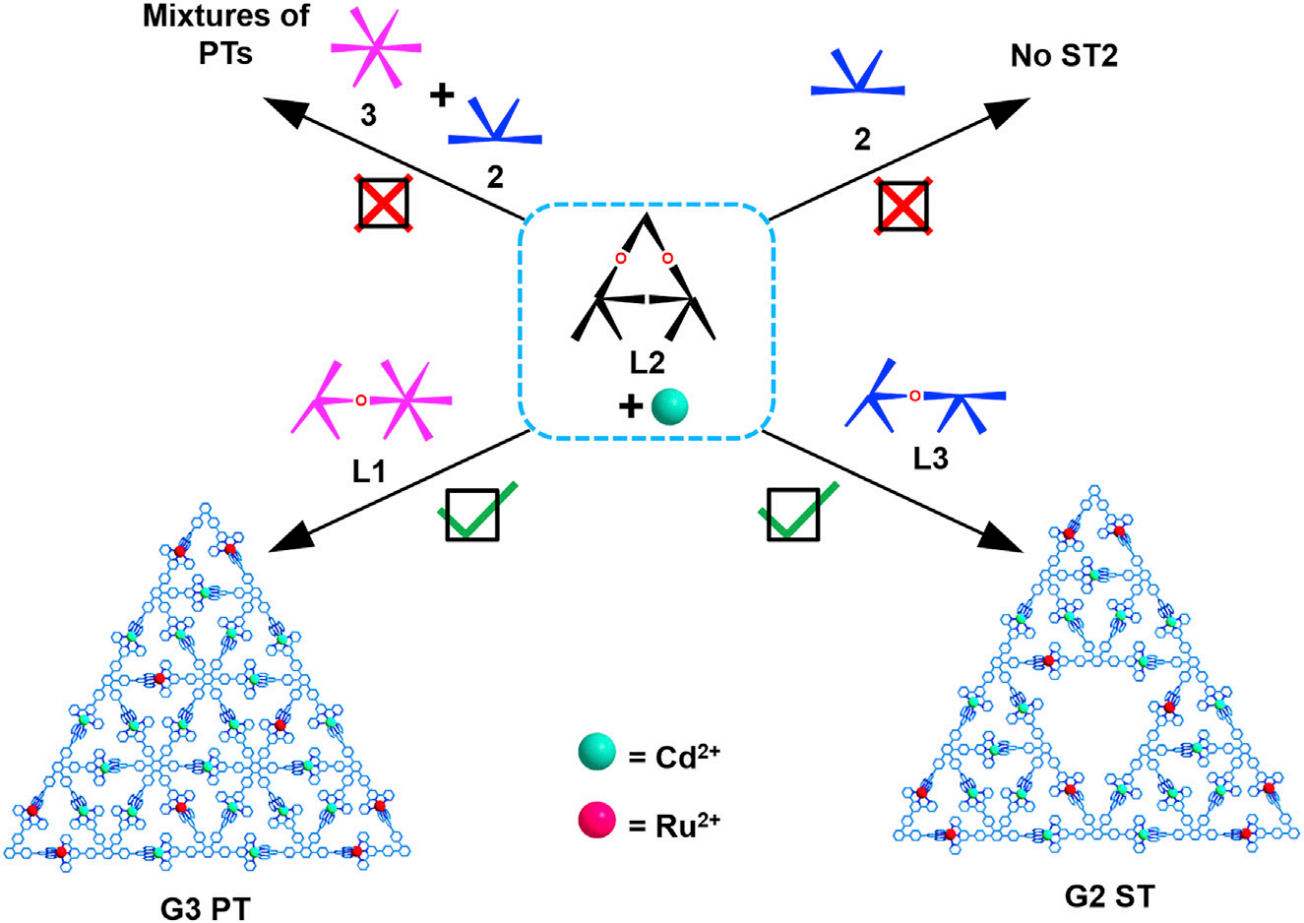
Schematic Illustrations of Preparing the Sierpiński-type Triangular Fractals from the Multicomponent Assembly Direct assembly of L2 with organic ligands failed to gain the expected architectures.

According to the geometric prediction, it was proposed that **L2**, K-shaped organic ligand **2,** and star shaped **3** could assemble with Cd^2+^ to form the desired G3 **PT** directly (Figure 3). The product was characterized by ESI-MS spectroscopy; unfortunately, only the G2 **PT** metallo-triangle architecture and a trace amount of G3 **PT** could be obtained (Figure S50). This was attributed to the fact that the self-assembly is an entropy-driven process, which needs to conquer the entropy reduction effect during the assembly. Similarly, the attempt to obtain G2 **ST** was, however, unsuccessful by directly mixing ligand **L2** and **2** with metal ions in a precise stoichiometric ratio of 1:2:7 via the one-pot procedure; the resultant product was unidentified by ESI-MS (Figures S49). We speculated that random assortment of free K-shaped ligands led to form the defective structures. Therefore, the Ru^2+^-tpy-based metallo-organic ligands **L1** and **L3** were redesigned through a retro-assembly analysis of targeted G2 **ST** and G3 **PT** (Video S1).

Video S1. Illustrating Carton Movie of Disconnection of G3 PT to Two Precursors and Three Synthons, Related to Figure 3

**Figure 4:**
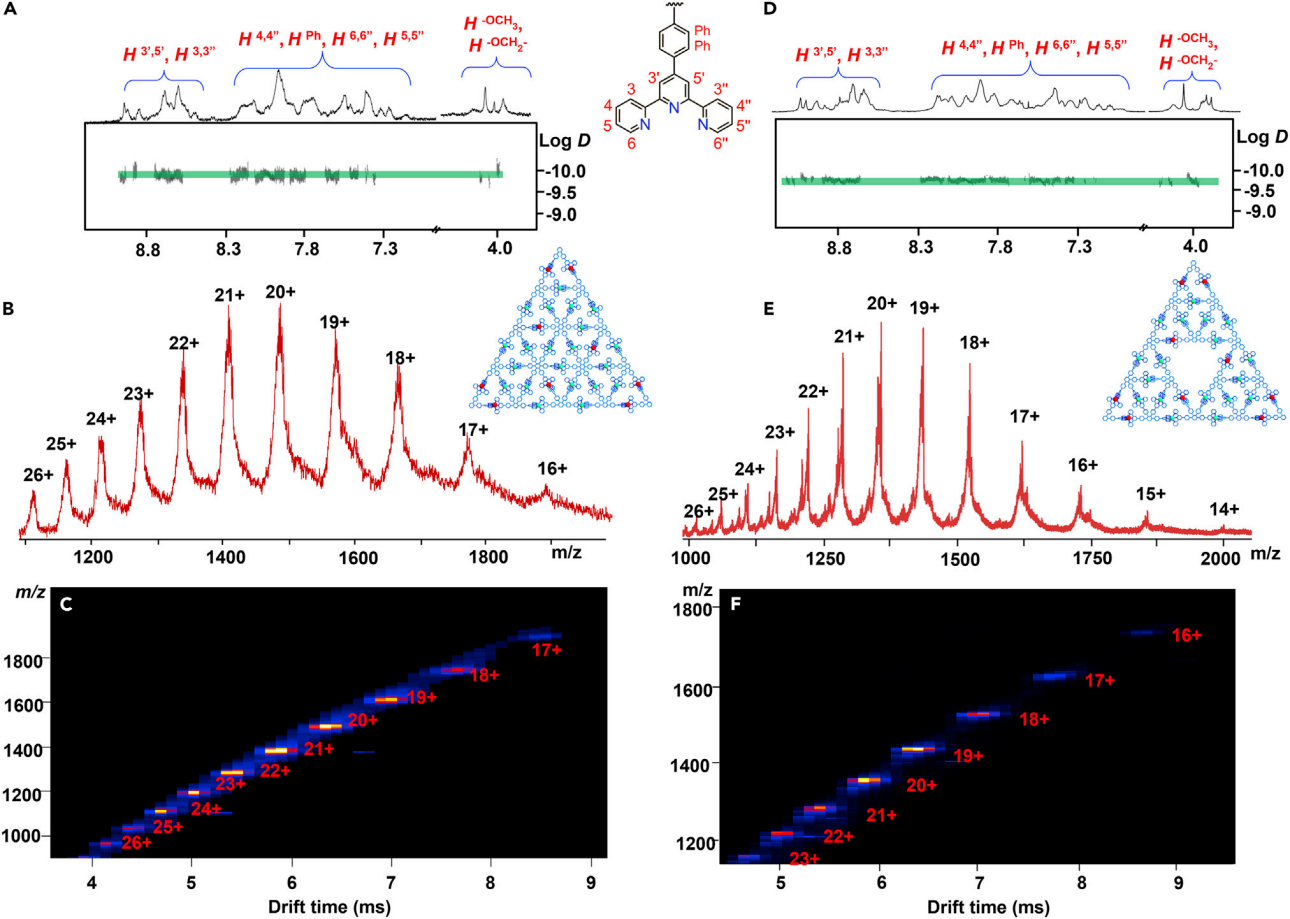
NMR Spectrum and Mass Spectrometry for Pascal's Triangle G3 PT and the Sierpiński Triangle G2 ST (A) 2D DOSY spectrum (500 MHz, CD_3_CN, 298 K) of G3 **PT**. (B) Electrospray ionization-mass spectrometry (ESI-MS) of G3 **PT**. (C) Traveling wave ion mobility mass spectrometry (TWIM-MS) of G3 **PT**. (D) 2D DOSY spectrum (500 MHz, CD_3_CN, 298 K) of G2 **ST**. (E) ESI-MS of G2 **ST**. (F) TWIM-MS of G2 **ST**.

Fortunately, after mixing modules **L1** and **L2** with Cd(NO_3_)_2_ in the precise ratio of 1:1:7 in MeOH/CHCl_3_ (1:1, v/v) at 75°C for 12 h, the multiple predesigned components were accurately assembled into the third-generation supramolecular Pascal's triangle G3 **PT**. Compared with the sharp ^1^H NMR signals of the ligands, the spectra of the supramolecular G3 **PT** displayed remarkable broaden peaks at aromatic area, owing to their much slower tumbling motion on the NMR timescale (Sun et al., 2010). But at aliphatic area, singlets of the methoxy groups and the triplet signal corresponding to the methylene group of G3 **PT** were observed at around 4.00 ppm; it showed complicated but sharp signals indicating the formation of a single assembly. All assignments were further confirmed by 2D COSY and NOESY spectra (Figures S41–S43). In particular, the diffusion-ordered spectroscopy (DOSY) spectrum of supramolecular G3 **PT** exhibited the only narrow band, confirming a single product was formed (Figure 4A). The observation of diffusion coefficient at 1.22×10^−10^ m^2^ s^−1^, according to the Stokes-Einstein equation (Einstein, 1956), the radius is determined to be 5.1 ± 0.5 nm, suggested the diameter of the supramolecular G3 **PT** is d = 10.2 ± 1.0 nm, which is consistent with the data of computer modeling (11.2 nm).

The ESI-MS spectrum of G3 **PT** (Figure 4B) exhibited a series of peaks at m/z 1,898.20, 1,780.04, 1,673.49, 1,577.27, 1,491.28, 1,413.47, 1,342.58, 1,277.56, 1,218.59, 1,164.32, and 1,113.54, with charge states from 16 + to 26 + via the loss of corresponding PF_6_¯ anions. Based on these signals, the molecular weight was calculated to be 32,725.88 Da, and this value was consistent with the theoretical ones (32,722.10 Da) that derived from the assembled composition of G3 **PT**. Unfortunately, the experimental isotopic pattern of each charge state was not obtained, possibly owing to the giant molecular weight beyond resolution of our ESI mass spectrometer, and such giant metallo-architecture may easily encapsulate numbers of solvent molecules owing to the large cavities. Moreover, traveling wave ion mobility mass spectrometry (TWIM-MS) was employed to verify the structural information; only one set of peaks on TWIM-MS excluded the formation of other isomers or conformers (Figure 4C). The valence change of Cd and Ru species was further confirmed by X-ray photoelectron spectroscopy (XPS) data. XPS peaks that appear at 281.1 and 285.3 eV are characteristic peaks of the Ru^2+^ and those that appear at 405.3 and 412.0 eV are characteristic peaks of the Cd^2+^ centers of G3 **PT** (Figure S59).

### Self-Assembly of the Second-Generation Sierpiński Triangle

The self-assembly of G2 **ST** was performed with modules **L2**, **L3**, and Cd^2+^ in a precise stoichiometric 1:1:7 ratio (Figure 3). The structure of G2 **ST** was confirmed by ESI-MS and NMR. The ^1^H NMR spectrum of G2 **ST** was also inevitably complicated owing to the presence of eighteen different terpyridine units and the slow tumbling motion of large complexes on the NMR timescale. Nevertheless, considerable structural information could be obtained by means of 2D COSY and NOESY NMR spectroscopy (Figures S44–S46). All tpyH^6,6″^ protons from the previously uncomplexed tpy units significantly shifted upfield owing to the electron shielding effects. The DOSY NMR of G2 **ST** that showed a distinct band with the diffusion coefficients at 1.13×10^−10^ m^2^ s^−1^ at 298 K indicated the formation of single product (Figure 4D). The diameter of the supramolecular G3 **ST** is also consistent with the result of computer modeling.

Mass spectrometry ESI-MS was primarily applied to characterize the G2 **ST** assembly. ESI-MS spectrum exhibited a series of peaks with charge states from 14 + to 26+, owing to the successive loss of PF_6_¯. These peaks were consistent very well with theoretical values of each charge state (Figure 4E). In addition, the exchange of the anions with PF_6_¯ also played a vital role in ESI-MS determination; the enlarged drawing exhibits two additional +19 ion peaks near [M-19PF_6_¯]^19+^, which was consistent with [M-20PF_6_¯+NO_3_¯]^19+^ and [M-21PF_6_¯+2NO_3_¯]^19+^. Such results were caused by an incomplete exchange of PF_6_¯ owing to the large number of exchangeable counter anions (Figure S47). The molecular weight for G2 **ST** was determined to be 30,061.54 Da, which was in accordance with the formula of [Cd_18_(C_244_H_180_N_30_O_6_Ru_2_)_3_(C_189_H_134_N_24_O_4_Ru)_3_]^54+^ (PF_6_¯)^54^. When a moderate ESI capillary voltage was applied, peaks with charge states from 12 + to 26+ were observed as a result of incorporating a different number of small solvent molecules (Figures S48 and S49). The TWIM-MS plot of G2 **ST** was obtained that can further serve as a good evidence for the absence of isomers as well as the high structural rigidity of the G2 **ST** (Figure 4F). Similarly, the presence of Cd^2+^ and Ru^2+^ ions was detected after deconvolution of the XPS data (Figure S60).

### Transmission Electron Microscopy and Atomic Force Microscopy Analysis

**Figure 5:**
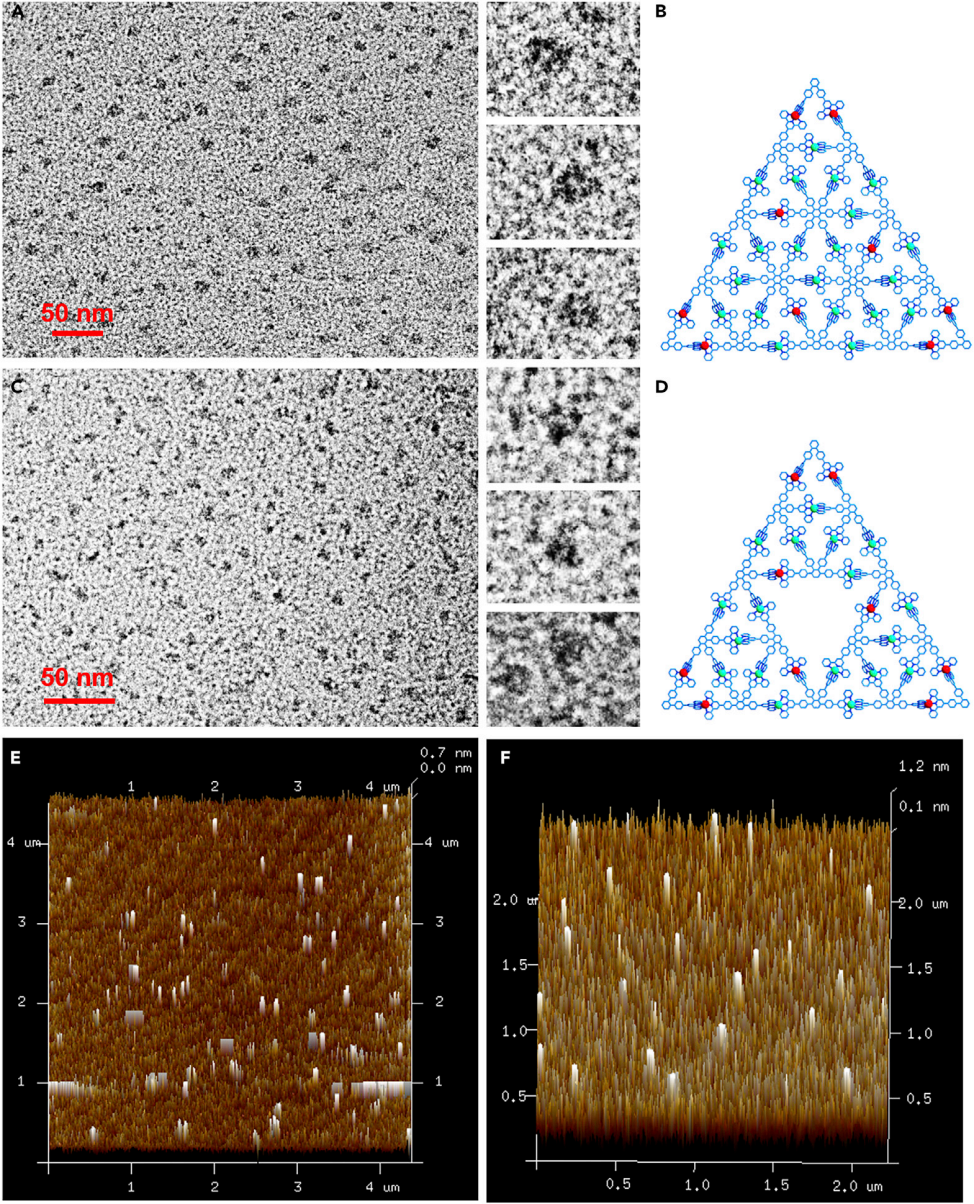
TEM and AFM Images TEM images of (A) Pascal's triangle G3 **PT** and (C) Sierpiński triangle G2 **ST**. Representative energy-minimized structures from molecular modeling of (B) G3 **PT** and (D) G2 **ST**. AFM images of (E) G3 **PT** and (F) G2 **ST**.

Any attempt to grow X-ray single crystals was unsuccessful. Alternatively, transmission electron microscopy (TEM) and atomic force microscopy (AFM) experiments were done to confirm the size and shape of the G3 **PT** and G2 **ST** (Figure 5). The deposition of G3 **PT** and G2 **ST** was conducted in a dilute (∼10^−6^ M) MeCN solution on carbon-coated grids (Cu, 400 mesh). From TEM pictures, both architectures were observed to be triangle-shadow patterns and the length of sides were about 11.6 ± 0.5 nm; the results were in agreement with the optimized molecular models (Figures S62 and S64). Specifically, a few patterns resembling the Star of David were also found in the TEM formed by the stacking of two G2 **ST** triangles (Figure S61), which was also shown for the G1 **ST**. (Sarkar et al., 2014) AFM images of G3 **PT** and G2 **ST** revealed a triangle-shadow morphology by dropping a dilute MeCN solution (∼10^−7^ M) on the mica surface, which were much larger than those of TEM and the theoretical simulation (Figures S63 and S65). Such results were derived from a tip broadening effect (Radmacher et al., 1994). However, the height of 0.8 ± 0.2 nm determined by AFM images matched well with energy-minimized (0.8 nm) structure obtained by molecular modeling (Bauer et al., 2011).

### The Reversible Gelation Behavior

**Figure 6:**
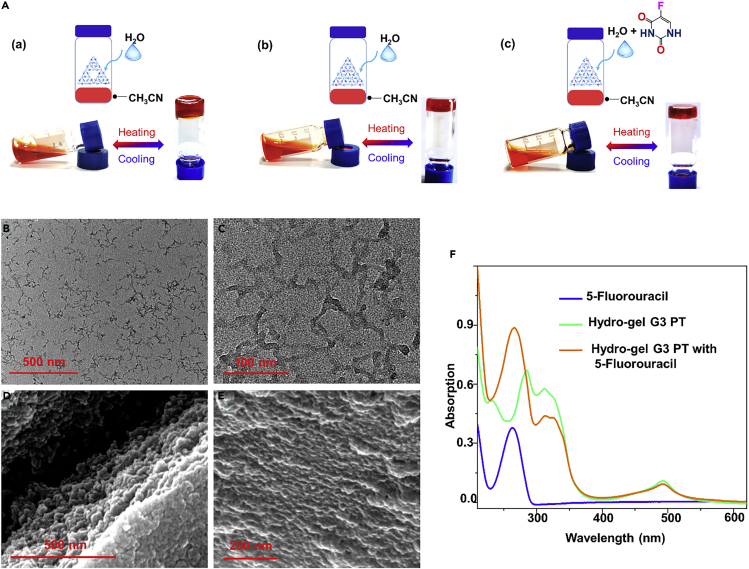
Reversible Gel-Sol Transitions of Supramolecular Hydro-Gels (A) Photographs of supramolecular hydro-gels formation in H_2_O and their thermal reversible gel-sol transitions (a: G2 **ST**, b: G3 **PT**, c: G3 **PT** with 5-fluorouracil). (B: scale bar 500 nm and C: scale bar 100 nm) TEM images of the solution of supramolecular hydro-gels G3 **PT** in H_2_O. (D: scale bar 500 nm and E: scale bar 200 nm) SEM images of the aggregates of the supramolecular hydro-gels G3 **PT** in H_2_O. (F) UV-vis absorption spectra of 5-fluorouracil, supramolecular hydro-gels G3 **PT,** and supramolecular hydro-gels G3 **PT** with 5-fluorouracil (10^−5^ M) in ethanol.

Notably, the self-assembled triangular architectures G3 **PT** and G2 **ST** were found to form supramolecular hydro-gels in water. By heating the solution of G3 **PT** and G2 **ST** in the mixture solvent of H_2_O/CH_3_CN (v/v, 10:1) and then cooling to room temperature gave red hydro-gels, respectively. It is interesting that the reversible gel-sol transitions could be realized through temperature stimuli; when the temperature increased, the formed supramolecular hydro-gels gradually turned into solutions, and the gels were able to reform when the temperature cooled down (Figures 6Aa and 6Ab). TEM and SEM (scanning electron microscopy) were employed to investigate the morphologies of the hydro-gel. As shown in Figures 6B–6E, the TEM images of G3 **PT**-based hydro-gel were observed to be nanofiber networks. The similar three-dimensional bulk networks were observed in the SEM images, which displayed the microstructures of the obtained hydro-gels. We expected that the supramolecular hydro-gels were generated owing to the dense-packed counterions of the coordinative and ionic interactions, hydrophobic interactions, solvent effect, π−π stacking, et al. (Cordier et al., 2008, Li et al., 2014a, Li et al., 2014b).

The supramolecular hydro-gel properties of the dense-packed counterions in these gigantic triangular assemblies could further promote the application of hydro-gel to adsorb and release water-soluble functional molecules. Take 5-fluorouracil as a representative; upon adding the aqueous solutions of the small molecule into acetonitrile solution of G3 **PT**, similar observation was obtained as described above, red transparent hydro-gel (Figures 6A–6C). The UV-vis spectra of molecule 5-fluorouracil, supramolecular hydro-gel G3 **PT**, and their mixture are shown in Figure 6F. The absorption of 5-fluorouracil in hydro-gel G3 **PT** shows a little difference from 5-fluorouracil with absorbance positions 267 and 263 nm, respectively, suggesting that the 5-fluorouracil was loaded in the hydro-gel. In fact, the latter formed supramolecular hydro-gel also possesses the reversible temperature-responsive gel-sol phase transitions; thus the small molecule was able to release in case of heating. The adsorption and release properties of such novel hydro-gels were expected (Chen et al., 2018a, Chen et al., 2018b).

### Conclusions

In conclusion, the supramolecular shape-persistent second-generation Sierpiński triangle and third-generation Pascal's triangle fractals were assembled from three metallo-precursors, which were redesigned through a retro-assembly analysis and synthesized via multi-fold Suzuki coupling reactions. The self-assembly of G2 **ST** and G3 **PT** with fractal features was employed by a modular strategy using <tpy-Ru^2+^-tpy> and <tpy-Cd^2+^-tpy> connectivity. Compared with one-pot self-assembly, modular strategy showed a highly spontaneous matching and avoided self-sorting as well as formation of oligomers; such predesigned ligands were more likely to form giant 2D architectures by self-assembly. Moreover, these gigantic triangular assemblies with dense-packed counterions and ionic interactions possess reversible gelation properties with potential applications in drug release. This work supports new pathways to design and construct giant discrete shape-persistent supramolecules based on terpyridine ligands or other ligands through retro-assembly analysis and modular strategy. These simple procedures also open the door to new precise functional nanomaterials by modification of simple directed monomers.

## Methods

All methods can be found in the accompanying Transparent Methods supplemental file.

## Supplemental Information

Document S1. Transparent Methods, Figures S1–S65, and Schemes S1 and S2
