# Supplementary material for: Assembling Shape-Persistent High-Order Sierpiński Triangular Fractals
Source: iScience. 2020 Apr 16;23(5):101064. doi: 10.1016/j.isci.2020.101064 (PMC7210427; doi:10.1016/j.isci.2020.101064)
Supplement: Document S1. Transparent Methods, Figures S1–S65, and Schemes S1 and S2 [file mmc1.pdf]

iScience, Volume *23*

Supplemental Information

Assembling Shape-Persistent High-Order Sierpiński Triangular Fractals

Zhilong Jiang, Die Liu, Mingzhao Chen, Jun Wang, He Zhao, Yiming Li, Zhe Zhang, Tingzheng Xie, Feng Wang, Xiaopeng Li, George R. Newkome, and Pingshan Wang

Supporting Information

Assembling shape-persistent high-order Sierpiński triangle fractals

Zhilong Jiang, Die Liu, Mingzhao Chen, Jun Wang, He Zhao, Yiming Li, Zhe Zhang, Tingzheng Xie, Feng Wang, Xiaopeng Li, George R. Newkome and Pingshan Wang

The Pascal triangle and Sierpiński Triangle pattern

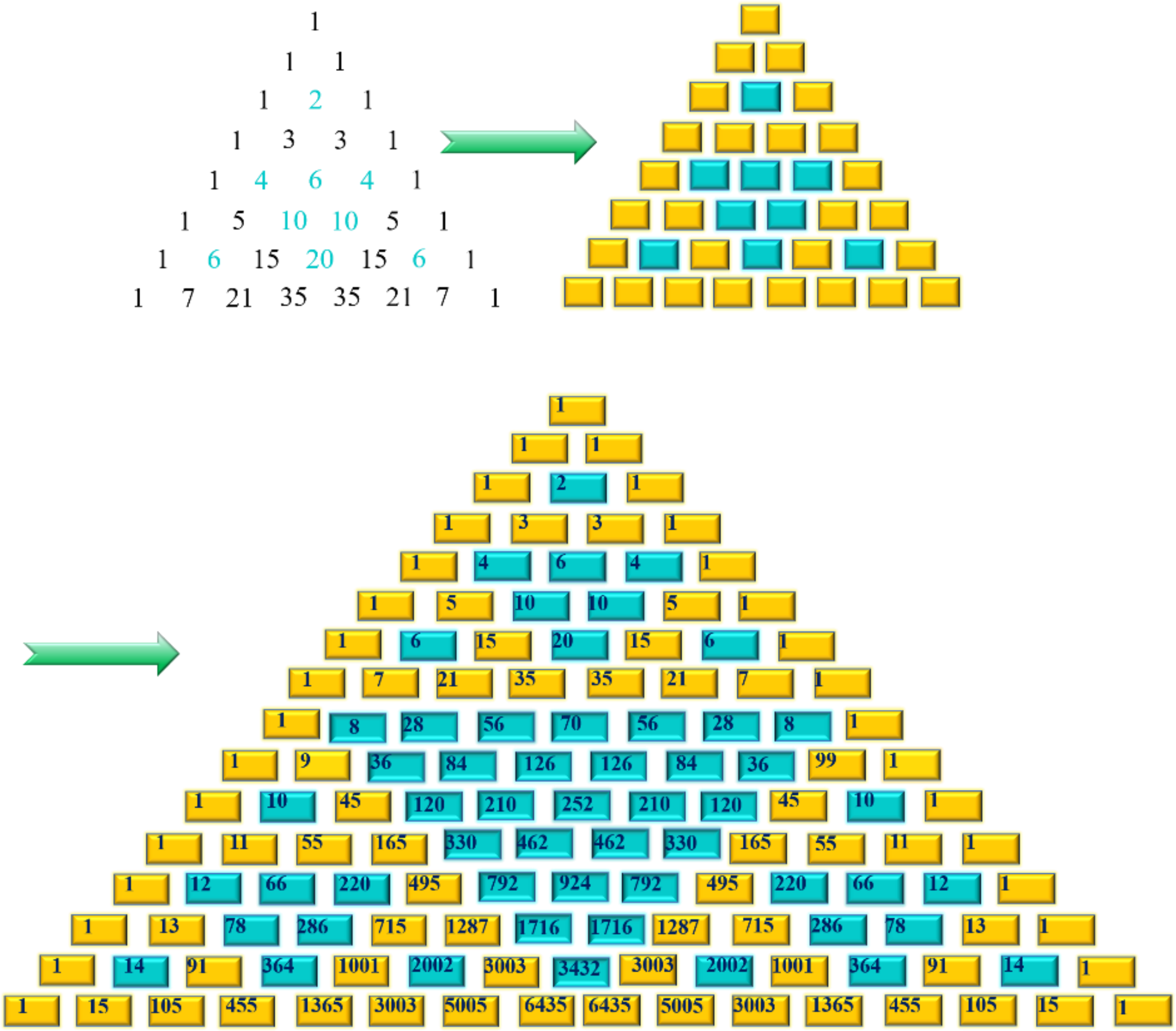

Figure S1. Mathematical triangular array of the binomial coefficients (yellow rectangles indicate the odd numbers and blue rectangles indicate the even numbers). Related to Figure 1.

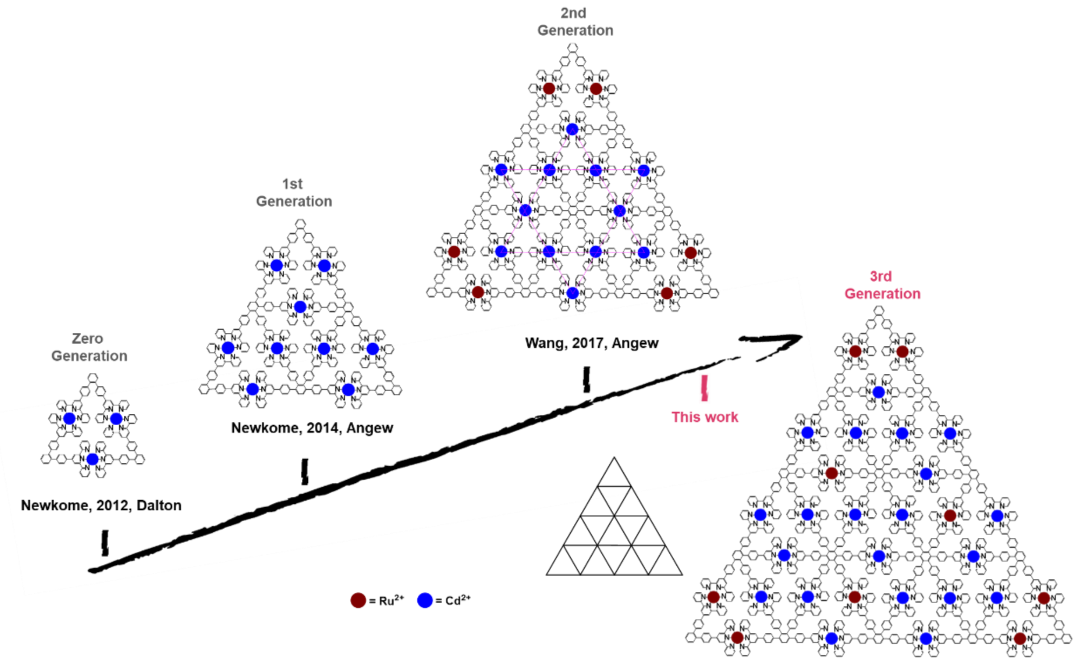

Figure S2. The whole family of the synthesized molecular PTs. Related to Figure 2.

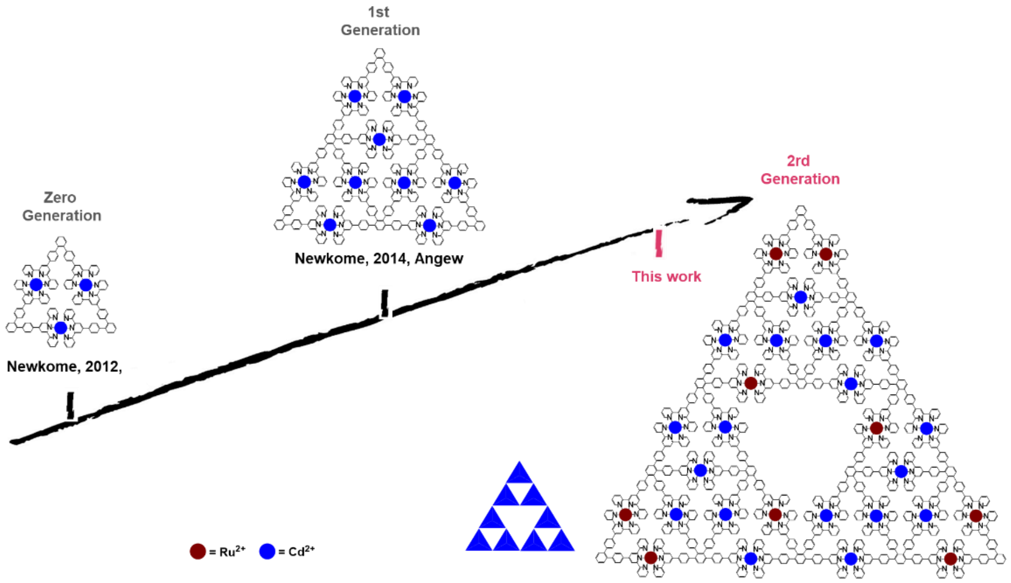

Figure S3. The whole family of the synthesized molecular STs. Related to Figure 2.

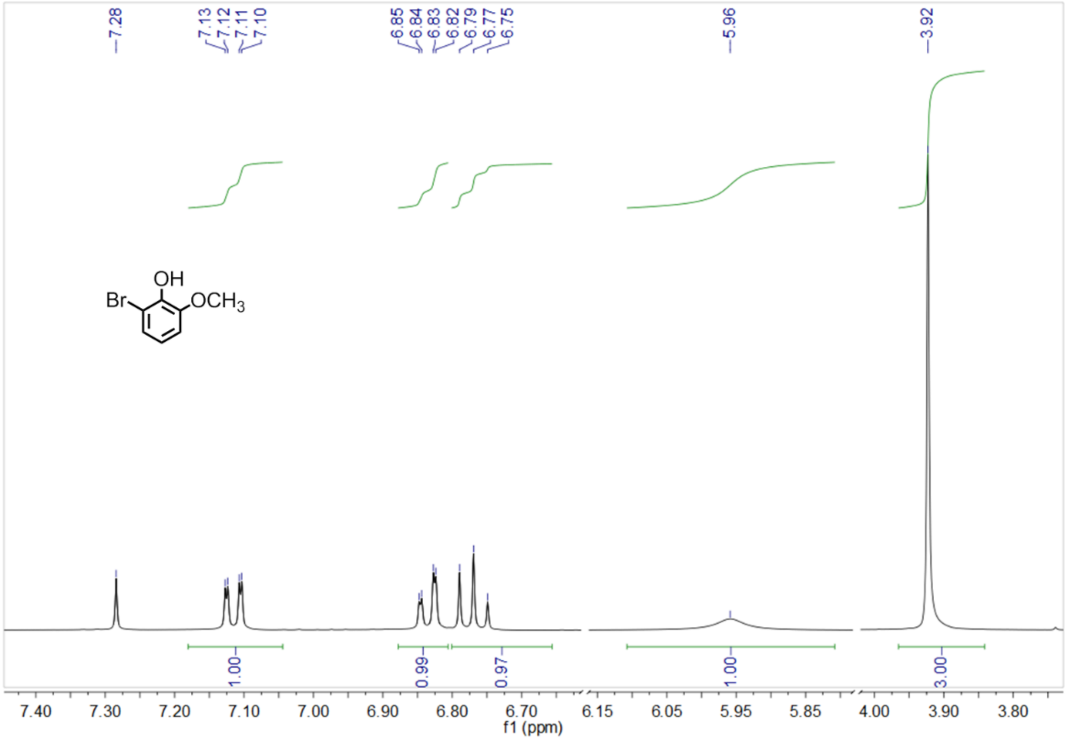

Figure S4. The ^{1}H NMR spectrum of **S2** (400 MHz) in $CDCl_3$. Related to Figure 2.

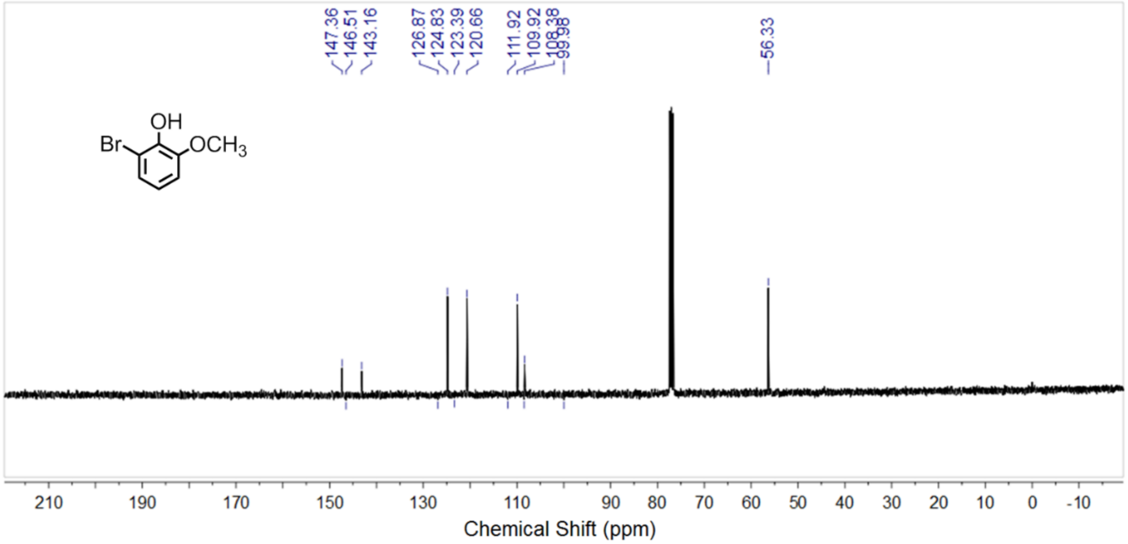

Figure S5. The ^{13}C NMR spectrum of **S2** (101 MHz) in $CDCl_3$. Related to Figure 2.

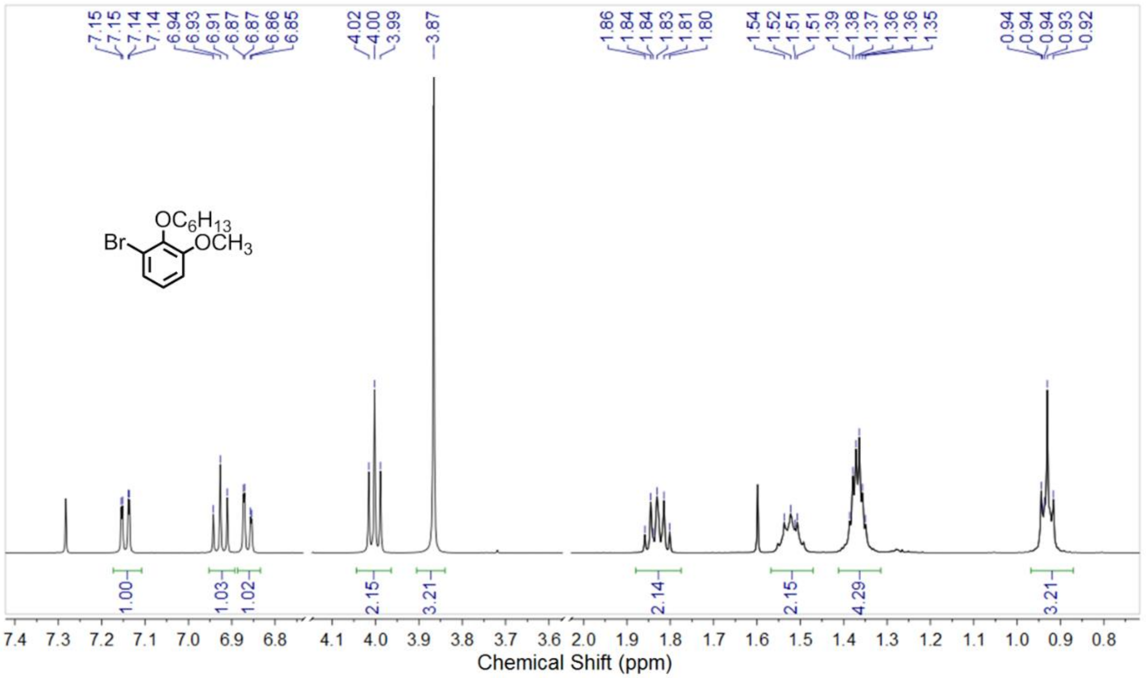

Figure S6. The ^{1}H NMR spectrum of **S3** (500 MHz) in $CDCl_3$. Related to Figure 2.

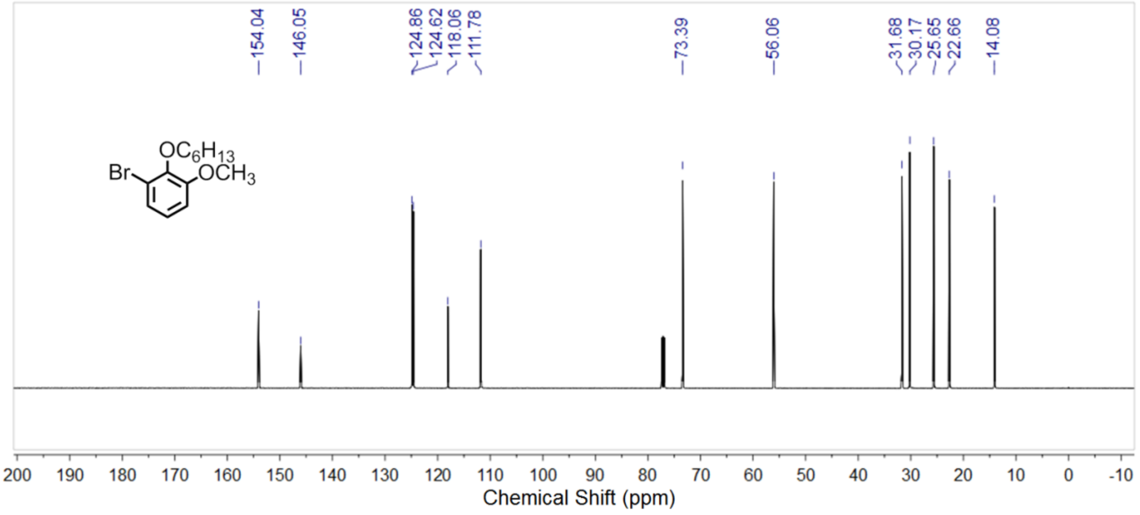

Figure S7. The ^{13}C NMR spectrum of **S3** (126 MHz) in $CDCl_3$. Related to Figure 2.

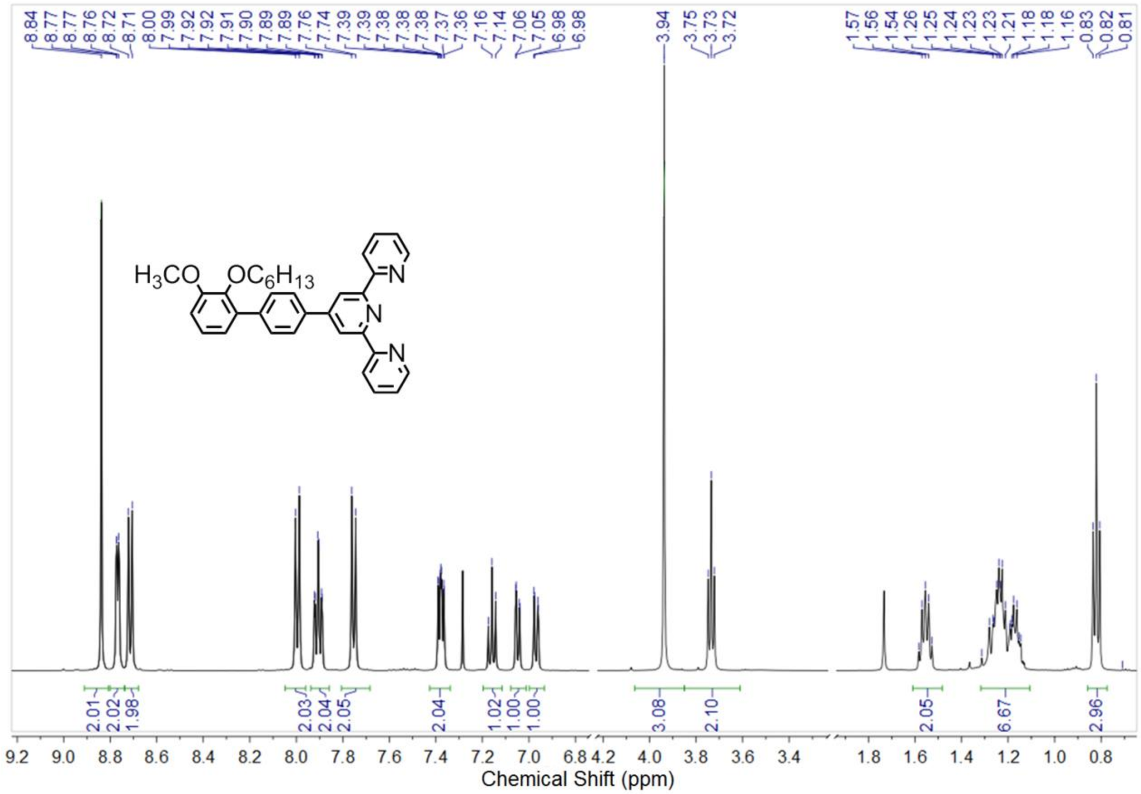

Figure S8. The ¹H NMR spectrum of **S4** (500 MHz) in $CDCl_3$. Related to Figure 2.

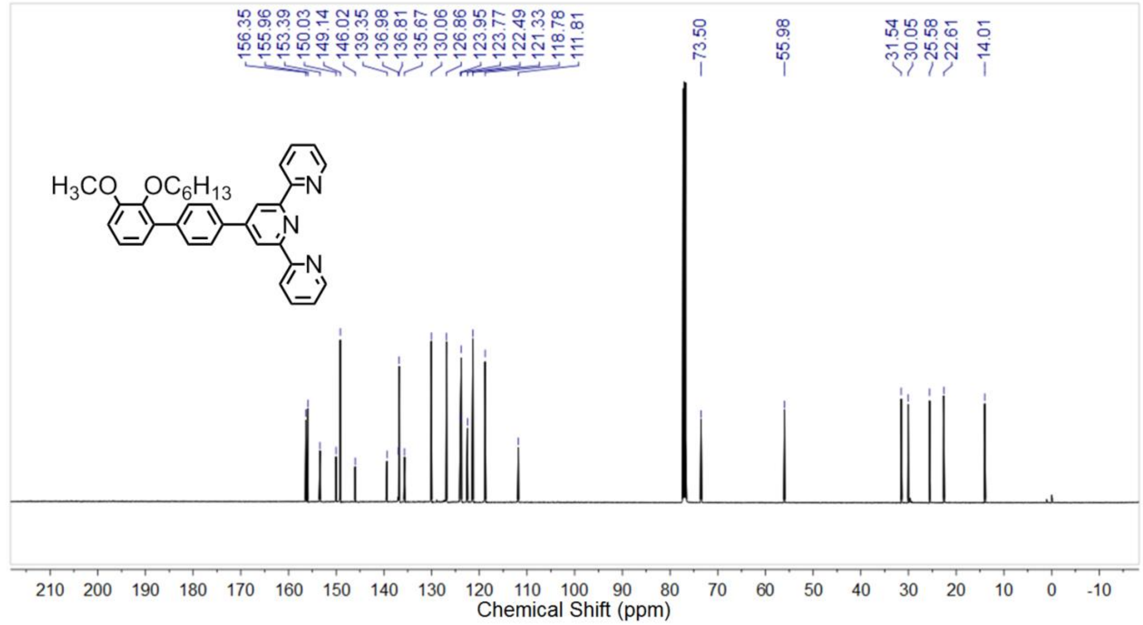

Figure S9. The ¹³C NMR spectrum of **S4** (126 MHz) in $CDCl_3$. Related to Figure 2.

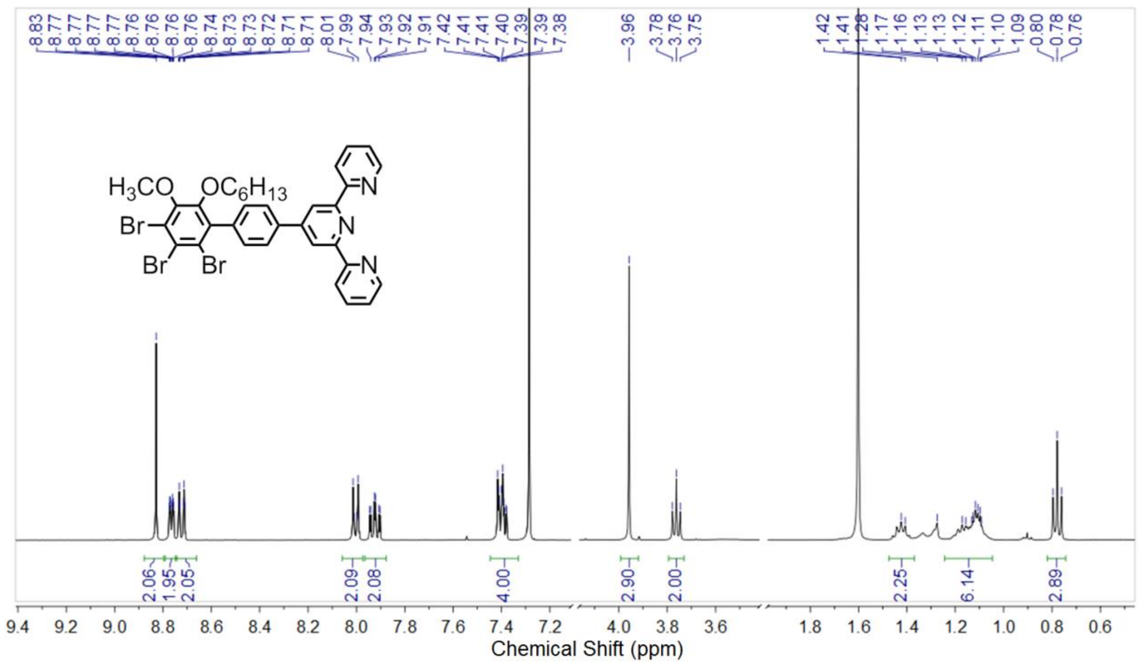

Figure S10. The ^{1}H NMR spectrum of **S5** (400 MHz) in $CDCl_3$. Related to Figure 2.

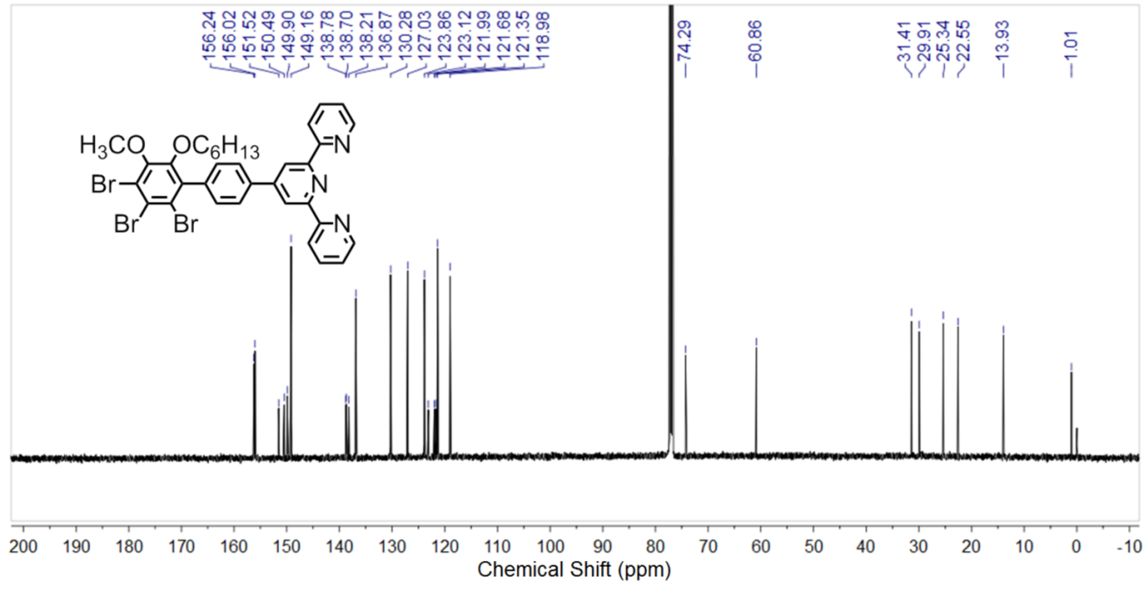

Figure S11. The ^{13}C NMR spectrum of **S5** (126 MHz) in $CDCl_3$. Related to Figure 2.

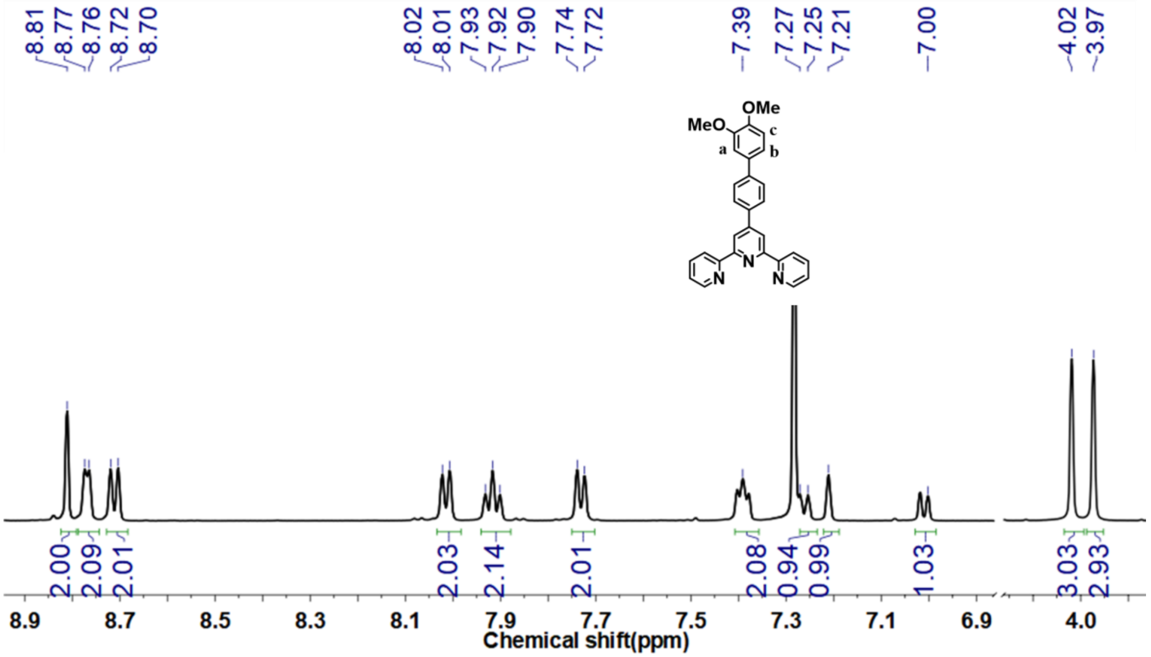

Figure S12. The ^{1}H NMR spectrum of **S7** (500 MHz) in $CDCl_3$. Related to Figure 2.

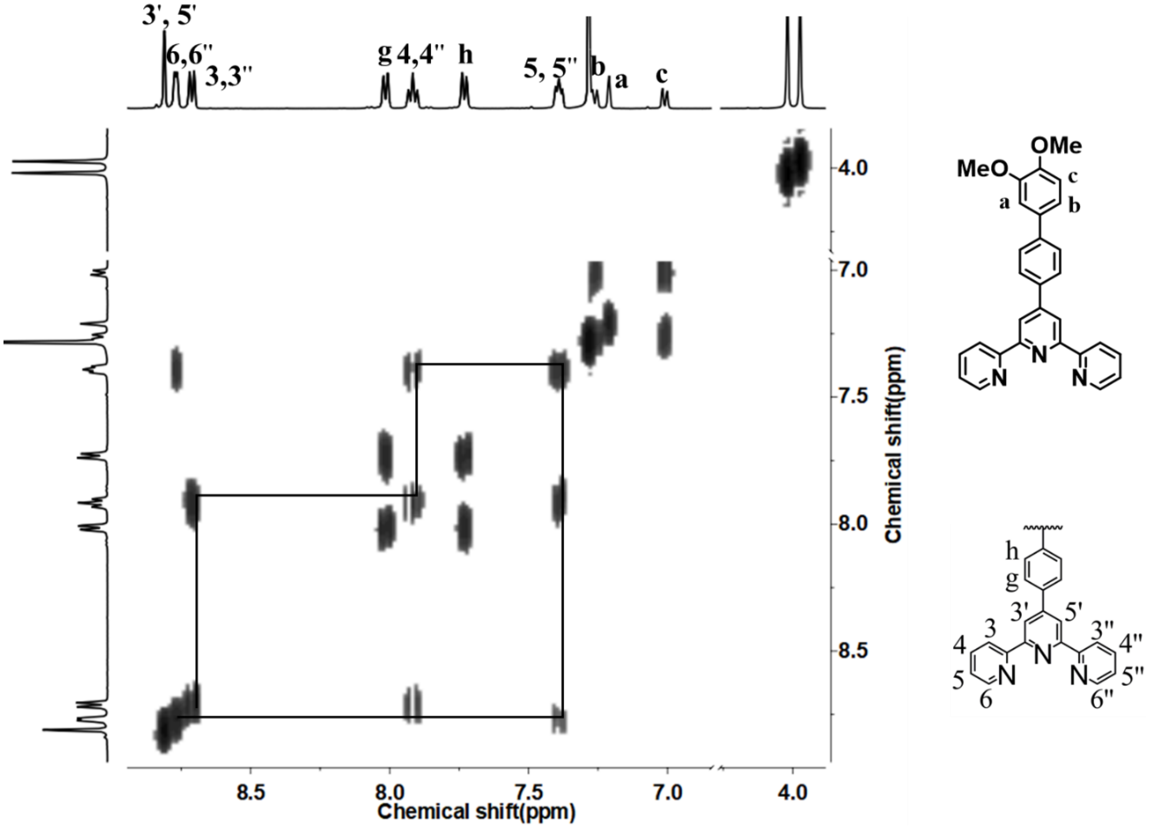

Figure S13. The 2D-COSY spectrum of **S7**(500 MHz) in $CDCl_3$. Related to Figure 2.

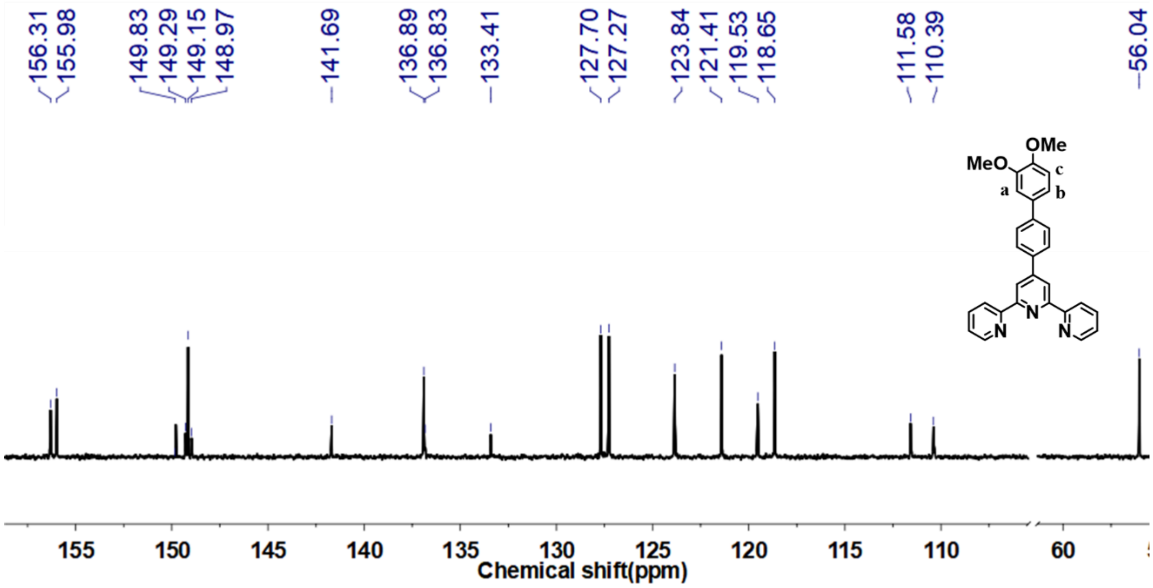

Figure S14. The ^{13}C NMR spectrum of **S7** (126 MHz) in $CDCl_3$. Related to Figure 2.

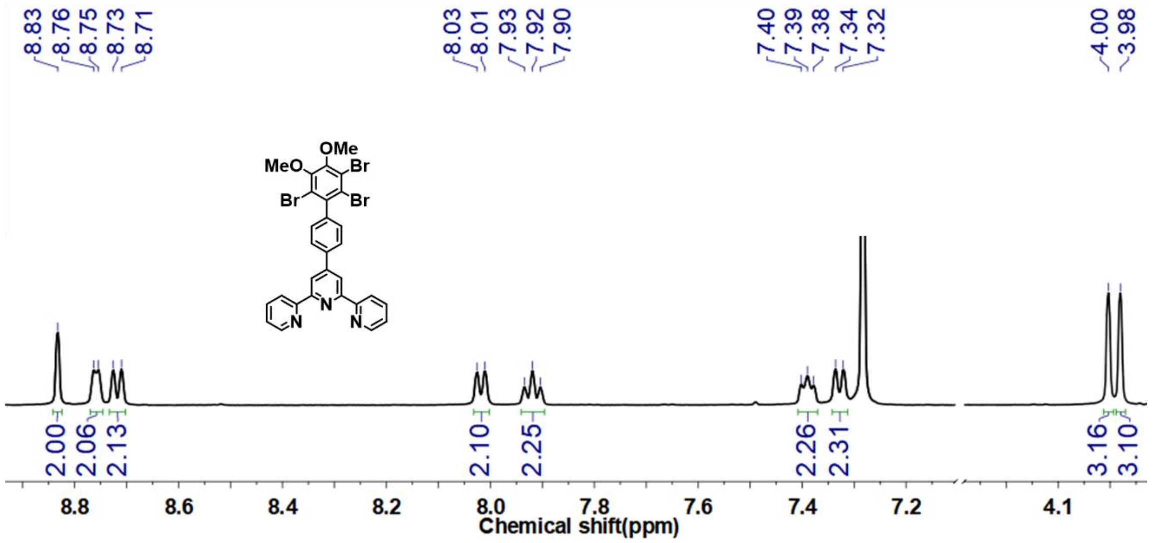

Figure S15. The ^{1}H NMR spectrum of **S8** (500 MHz) in $CDCl_3$. Related to Figure 2.

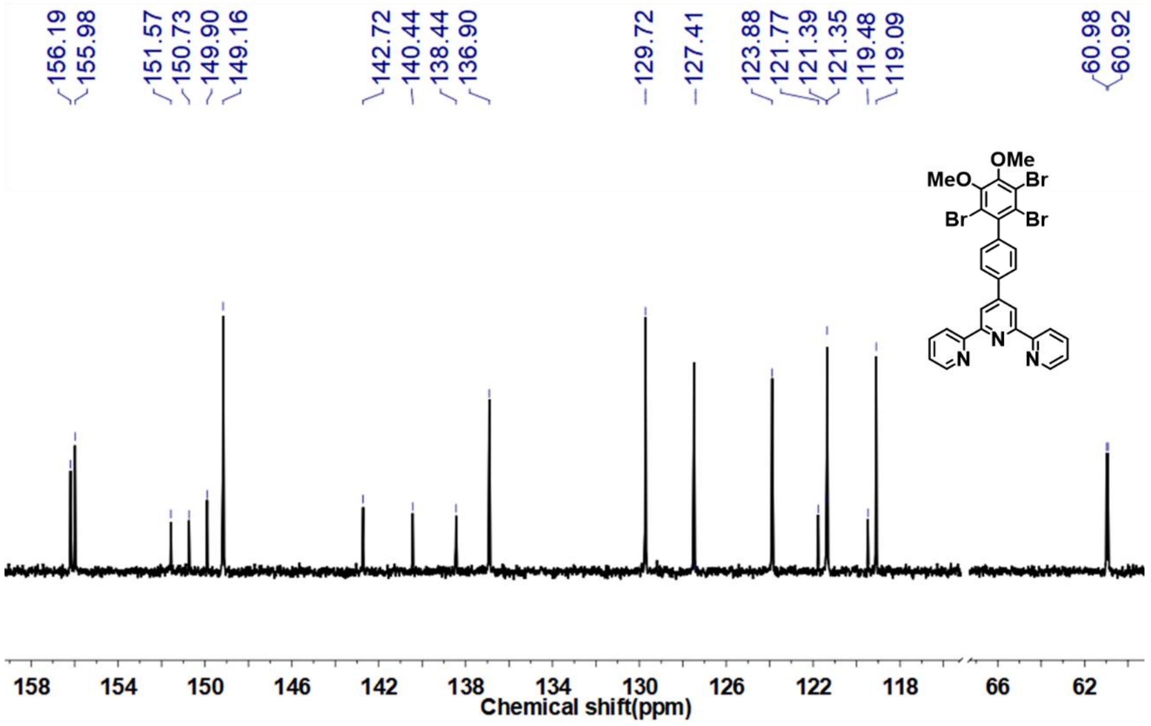

Figure S16. The ^{13}C NMR spectrum of **S8** (126 MHz) in $CDCl_3$. Related to Figure 2.

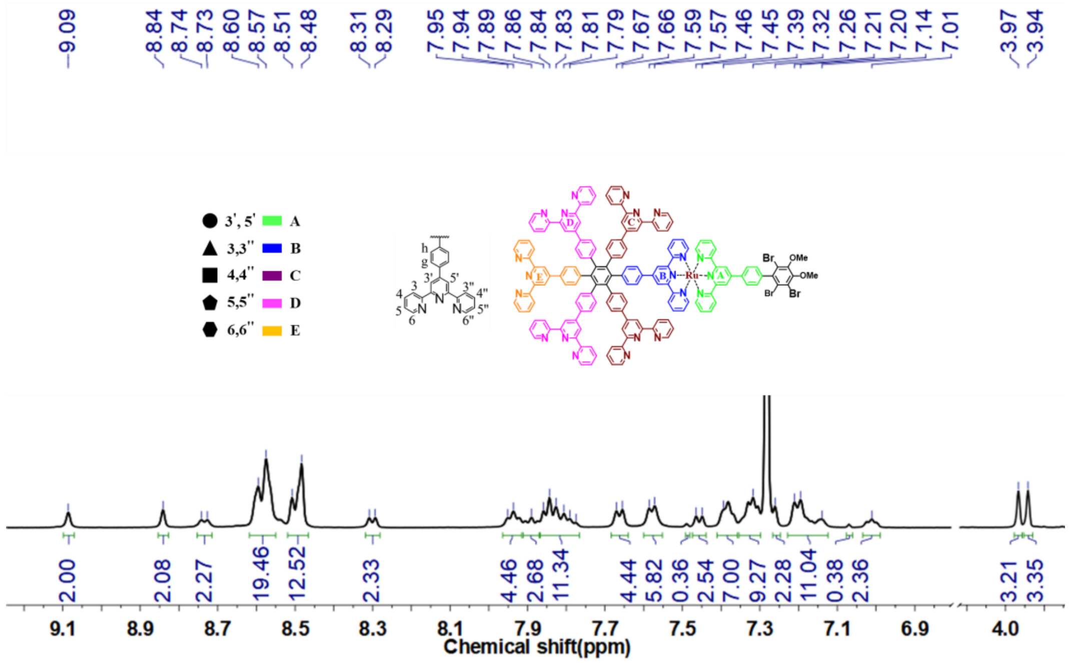

Figure S17. The ^{1}H NMR spectrum of **B1** (500 MHz) in $CDCl_3$. Related to Figure 2.

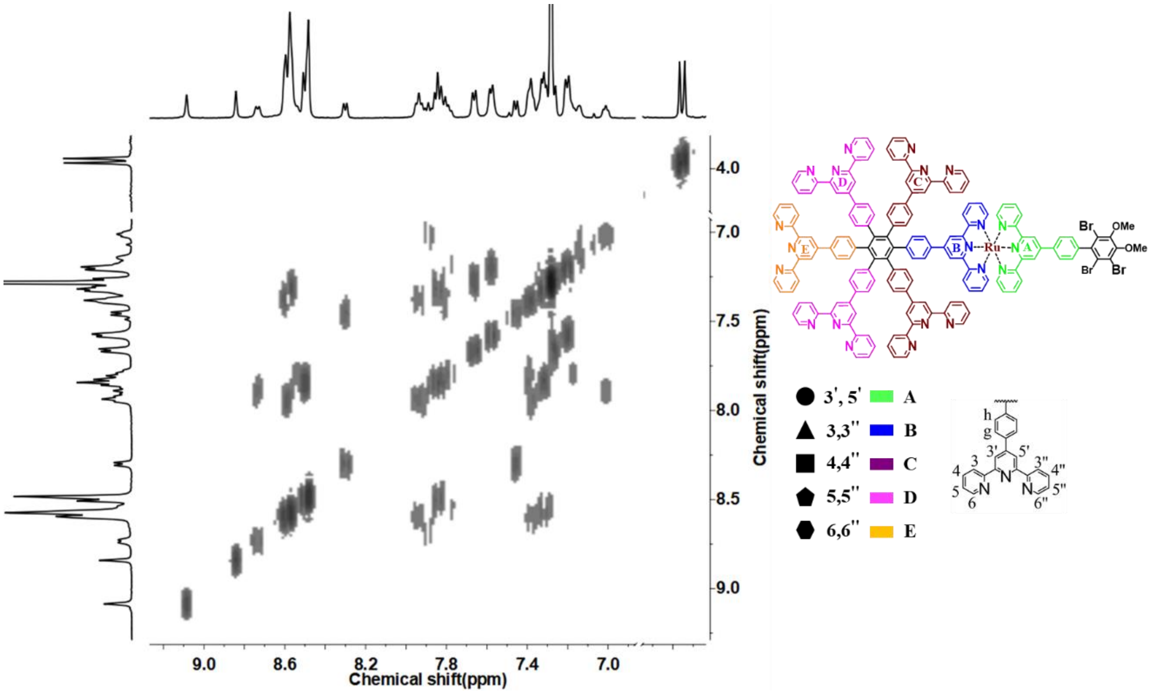

Figure S18. The 2D-COSY spectrum of **B1** (500 MHz) in $CDCl_3$. Related to Figure 2.

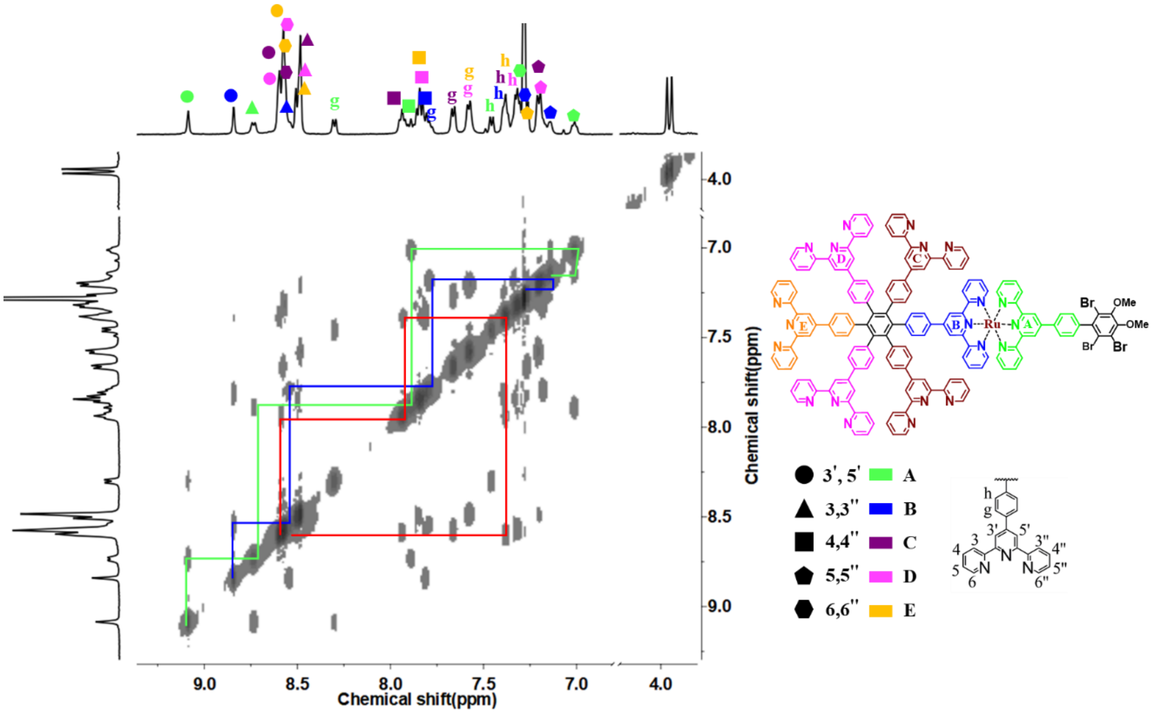

Figure S19. The 2D-NOESY spectrum of **B1** (500 MHz) in $CDCl_3$. Related to Figure 2.

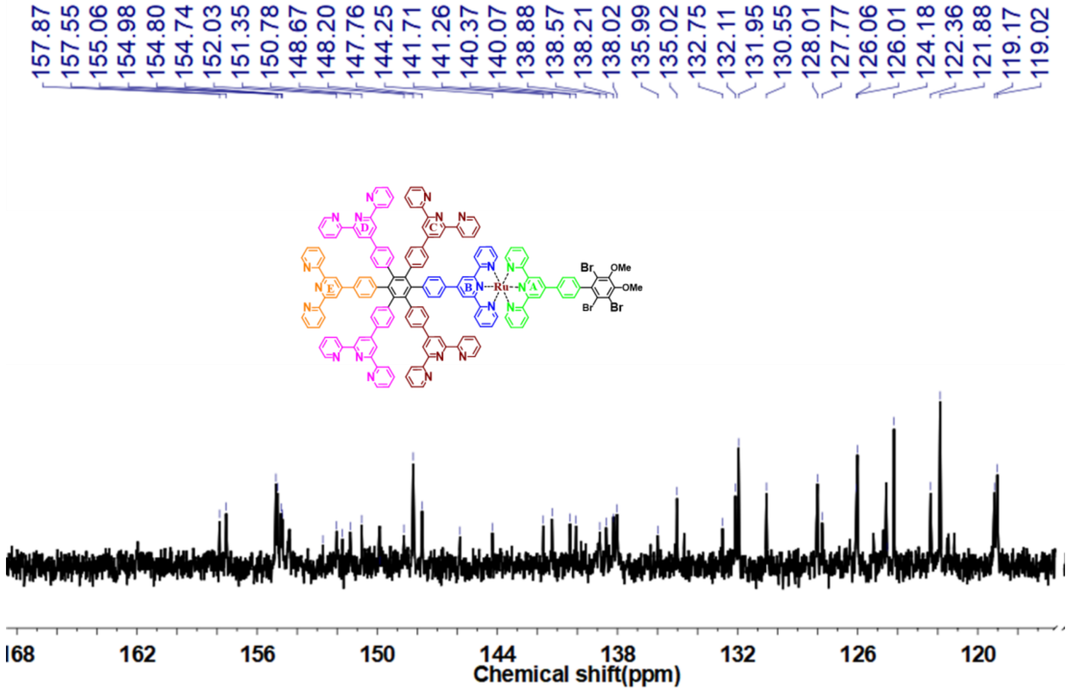

Figure S20. The ^{13}C NMR spectrum of **B1** (126 MHz) in $CDCl_3$. Related to Figure 2.

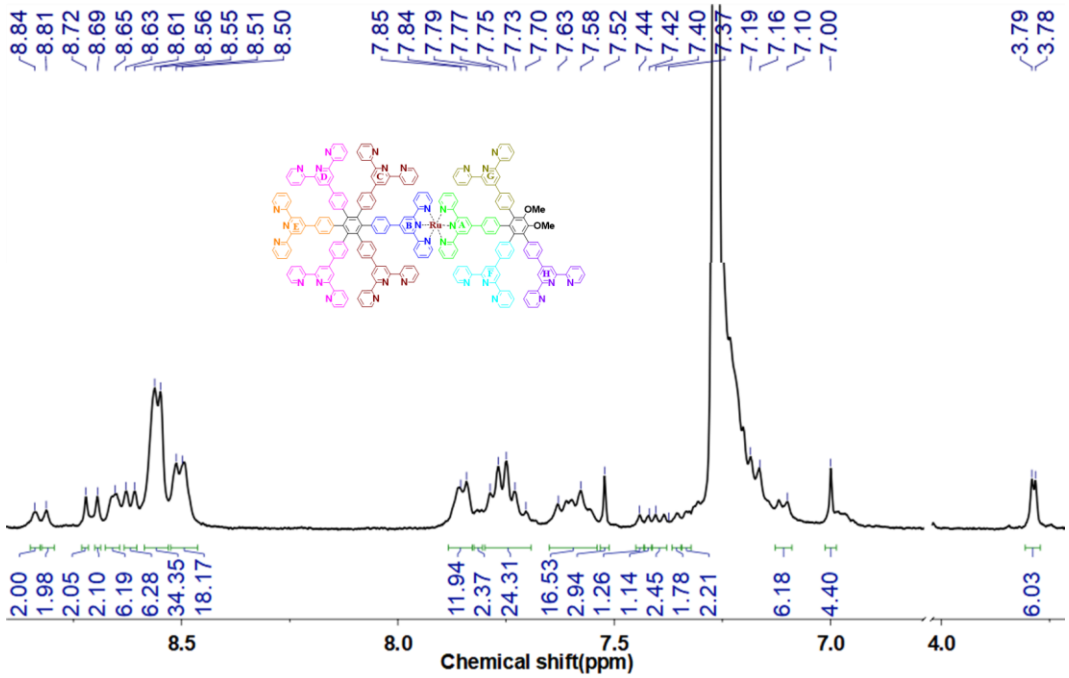

Figure S21. The ^{1}H NMR spectrum of **L1** (400 MHz) in $CDCl_3$. Related to Figure 2.

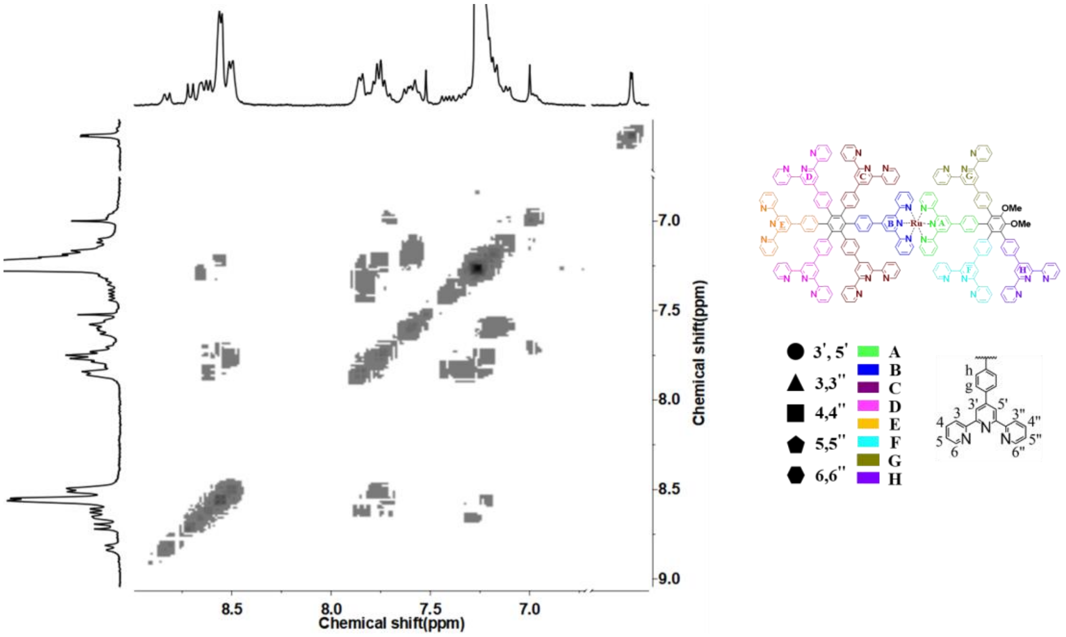

Figure S22. The 2D-COSY spectrum of **L1** (400 MHz) in $CDCl_3$. Related to Figure 2.

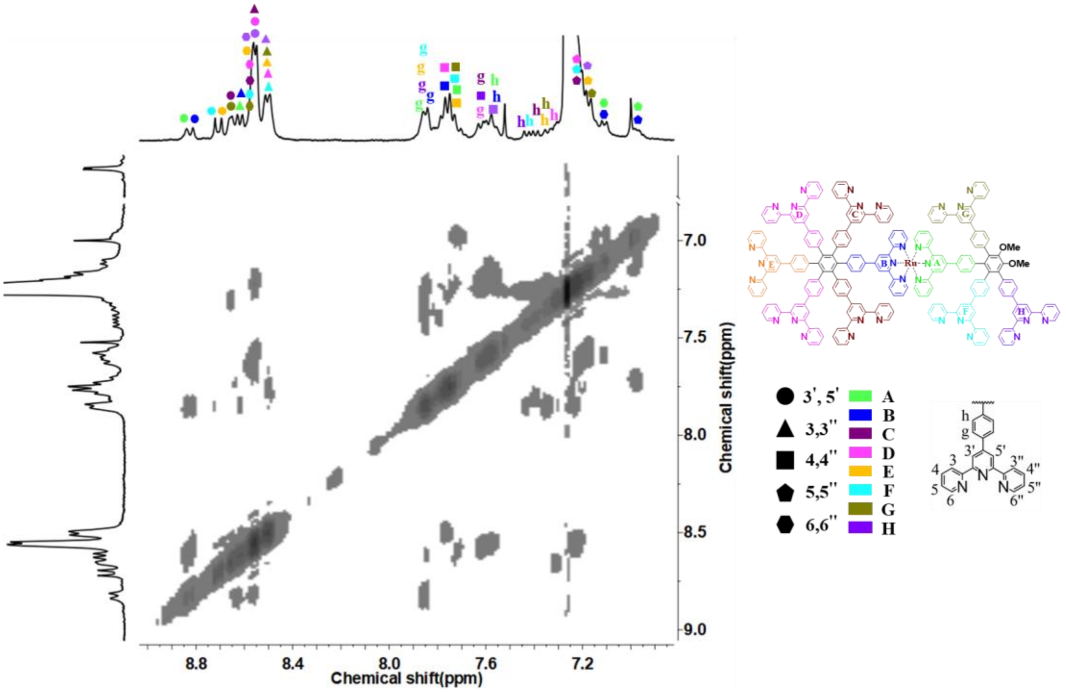

Figure S23. The 2D-NOESY spectrum of **L1** (400 MHz) in $CDCl_3$. Related to Figure 2.

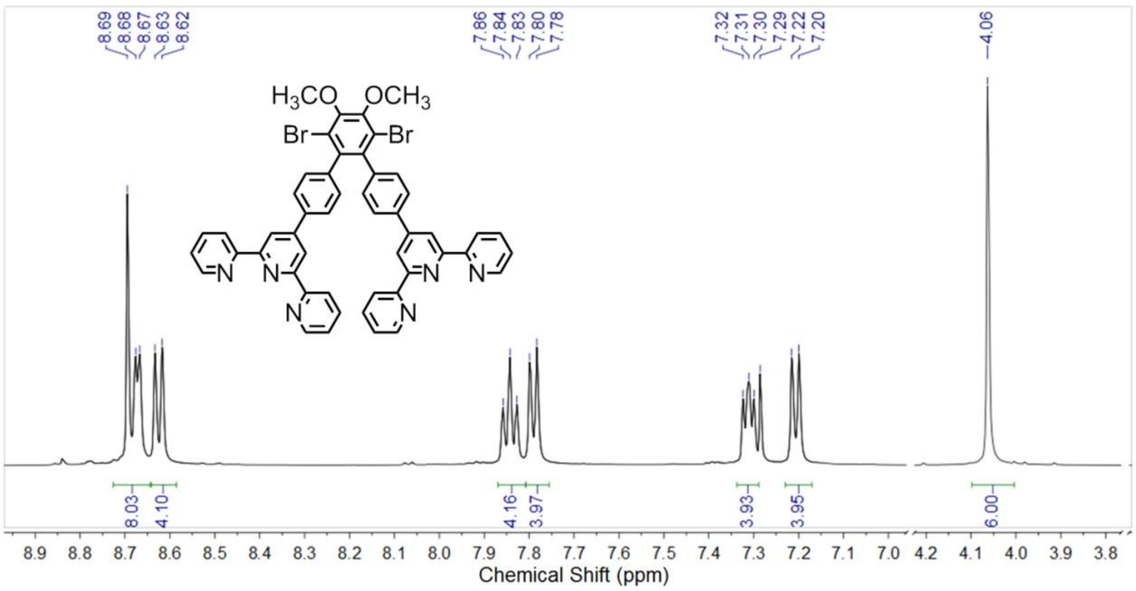

Figure S24. ^{1}H NMR spectrum of **S11** (500 MHz) in $CDCl_3$. Related to Figure 2.

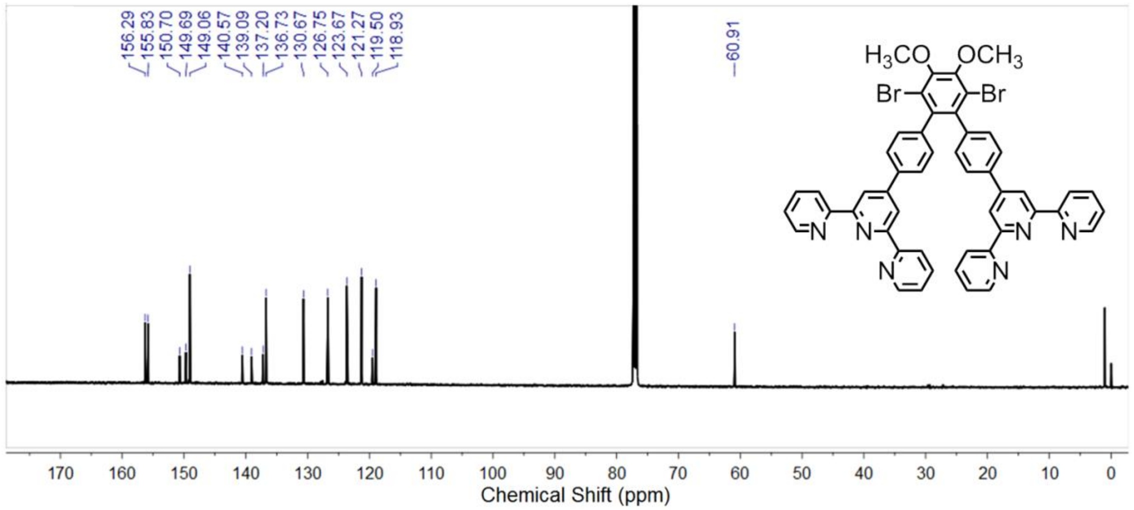

Figure S25. ^{13}C NMR spectrum of **S11** (126 MHz) in $CDCl_3$. Related to Figure 2.

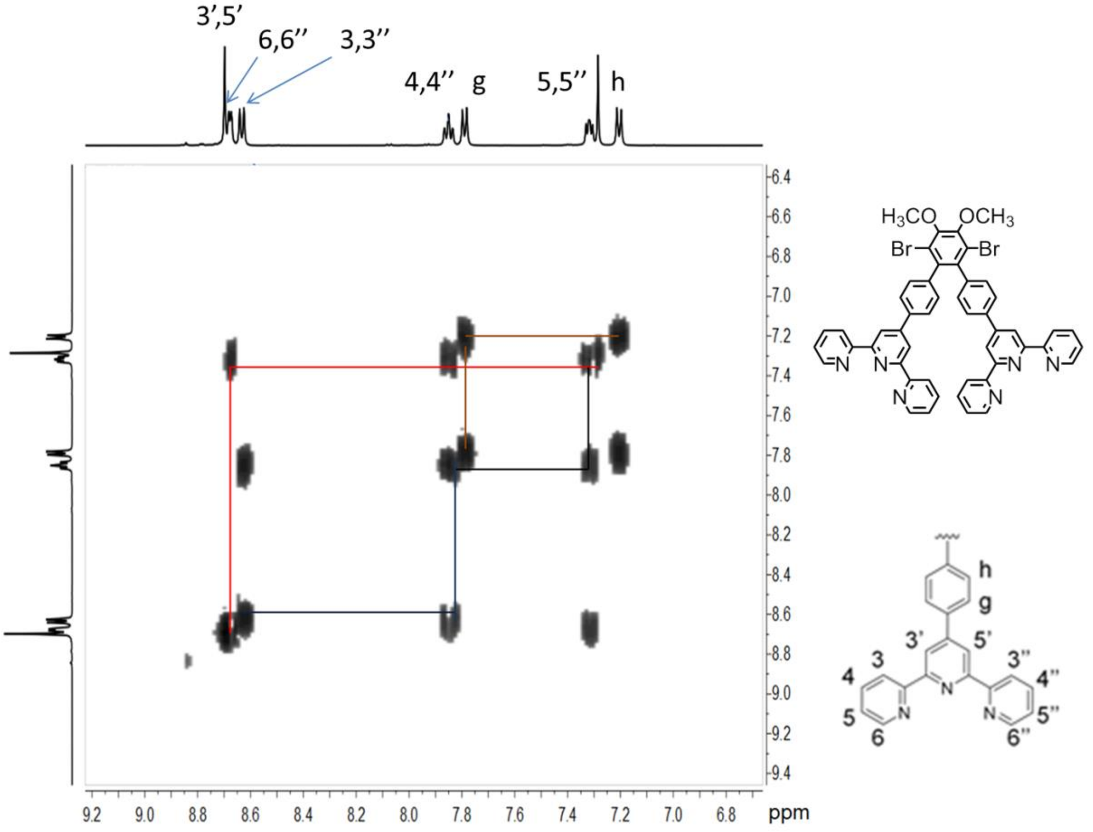

Figure S26. The 2D-COSY spectrum of **S11** (500 MHz) in $CDCl_3$. Related to Figure 2.

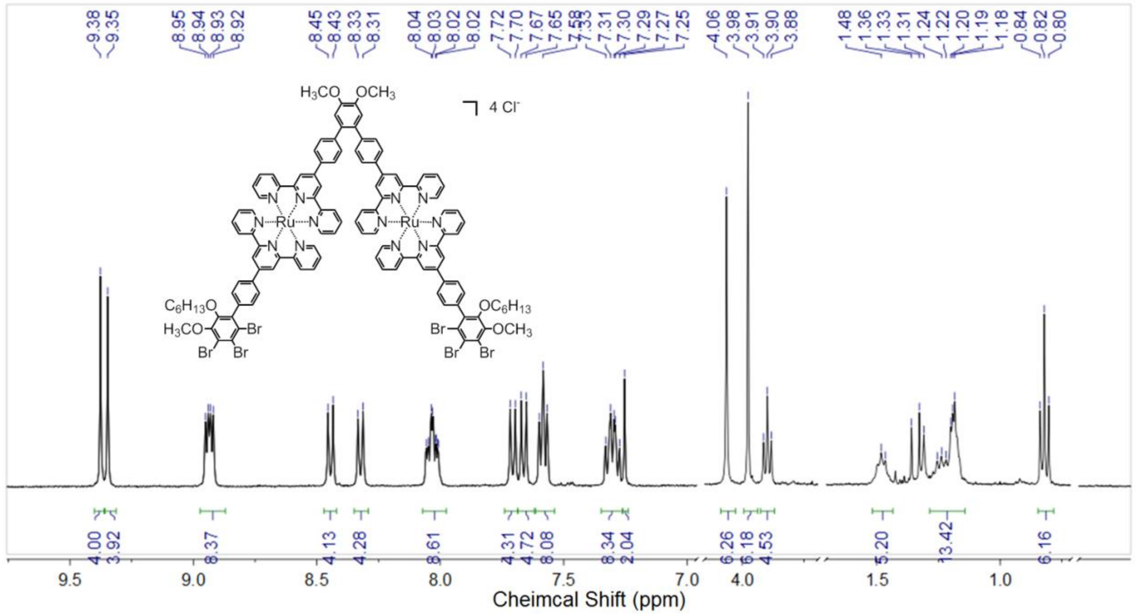

Figure S27. ^{1}H NMR spectrum of **B2** (400 MHz) in CD_3CN. Related to Figure 2.

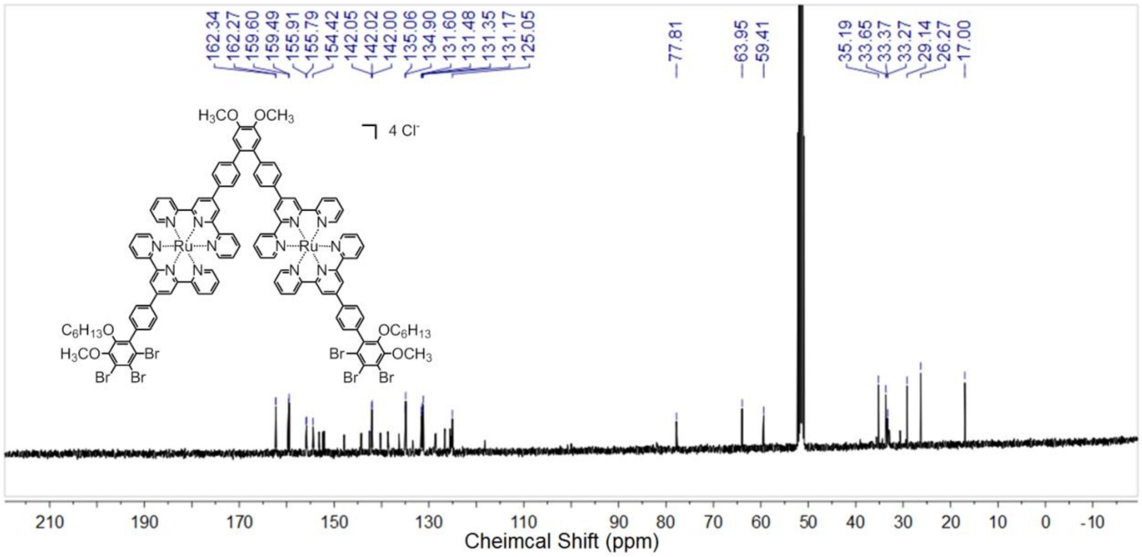

Figure S28. The ^{13}C NMR spectrum of **B2** (101 MHz) in CD3CN. Related to Figure 2.

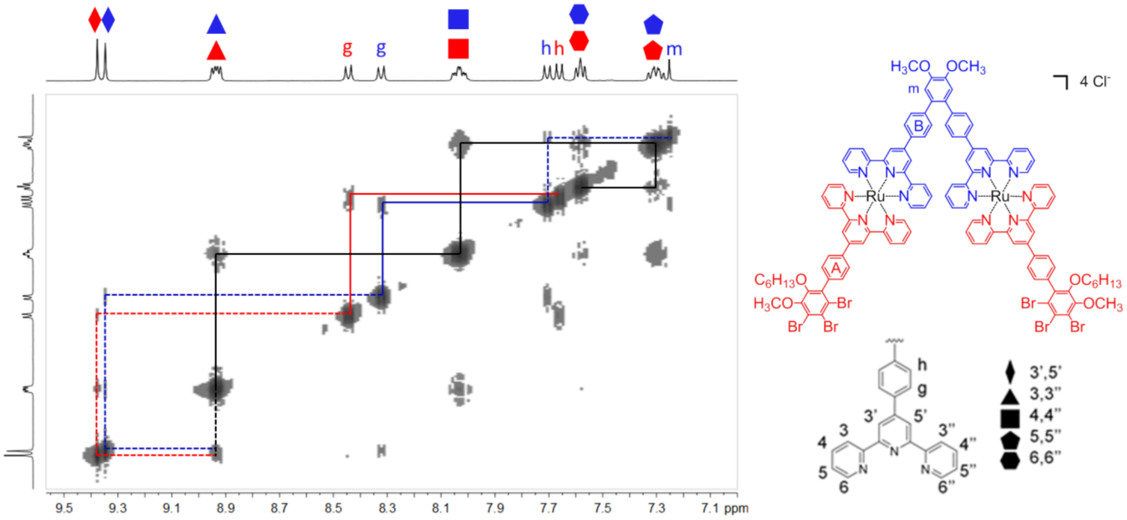

Figure S29. The NOESY spectrum of **B2** (500 MHz) in CD3CN. Related to Figure 2.

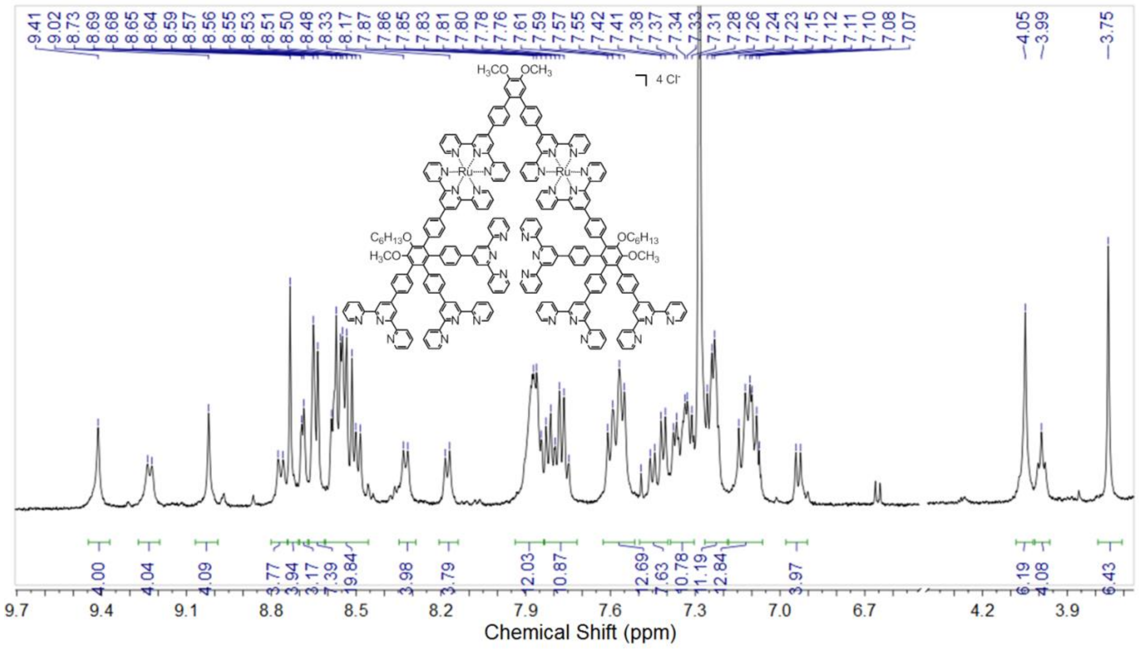

Figure S30. The ^{1}H NMR spectrum of **L2** (500 MHz) in CD_3CN. Related to Figure 2.

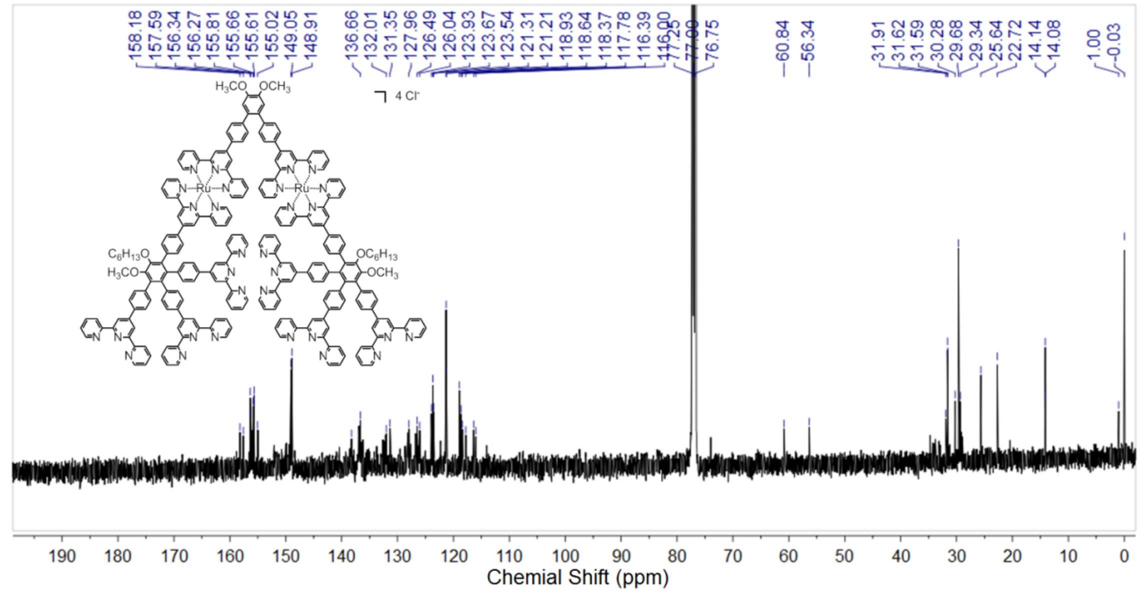

Figure S31. The ^{13}C NMR spectrum of **L2** (126 MHz) in CD_3CN. Related to Figure 2.

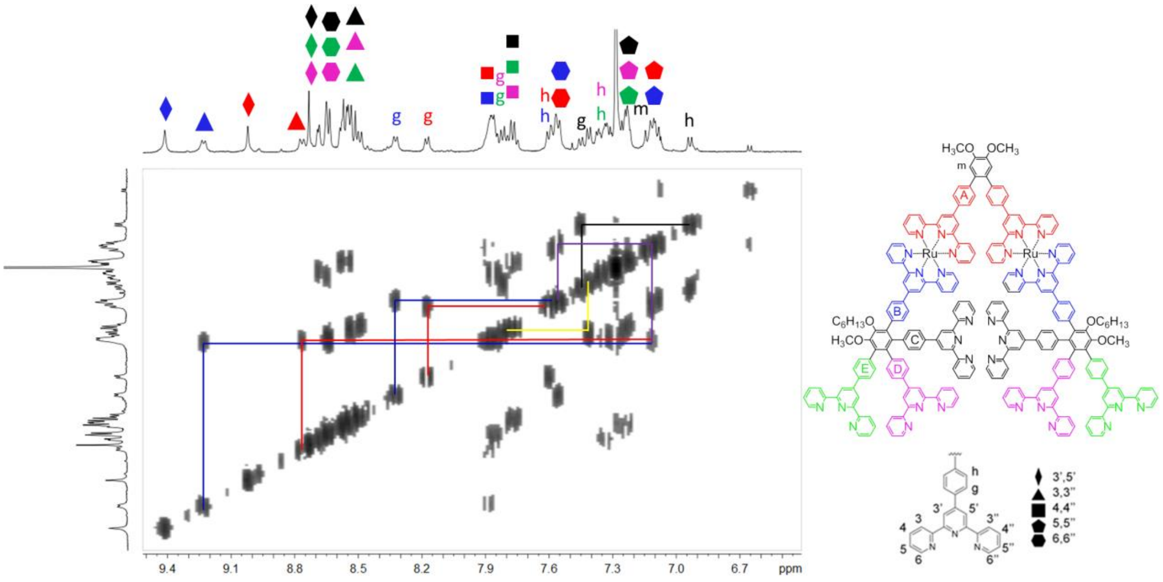

Figure S32. The 2D-COSY spectrum of **L2** (500 MHz) in CD_3CN. Related to Figure 2.

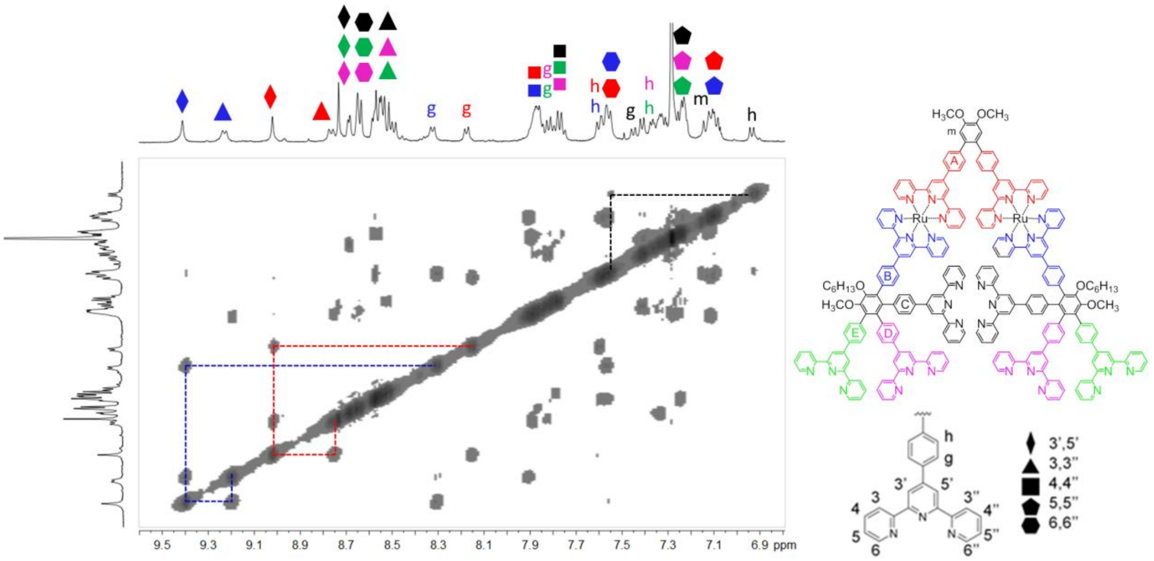

Figure S33. The NOESY spectrum of **L2** (500 MHz) in CD_3CN. Related to Figure 2.

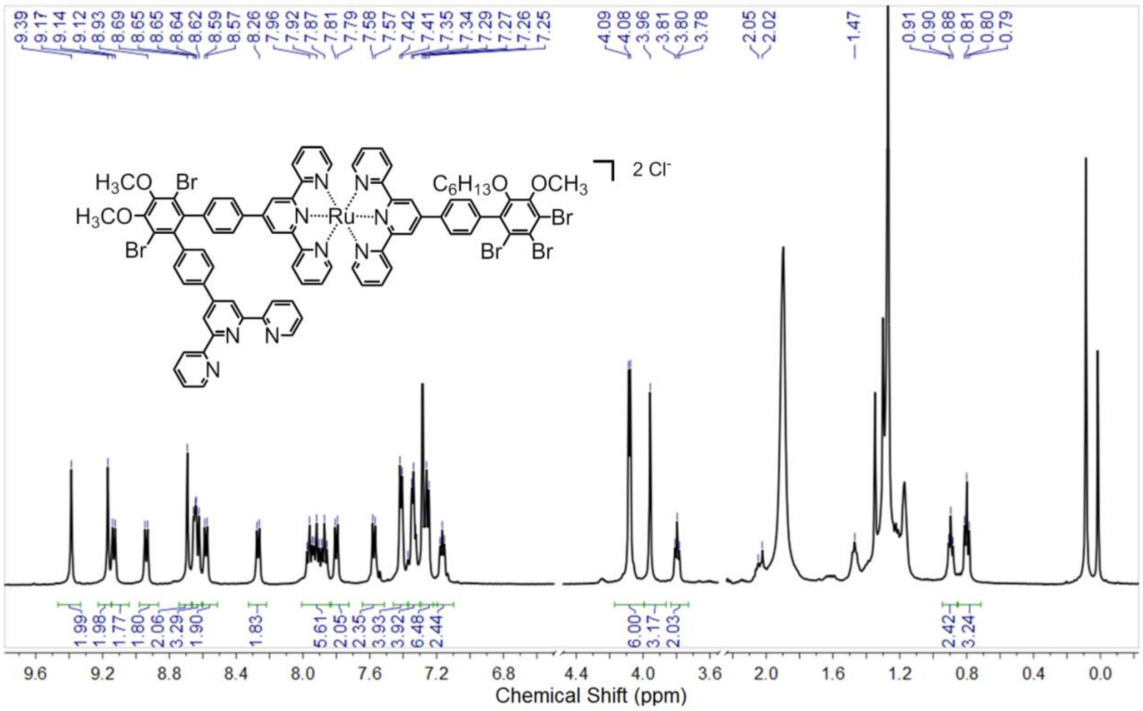

Figure S34. The ^{13}C NMR spectrum of **B3** (500 MHz) in $CDCl_3$. Related to Figure 2.

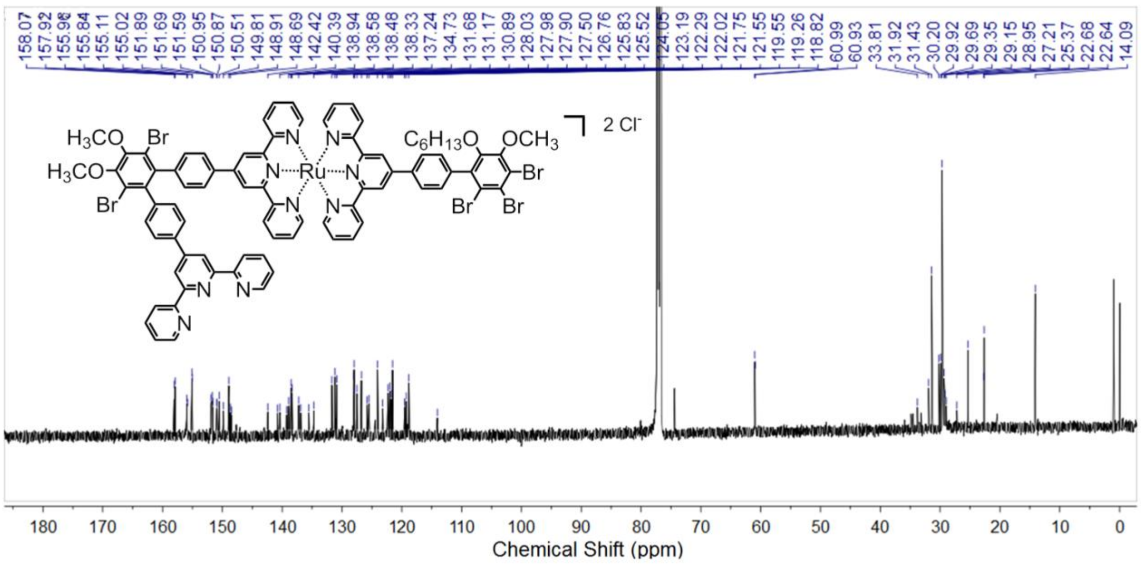

Figure S35. The ^{1}H NMR spectrum of **B3** (126 MHz) in $CDCl_3$. Related to Figure 2.

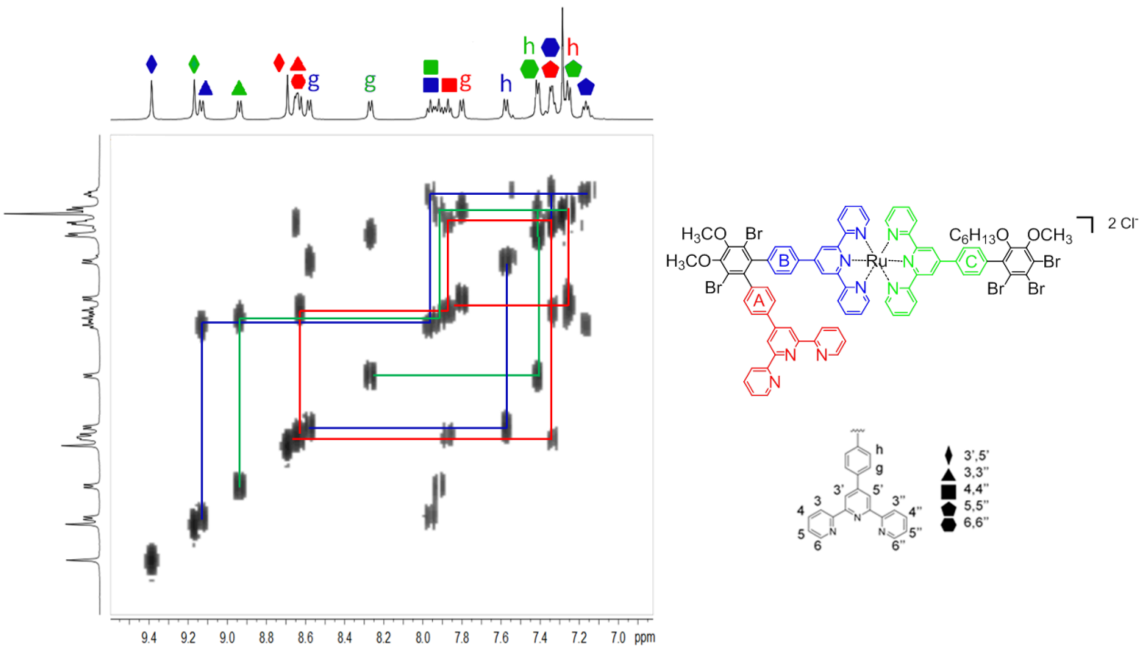

Figure S36. The COSY spectrum of **B3** (500 MHz) in $CDCl_3$. Related to Figure 2.

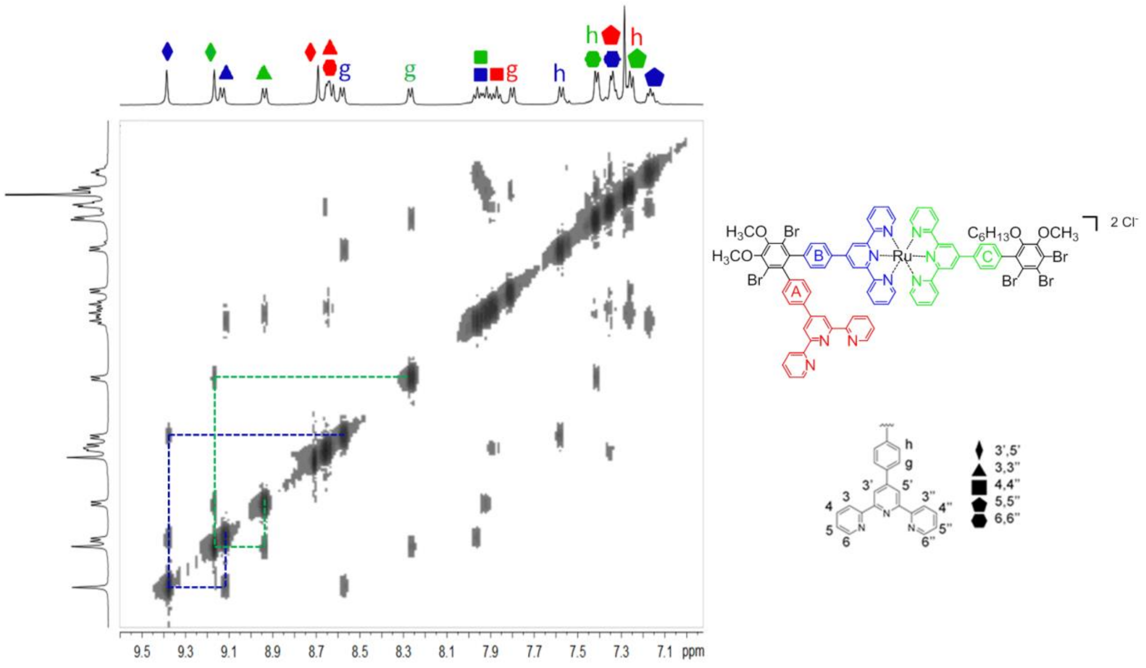

Figure S37. The NOESY spectrum of **B3**(500 MHz) in $CDCl_3$. Related to Figure 2.

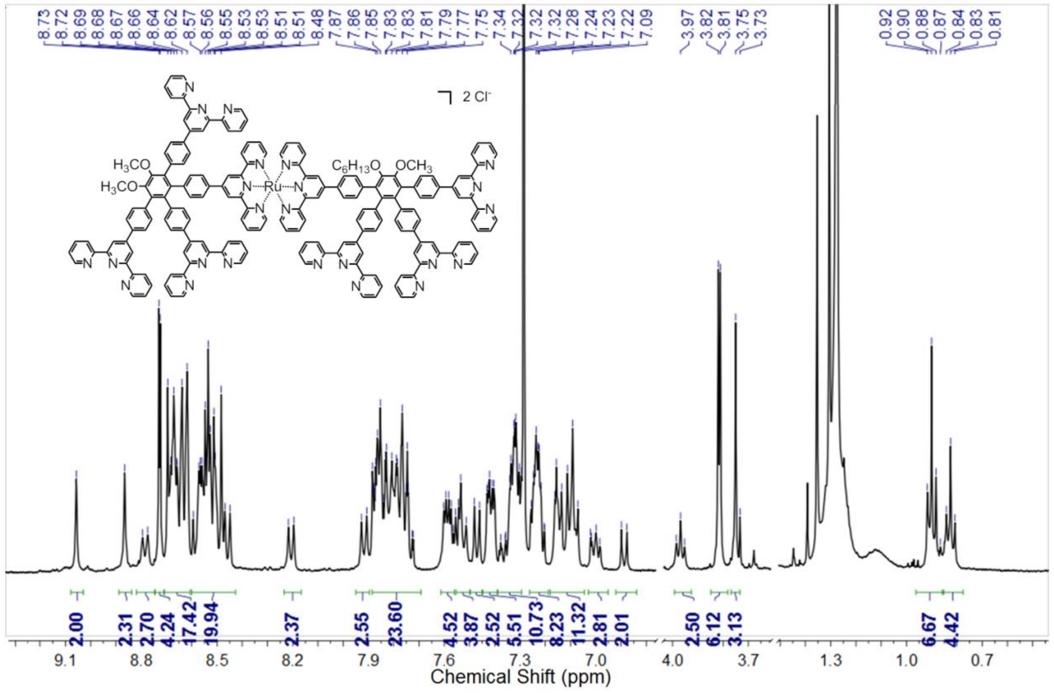

Figure S38. The ^{1}H NMR spectrum of **L3** (400 MHz) in $CDCl_3$. Related to Figure 2.

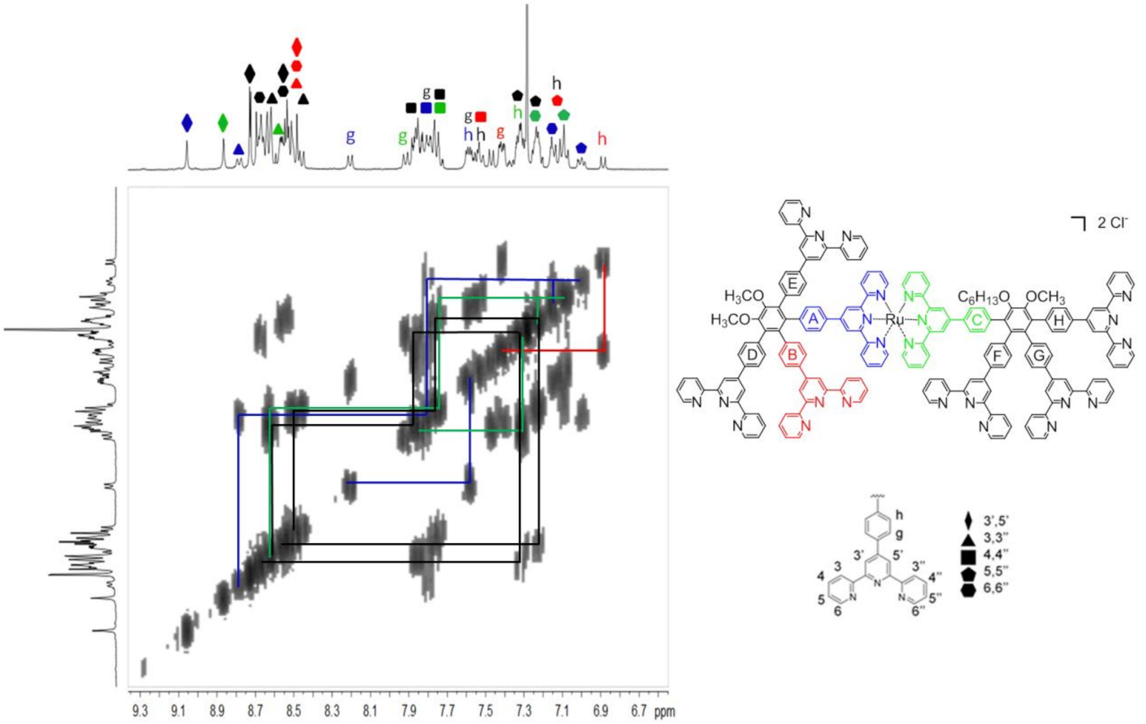

Figure S39. The 2D-COSY spectrum of **L3** (500 MHz) in $CDCl_3$. Related to Figure 2.

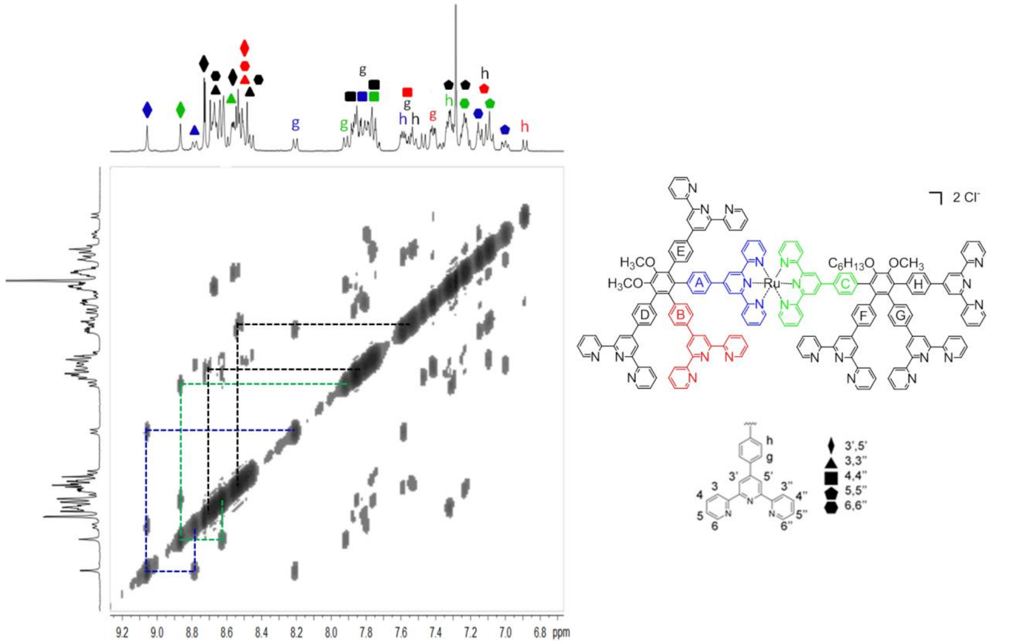

Figure S40. The 2D-NOESY spectrum of **L3** (500 MHz) in $CDCl_3$. Related to Figure 2.

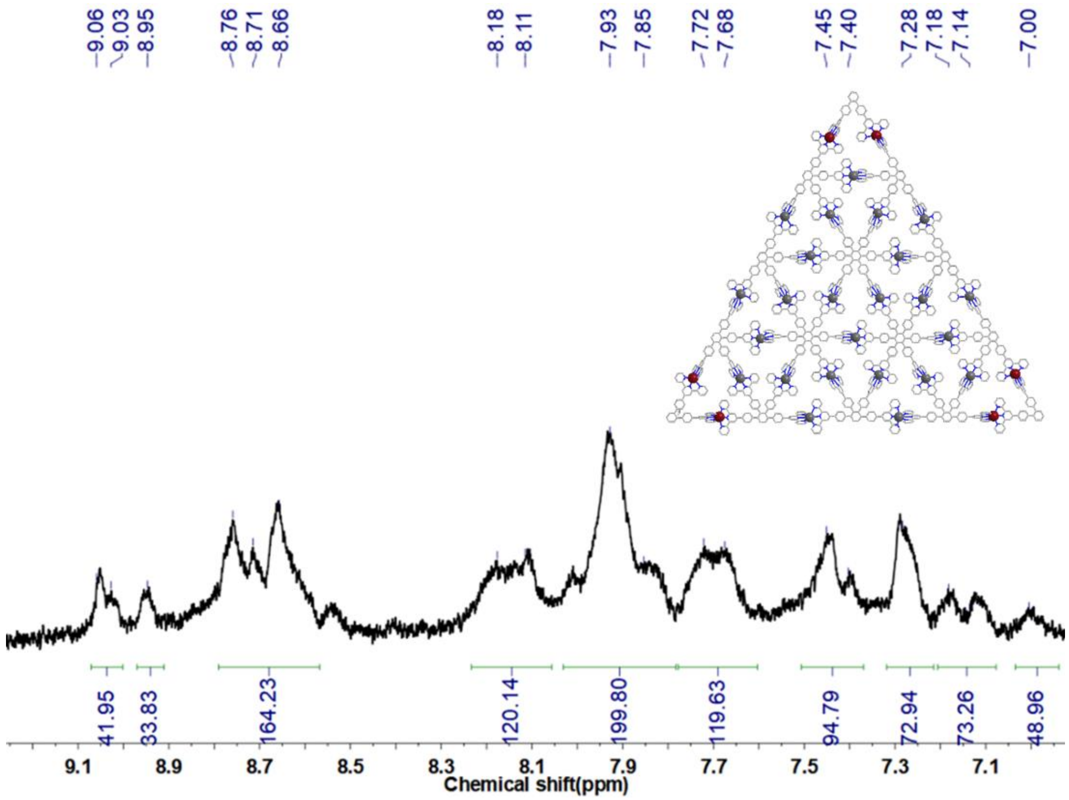

Figure S41. The ^{1}H NMR spectrum of G3 **PT-3** (500 MHz) in CD_3CN. Related to Figure 4.

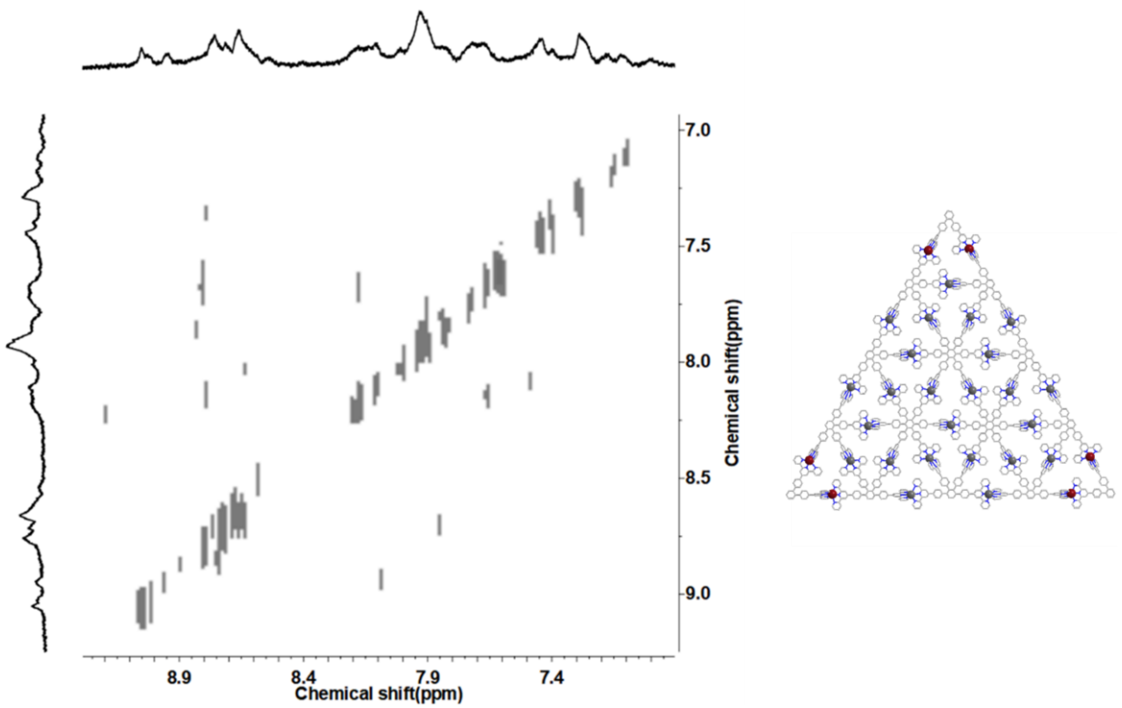

Figure S42. The 2D-COSY spectrum of G3 **PT-3** (500 MHz) in CD_3CN. Related to Figure 4.

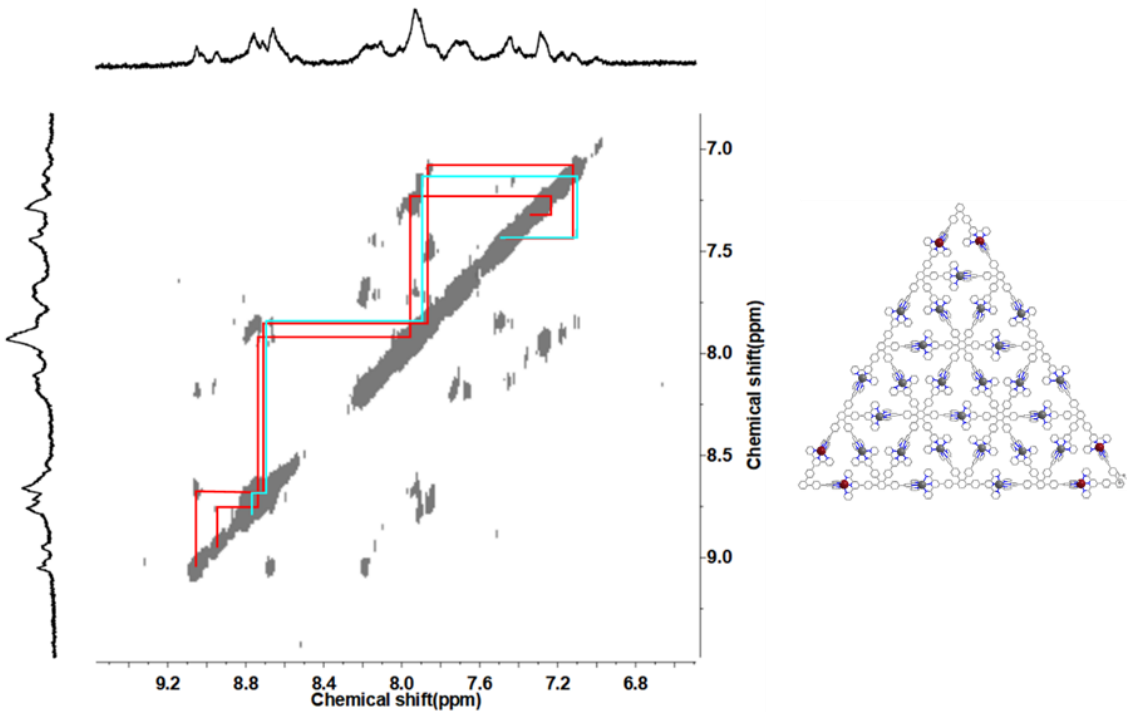

Figure S43. The 2D-NOESY spectrum of G3 **PT-3** (500 MHz) in CD_3CN. Related to Figure 4.

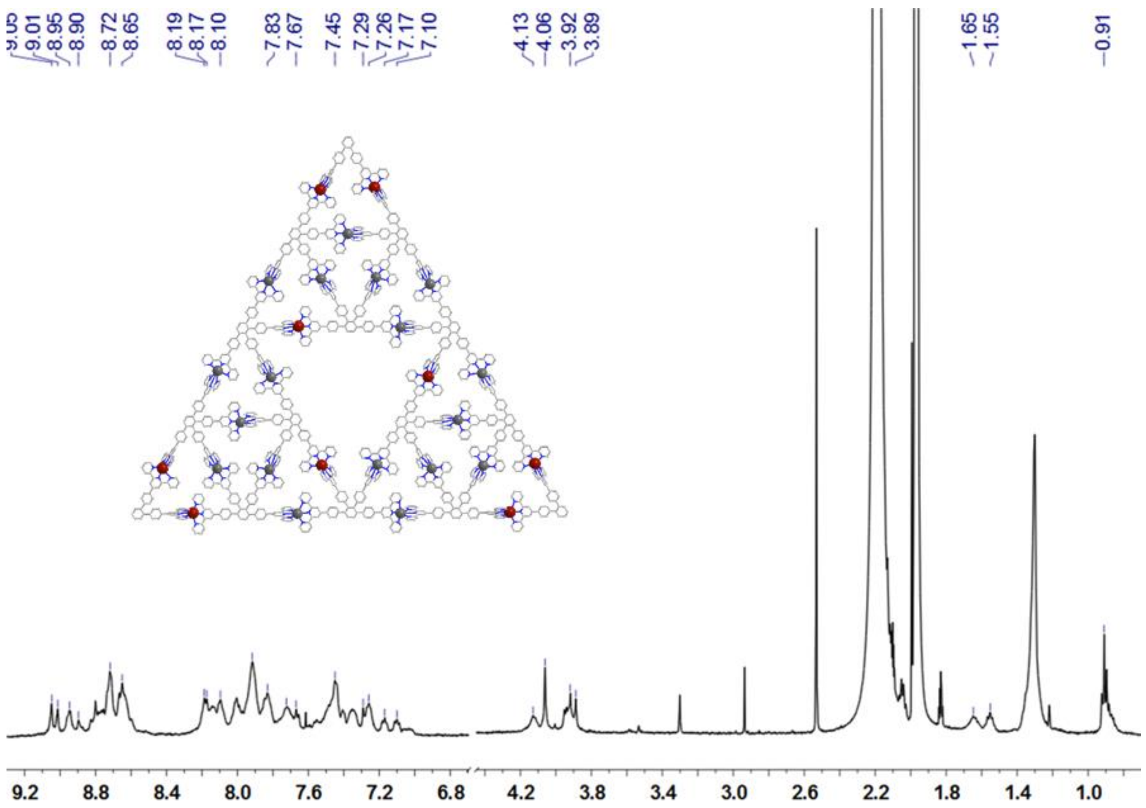

Figure S44. The ^{1}H NMR spectrum of G2 **ST** (500 MHz) in CD_3CN. Related to Figure 4.

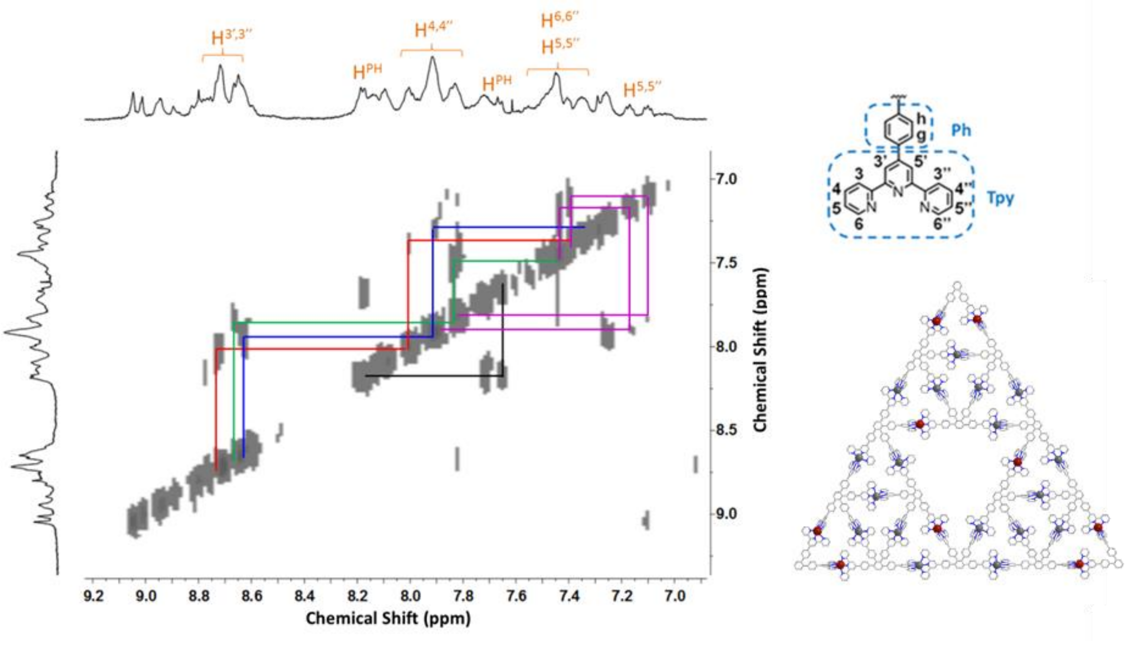

Figure S45. The 2D-COSY spectrum of G2 **ST** (500 MHz) in CD_3CN. Related to Figure 4.

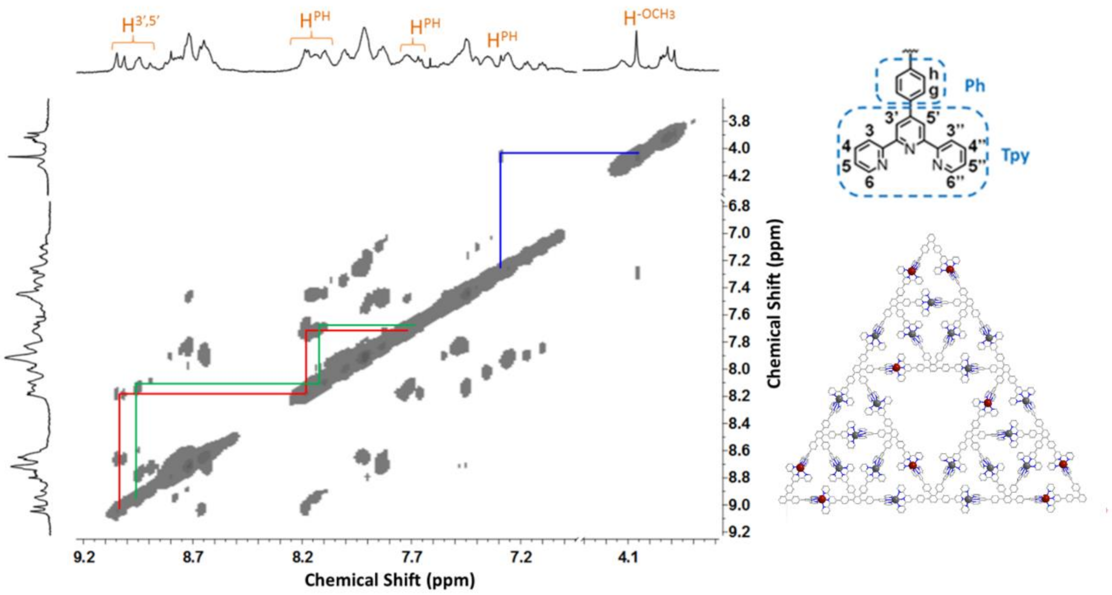

Figure S46. The 2D-NOESY spectrum of G2 **ST** (500 MHz) in CD_3CN. Related to Figure 4.

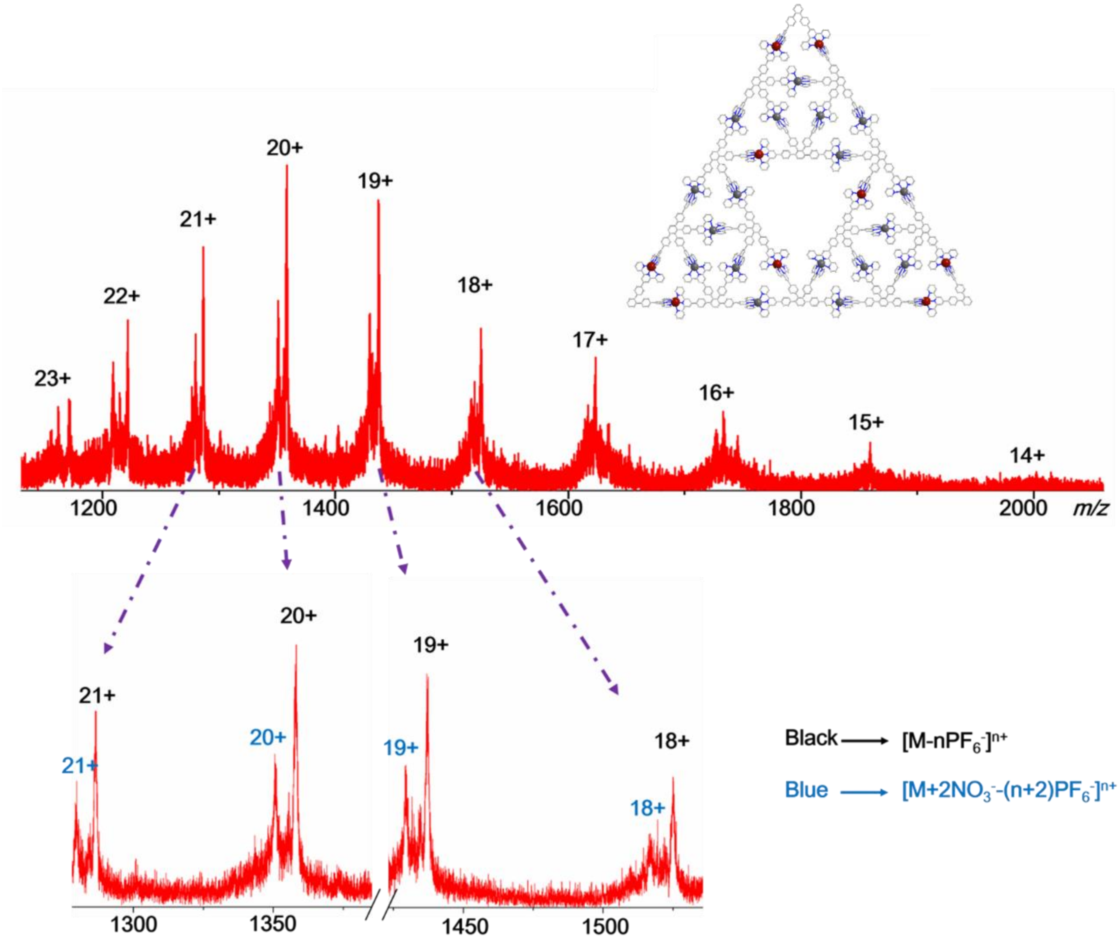

Figure S47. ESI-MS spectrum of G2 **ST** indicated the incomplete exchange of counterions (ion exchange was performed by adding excess amount of NH_4PF_6), the NO_3^- may held at the center of the little triangle structure (Ayme et al., 2012). Related to Figure 4.

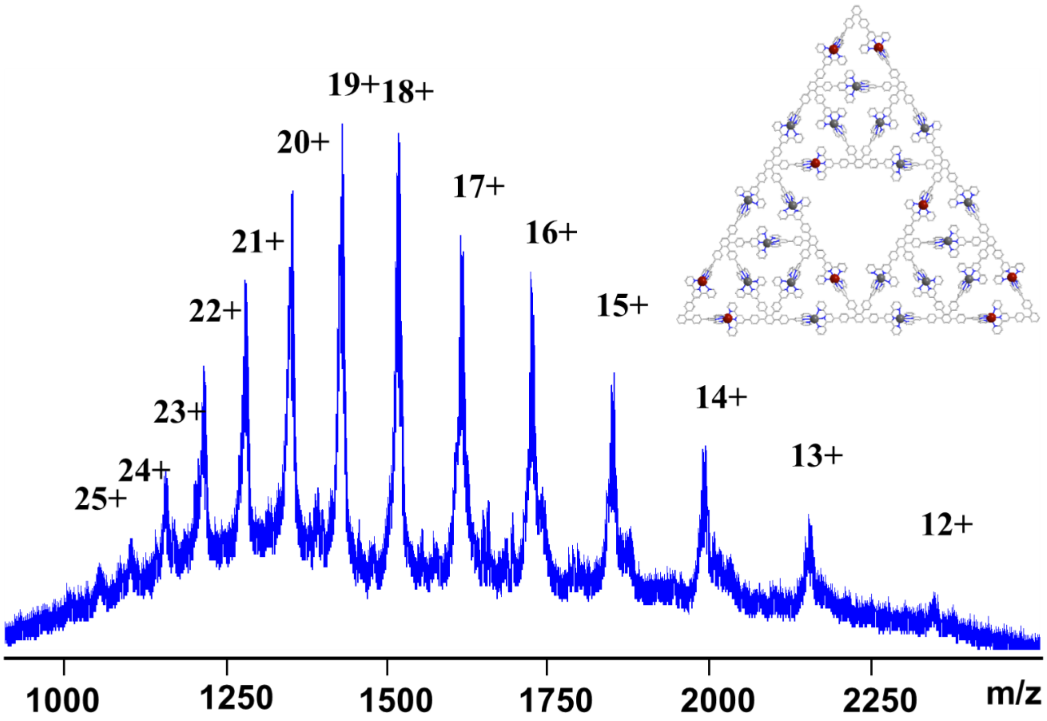

Figure S48. The ESI-MS spectrum of ligand G2 **ST** was obtained at low source and desolvation temperature. Therefore, supramolecules can form solvent adducts and/or salts adducts in ESI-MS (Sun et al., 2015). Related to Figure 4.

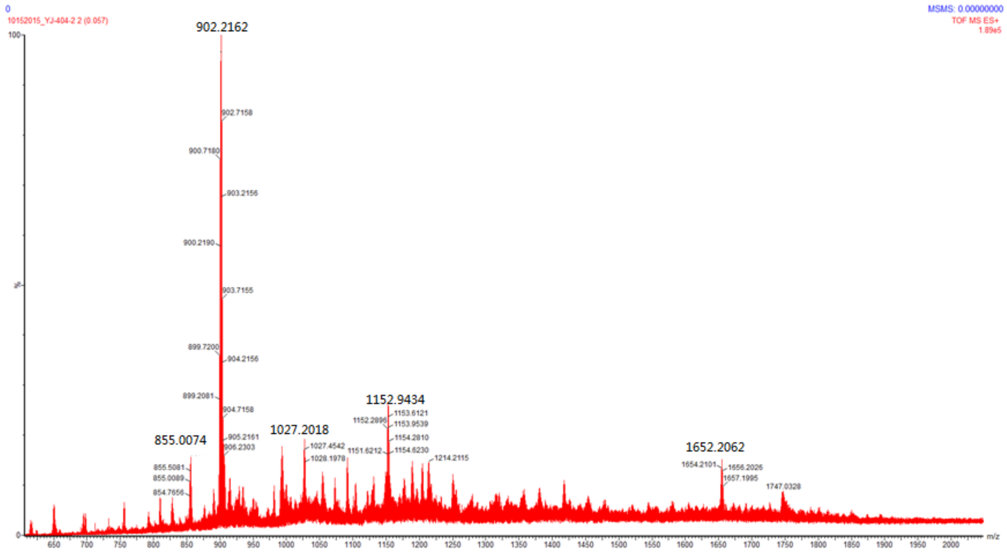

Figure S49. The ESI-MS Spectrum of assembled products by metal-organic ligand **L2** and K-shaped tetra*kis*-terpyridine **4** with Cd^{2+}, the resultant G2 **ST** was unidentified by ESI-MS. Related to Figure 4.

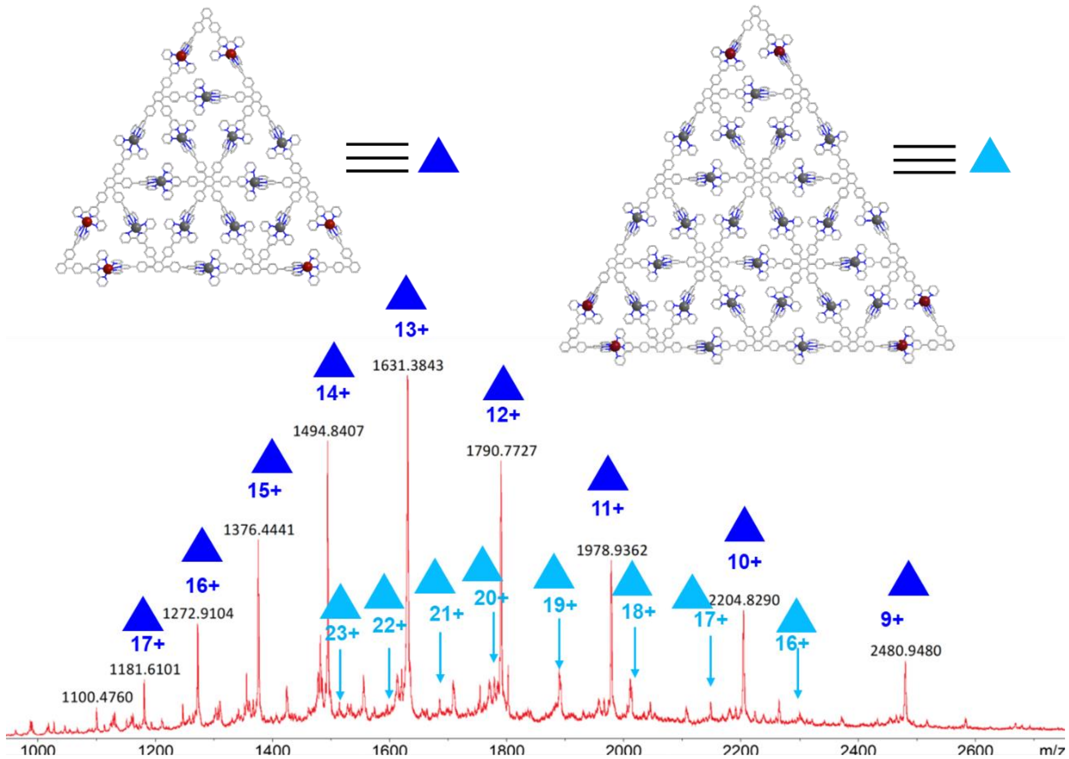

Figure S50. The ESI-MS Spectrum of assembled products by metal-organic ligand **L2**, K-shaped tetra*kis*-terpyridine **4** and star shaped **5** with Cd^{2+}. Only the G2 **PT** metallo-triangle architecture and a trace amount of G3 **PT** could be obtained. Related to Figure 4.

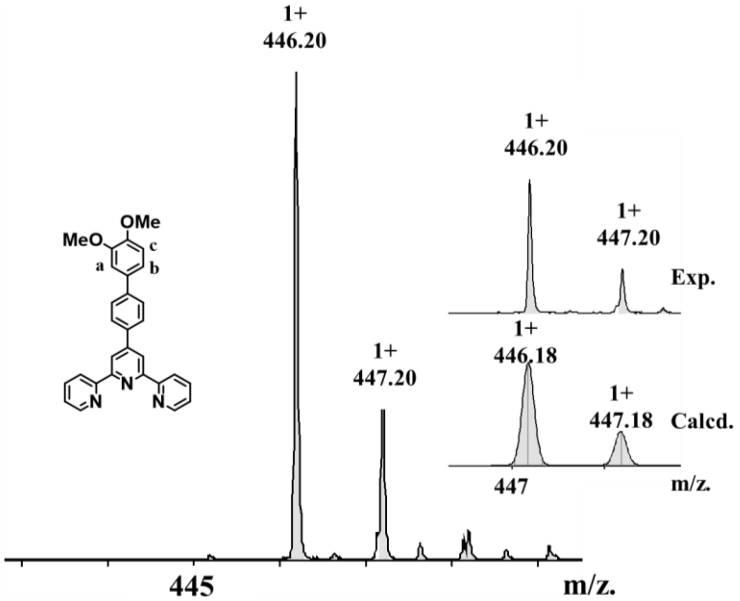

Figure S51. The ESI-MS spectrum of ligand **S6**. Related to Figure 2.

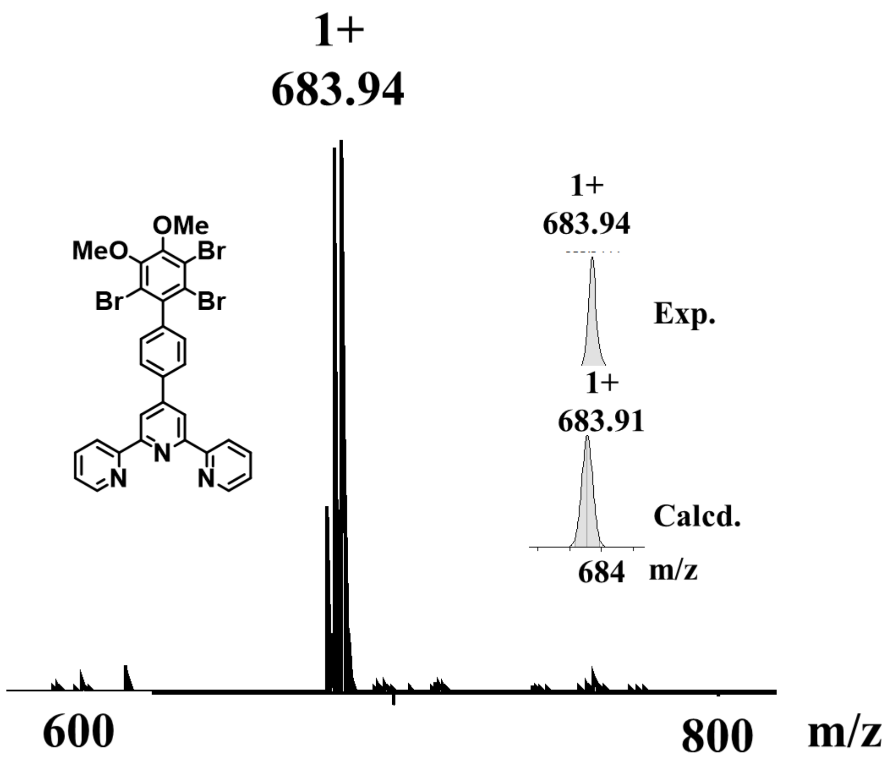

Figure S52. The ESI-MS spectrum of ligand **S7**. Related to Figure 2.

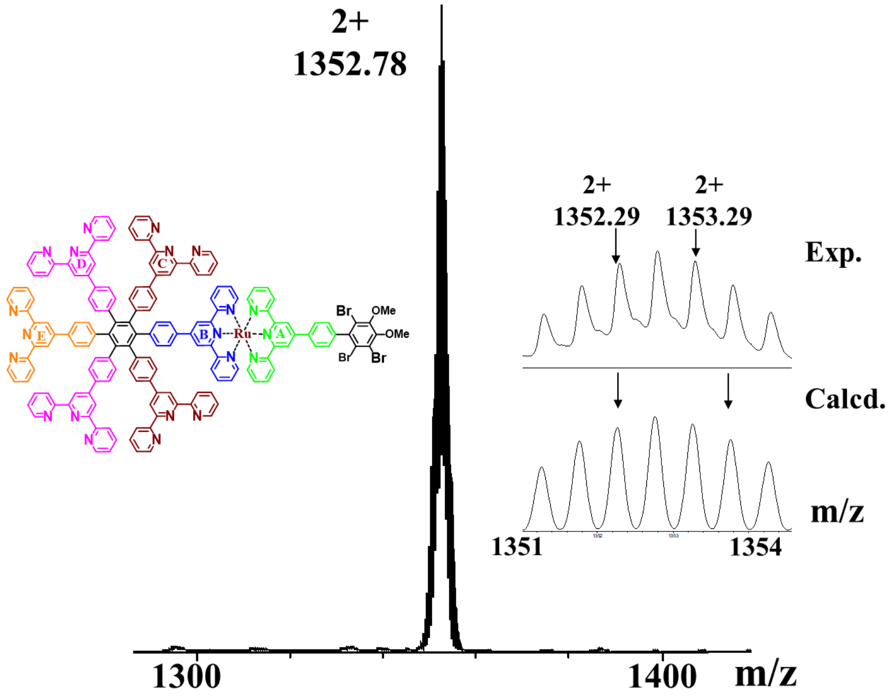

Figure S53. ESI-MS spectrum of ligand **6**. Related to Figure 2.

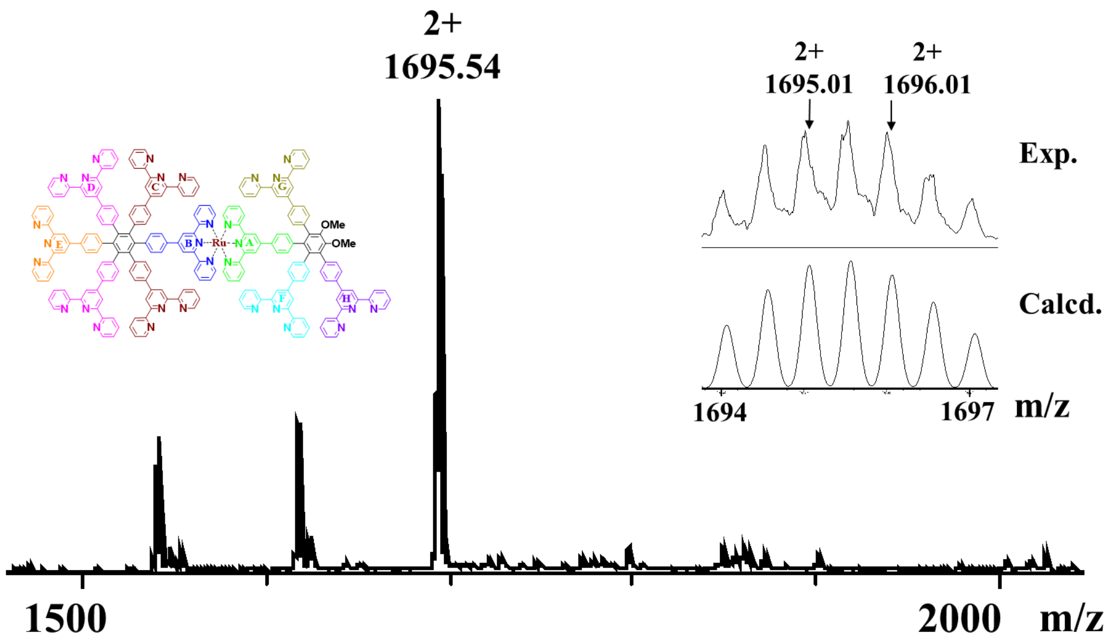

Figure S54. ESI-MS spectrum of ligand **L1**. Related to Figure 2.

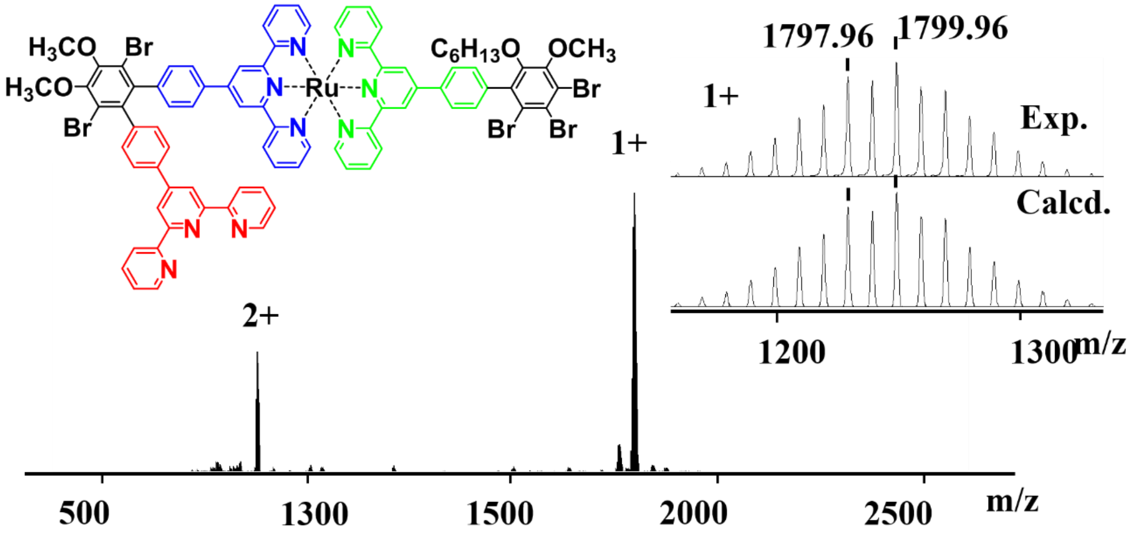

Figure S55. The HR-MS spectrum of complex **10**. Related to Figure 2.

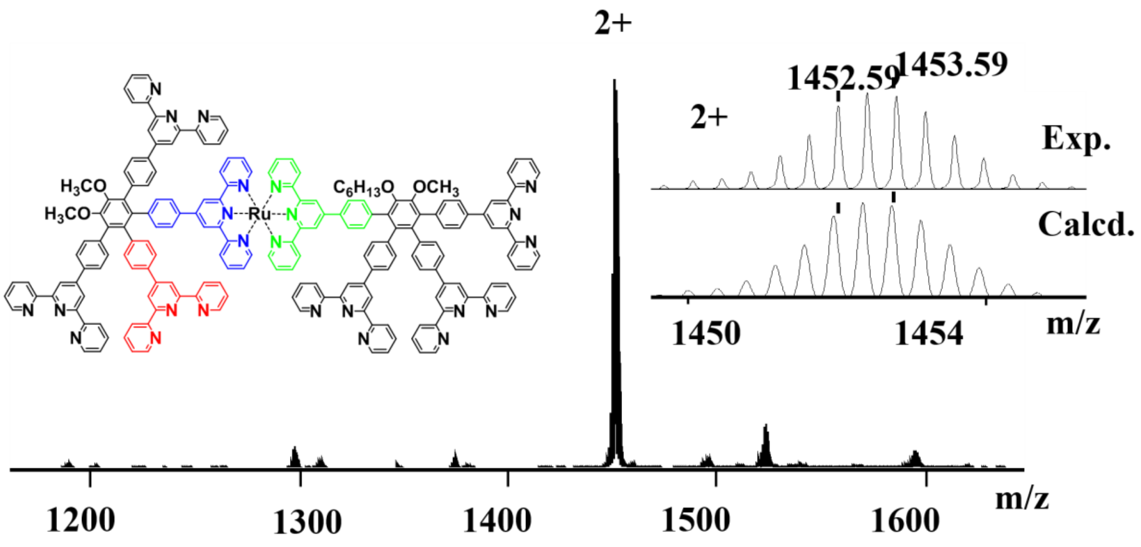

Figure S56. The MS spectrum of ligand **L3**. Related to Figure 2.

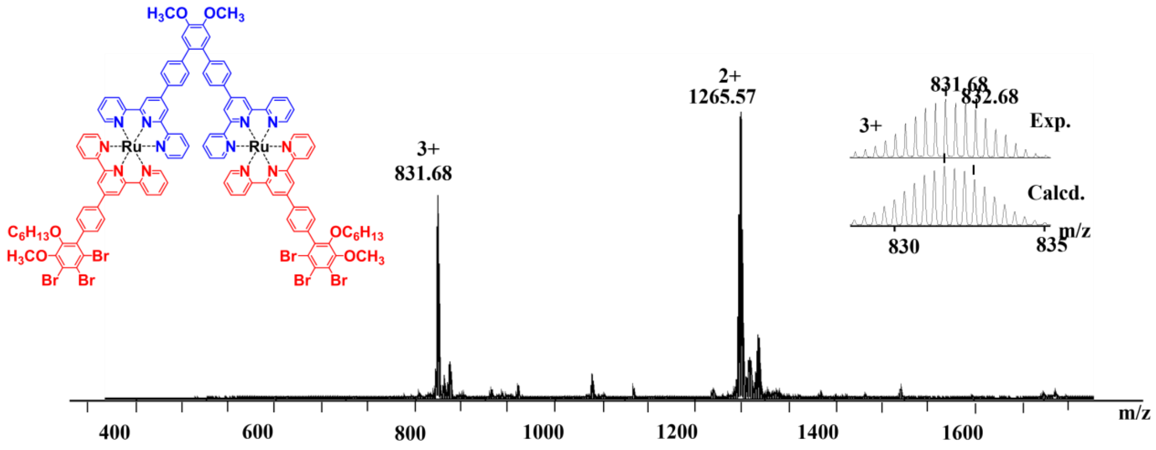

Figure S57. The ESI-MS spectrum of complex **8**. Related to Figure 2.

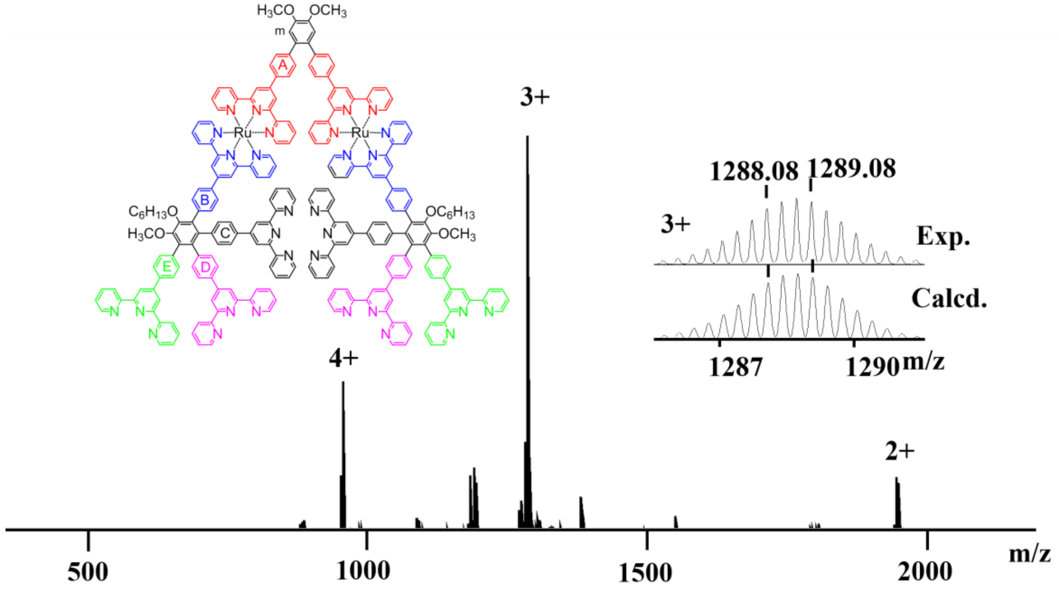

Figure S58. The ESI-MS spectrum of ligand **L2**. Related to Figure 2.

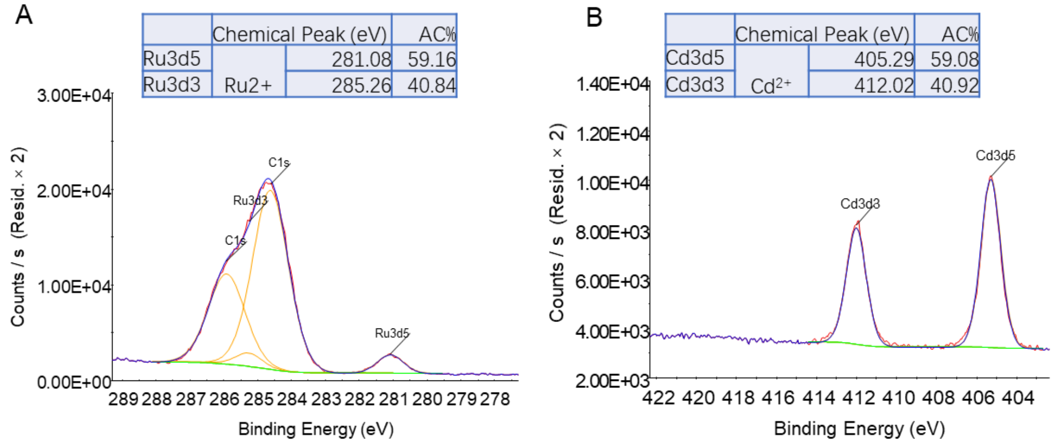

Figure S59. X-ray photoelectron spectrum of (A) Ru and (B) Cd of Pascal's Triangle G3 **PT**. Related to Figure 4.

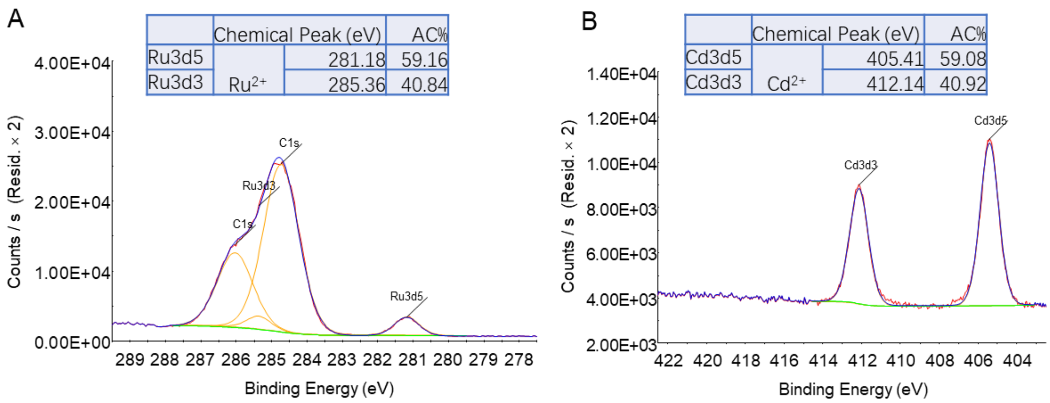

Figure S60. X-ray photoelectron spectrum of (A) Ru and (B) Cd of Sierpiński Triangle G2 **ST**. Related to Figure 4.

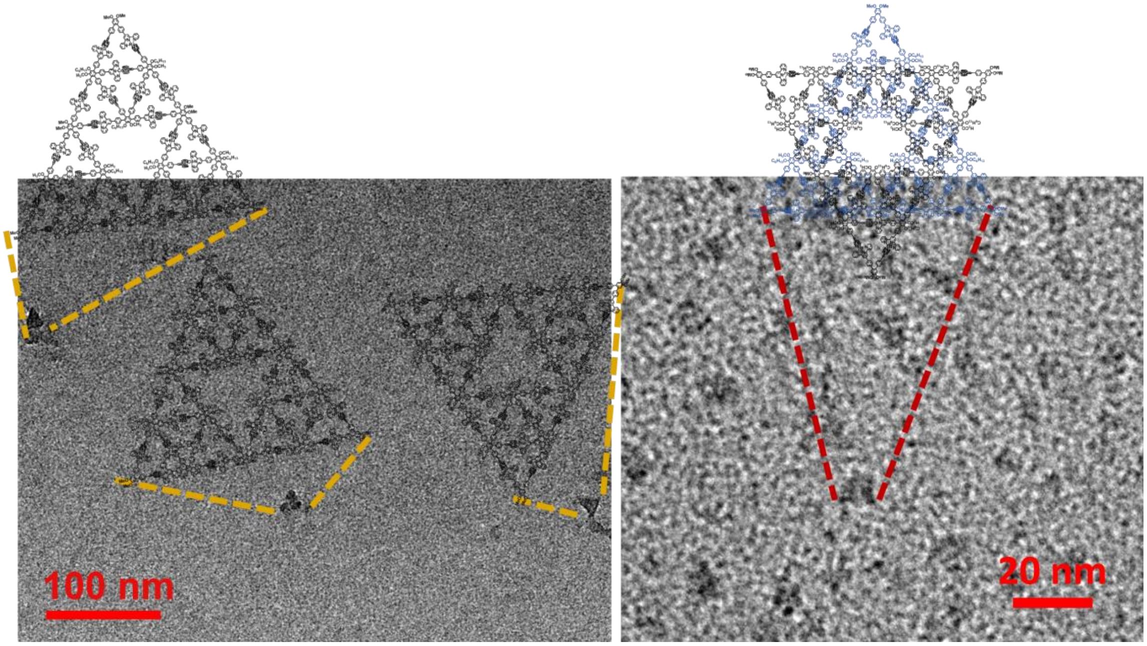

Figure S61. The TEM image of Sierpiński Triangle G2 **ST**. The architecture was observed as a triangle-shadow pattern and the length of sides that was comparable with the size of 11.6±0.5 nm calculated from molecular modeling. The overlapping of two triangles generated several Star-of-David patterns as well. Related to Figure 5.

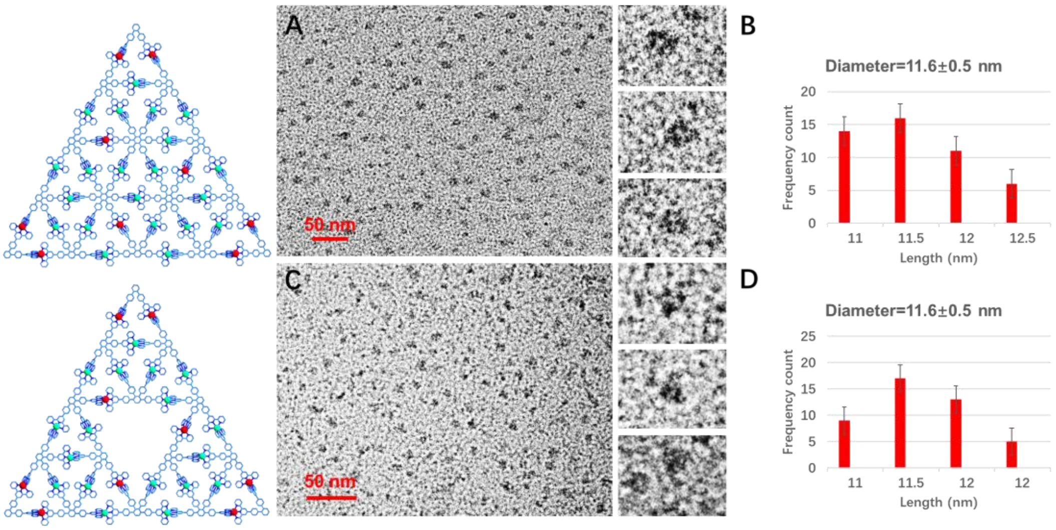

Figure S62. (A) TEM images of Pascal's Triangle G3 **PT** and (C) Sierpiński Triangle G2 **ST**; (B) TEM statistical size distributions of G3 **PT** and (C) G2 **ST**. Related to Figure 5.

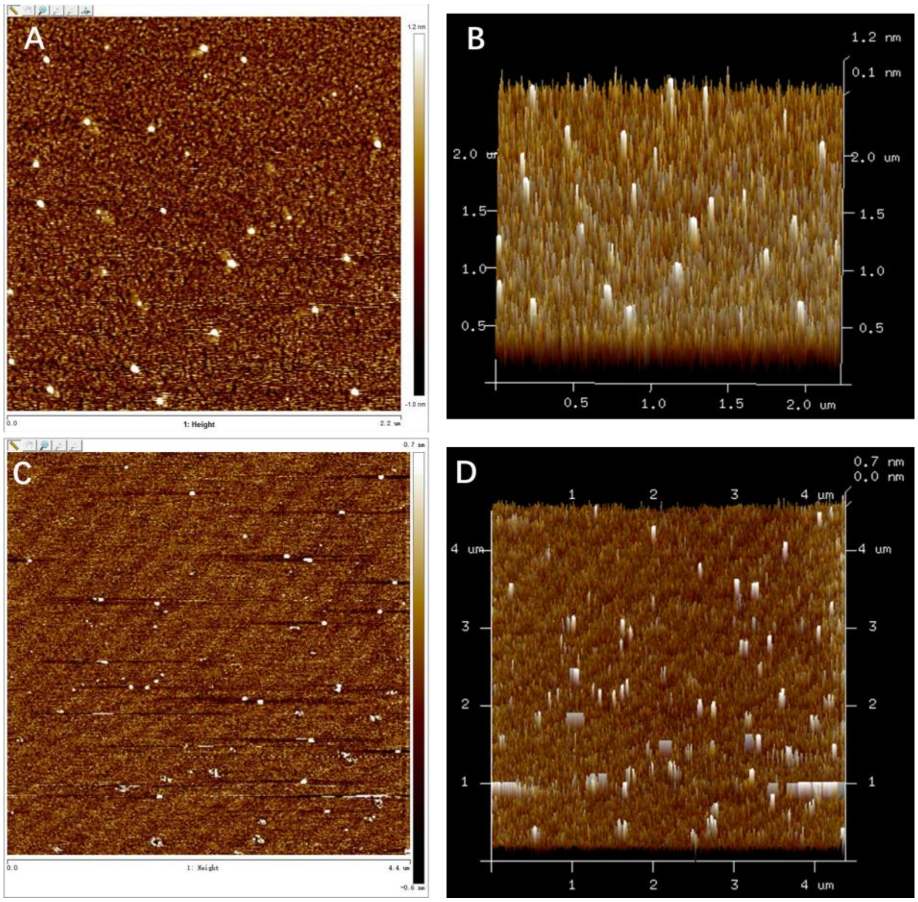

Figure S63. (A,B) AFM images of Pascal's Triangle G3 **PT** and (C,D) Sierpiński Triangle G2 **ST**. Related to Figure 5.

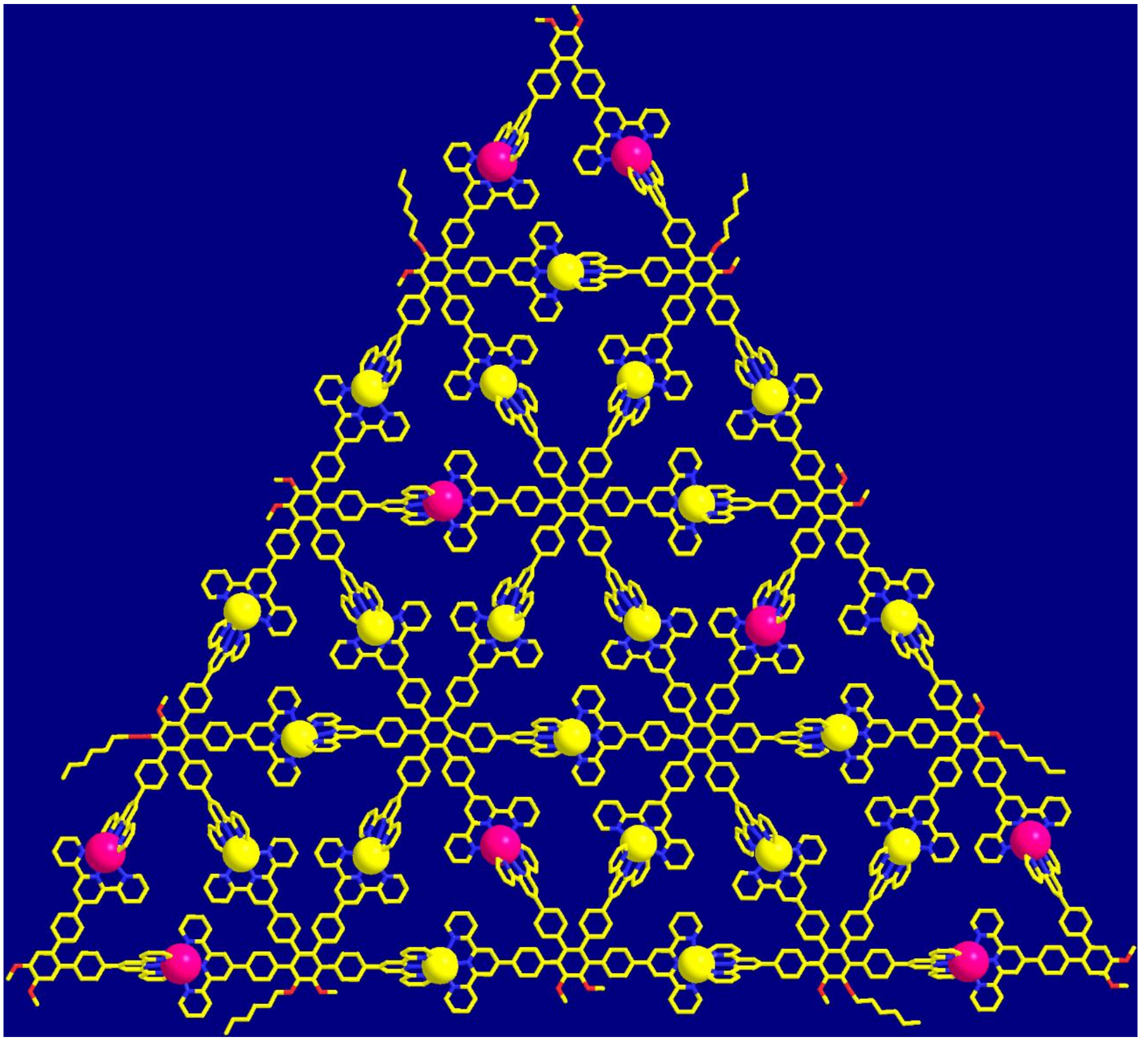

Figure S64. Representative energy-minimized structure from molecular modeling of G3 **PT**. Related to Figure 5.

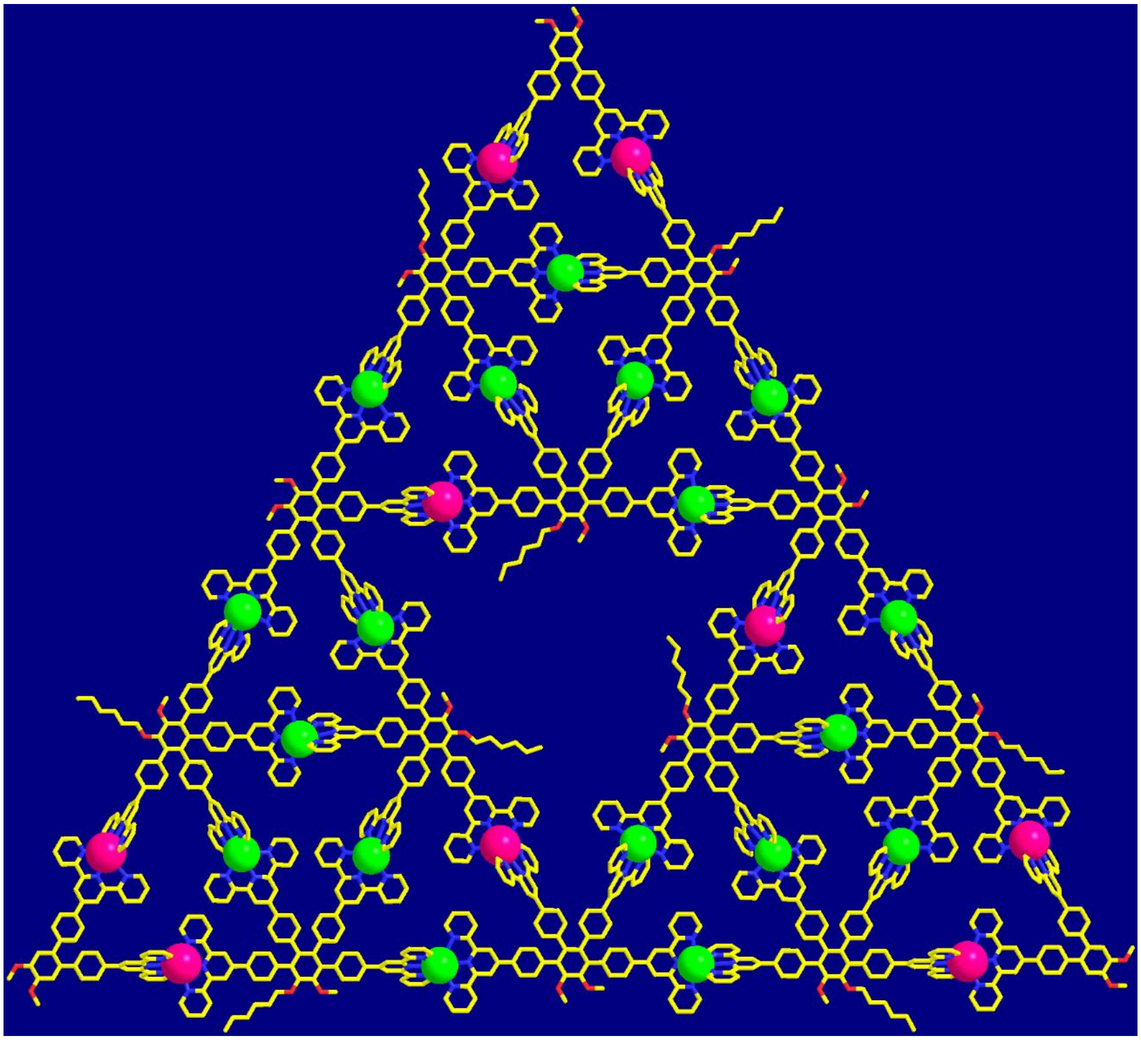

Figure S65. Representative energy-minimized structure from molecular modeling of G2 **ST**. Related to Figure 5.

Synthesis of the Ligands and Complexes

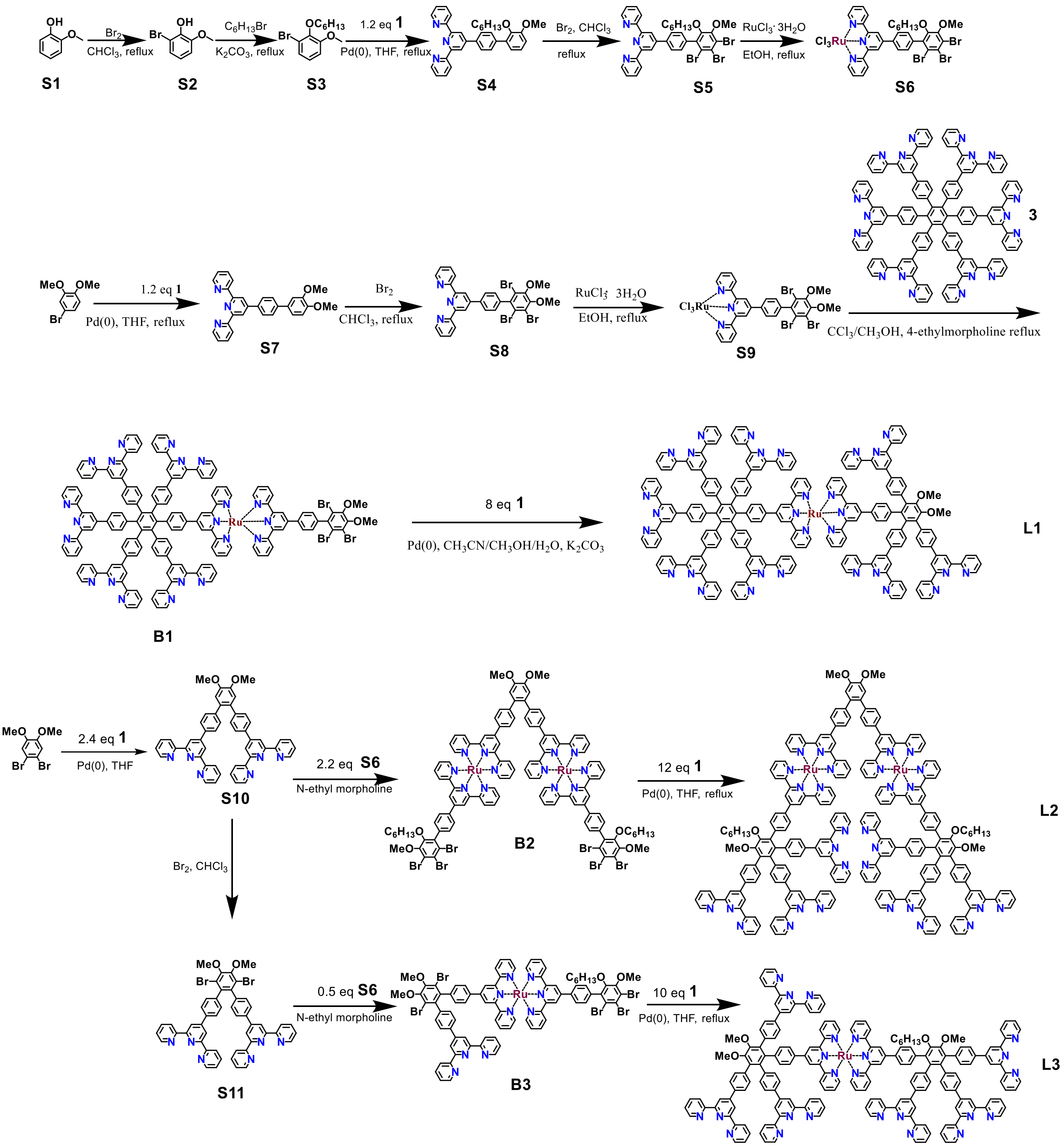

Scheme S1. The synthetic route of metallo-organic ligands module **L1-3**. Related to Figure 2.

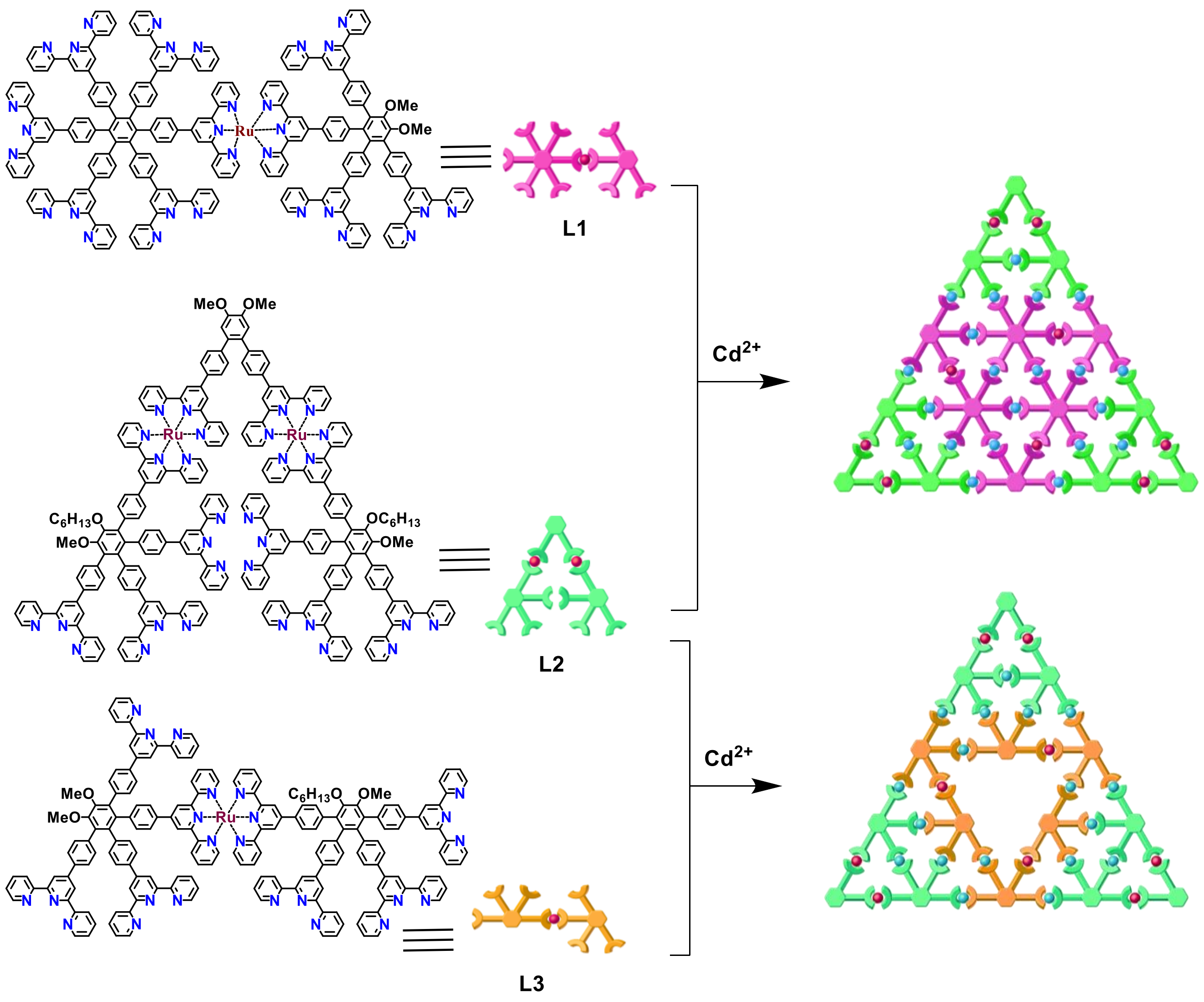

Scheme S2. The synthetic route of the third generation metallo-Pascal's Triangle G3 **PT** and the second generation metallo-Sierpiński Triangle G2 **ST**. Related to Figure 2.

Transparent Methods

2-Methoxyphenol and 1,2-dibromo-4,5-dimethoxybenzene were purchased from Aldrich and used without further purification. 4'-Boronatophenyl[2,2':6',2'']terpyridine **1,** tetra*kis*terpyridine **2** and star shaped ligand **3** were synthesized according to a reported method (Jarozs et al., 2009; Schultz et al., 2012; Wang et al., 2011). Column chromatography was conducted using basic Al_2O_3 (Sinopharm Chemical Reagents Co. Ltd, 200-300 mesh) or SiO_2 (Qingdao Haiyang Chemical Co., Ltd, 200-300 mesh) and the separated products were confirmed by ^1H NMR and ^{13}C NMR spectra using a Bruker Avance 400-MHz and 500-MHz NMR spectrometers in $CDCl_3$, DMSO-D_6, and CD_3CN with a TMS standard.

Electrospray ionization mass (ESI-MS). ESI-MS spectra were recorded with a Waters Synapt G2 tandem mass spectrometer, using solutions of 10 μg samples in 1 mL of $CHCl_3$/MeOH (1:3, v/v) for the ligands or 0.5 mg in 1 mL of MeCN/MeOH (3:1, v/v) for complexes. The ESI-MS experiments were performed under the following conditions: ESI capillary voltage, 3 kV; sample cone voltage, 30 V; extraction cone voltage, 3.5 V; source temperature 100 ºC; desolvation temperature, 100 ºC; cone gas flow, 10 L/h; desolvation gas flow, 700 L/h (N_2); source gas control, 0 mL/min; trap gas control, 2 mL/min; Helium cell gas control, 100 mL/min; ion mobility (IM) cell gas control, 30 mL/min; sample flow rate, 5 μL/min; IM traveling wave height, 25 V; and IM traveling wave velocity, 1000 m/s. Q was set in rf-only mode to transmit all ions produced by ESI into the triwave region for the acquisition of TWIM MS data.

The transmission electron microscope (TEM). TEM images were recorded with a JEOL 2010. The sample was prepared by drop-casting a sample MeCN solution (1×10^{-6} M) onto a carbon-coated Cu grid and extra solution was absorbed by filter paper to avoid aggregation, then dried *in vacuo* for 2 h, about 10 pictures were recorded for each architecture.

Atomic force microscopy (AFM). AFM was conducted on a Bruker Dimension Icon AFM system with ScanAsyst using non-contact/dynamic mode/tapping mode AFM probes, scan mode: contact mode; Scan rate, 2.00 Hz. The data were processed by NanoScope Analysis version 1.5 (Bruker Software, Inc.). The sample was prepared by drop-casting a sample MeCN solution (1×10^{-7} M) onto mica surface and the excess solvent was absorbed by filter paper to avoid aggregation, then dried *in vacuo* for 2 h, about 10 pictures were recorded for each architecture.

X-ray photoelectron spectroscopy (XPS). XPS data was obtained using an EscaLab Xi+ Versa Probe apparatus equipped with an Al Kα Xray source (1486.6 eV). Energy Step Size, 0.050 eV; Pass Energy 100.0 eV. All measurements were done at room temperature.

Molecular Modeling. All the structural optimization and energy calculation were carried out in Materials Studio version 6.1, using the Geometry Optimization and Energy tasks in the Forcite module (Accelrys Software, Inc.). The initial structural model was built up with all counterions omitted for clarity. The Geometry Optimization was performed by using Universal Force Field (UFF)[2] with atom-based summation and cubic spline truncation for both the electrostatic and Van der Waals parameters.

Synthesis of the Ligands and Complexes

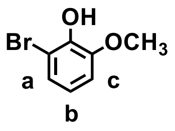

2-Bromo-6-methoxyphenol (**S2**). 2-Methoxyphenol **S1** (6.2 g, 50 mmol) was dissolved in dry THF (250 mL), then it was cooled to -50 °C with dry ice in an EtOH bath, then Br_2 (7.9 g, 50 mmol) was slowly dripped into the mixture maintaining this temperature for 2 h. The mixture was warmed to 25 °C with stirring for 4 hours; then the mixture was poured into an aqueous $NaHSO_3$ solution and extracted with CH_2Cl_2 (100 mL, ×3). The combined organic phase was washed sequentially with an aqueous $NaHCO_3$ solution, then brine. After drying over anh. Na_2SO_4, the solvent was removed *in vacuo* giving a residue, which was purified on a SiO_2 column (200-300 mesh), eluent with CH_2Cl_2: petroleum ether (1:3) to give **S2**, as a white solid: 5 g (65 %); ^{1}H NMR (400 MHz, $CDCl_3$): δ 7.12 (dd, *J* = 8.1, 1.4 Hz, PhH^a, 1H), 6.84 (dd, *J* = 8.1, 1.4 Hz, PhH^b, 1H), 6.77 (t, *J* = 8.1 Hz, PhH^c, 1H), 3.92 (s, OCH_3, 3H); ^{13}C NMR (101 MHz, $CDCl_3$): δ 147.36, 143.16, 124.83, 120.66, 109.92, 108.38, 56.33; ESI/MS (m/z): Calcd. $[M+H]^+$: 204.0, Found: 204.1.

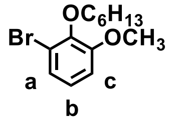

3-Bromo-2-hexyloxy-methoxybenzene (**S3**). A mixture of **S2** (2.03 g, 10 mmol), 1-bromohexane (1.64 g, 10 mmol), K_2CO_3 (1.48 g, 11 mmol), and DMF (100 mL) in a 250 mL 3-neck round bottom flask was stirred at 100 °C for 24 h under N_2, then cooled to 25 °C; the solvent was removed *in vacuo*. The residue was purified on a SiO_2 column eluting with petroleum ether (bp: 60-90 °C) to give the desired monomer, which was triturated with MeOH to generate (73 %) **S3**, as a white powder: 2.1 g; ^{1}H NMR (500 MHz, $CDCl_3$): δ 7.15 (dd, *J* = 8.0, 1.4 Hz, PhH^a, 1H), 6.93 (t, *J* = 8.1 Hz, PhH^b, 1H), 6.86 (dd, *J* = 8.2, 1.4 Hz, PhH^c, 1H), 4.00 (t, *J* = 6.7 Hz, OCH_2R, 2H), 3.87 (s, OCH_3, 3H), 1.83 (m, H^{Alkyl}, 2H), 1.51 (m, H^{Alkyl}, 6H), 1.37 (dd, *J* = 7.4, 3.6 Hz, H^{Alkyl}, 3H); ^{13}C NMR (126 MHz, $CDCl_3$): δ 154.04, 146.05, 124.86, 124.62, 118.06, 111.78, 73.39, 56.06, 31.68, 30.17, 25.65, 22.66, 14.08; ESI/MS (m/z): Calcd. $[M+H]^+$: 287.2, Found: 287.2.

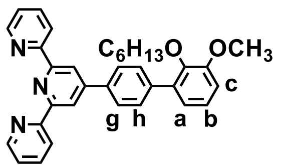

Monomer S4. To a solution of **S3** (287 mg, 1 mmol) and 4'-(4-boronatophenyl)[2, 2':6',2"]terpyridine (**1**; 391, 1.1 mmol) in THF (100 mL), aqueous Na_2CO_3 (2 mL, 1 M) was added. The mixture was freeze-pump-thawed (×3) and backfilled with Argon; then $Pd(PPh_3)_4$ (50 mg, 5% m/m) was added. After refluxing for 12 h under Argon, the mixture was cooled to 25 °C and poured into an aq. NH_4Cl solution. This aqueous layer was extracted with $CHCl_3$, then the combined organic phase was washed with brine and dried ($MgSO_4$). After concentration *in vacuo,* the residue was purified by flash column chromatography (Al_2O_3 200-300 mesh), eluting with $CHCl_3$:petroleum ether (2:1) to give **S4**, as a white solid: 320 mg (62%); ^{1}H NMR (500 MHz, $CDCl_3$): δ 8.84 (s, tpy$H^{3',5'}$, 2H), 8.80-8.73 (m, tpy$H^{6,6''}$, 2H), 8.71 (d, J = 7.9 Hz, tpy$H^{3,3''}$, 2H), 8.00 (d, J = 8.3 Hz, PhH^g, 2H), 7.91 (td, J = 7.8, 1.7 Hz, tpy$H^{4,4''}$, 2H), 7.75 (d, J = 8.3 Hz, PhH^h, 2H), 7.40–7.34 (m, tpy$H^{5,5''}$, 2H), 7.16 (t, J = 7.9 Hz, PhH^a, 1H), 7.05 (dd, J = 7.7, 1.3 Hz, PhH^b, 1H), 6.97 (dd, J = 8.1, 1.2 Hz, PhH^c ,1H), 3.94 (s, OCH_3, 3H), 3.73 (t, J = 6.6 Hz, OCH_2R, 2H), 1.61–1.51 (m, H^{Alkyl}, 2H), 1.32–1.11 (m, H^{Alkyl}, 6H), 0.82 (t, H^{Alkyl}, J = 7.2 Hz, 3H); ^{13}C NMR (126 MHz, $CDCl_3$): δ 156.35, 155.96, 153.39, 150.03, 149.14, 146.02, 139.35, 136.98, 136.81, 135.67, 130.06, 126.86, 123.95, 123.77, 122.49, 121.33, 118.78, 111.81, 73.50, 55.98, 31.54, 30.05, 25.58, 22.61, 14.01; ESI/MS (m/z): Calcd $[M+H]^+$: 516.4, Found: 516.2.

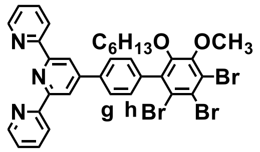

Monomer S5. Monomer **S4** (102 mg, 200 μmol) was dissolved in $CHCl_3$ (100 mL), Br_2 (1.58 g, 10 mmol) was slowly added under stirring, then the mixture was refluxed for 24 h with a tube to capture the HBr effluent. After the solution became colorless, the mixture was cooled to 25 °C and poured into cold aqueous $NaHSO_3$ solution, then extracted with CH_2Cl_2 (100 mL each, ×3). The organic phase was combined and washed with brine and purified on a SiO_2 column eluting with a 1:3 mixture of CH_2Cl_2 : petroleum ether, the desired **S5** was obtained, as a white solid: 100 mg (67%):.^{1}H NMR (400 MHz, $CDCl_3$): δ 8.83 (s, tpy$H^{3,3''}$,2H), 8.76 (ddd, J = 4.8, 1.7, 0.9 Hz, tpy$H^{6,6''}$ 2H), 8.72 (dt, J = 8.0, 1.0 Hz, tpy $H^{3,3''}$, 2H), 8.00 (d, PhH^g, 2H), 7.92 (m, tpy$H^{4,4''}$, 2H), 7.40 (ddd, J = 6.7, 3.8, 1.4 Hz, PhH^h, tpy$H^{5,5''}$, 4H), 3.96 (s, OCH_3, 3H), 3.76 (t, J = 6.5 Hz, OCH_2R, 2H), 1.12 (ddd, J = 11.0, 10.2, 4.5 Hz, H^{Alkyl}, 6H), 0.78 (t, J

= 7.0 Hz, H^{Alkyl}, 3H); ^{13}C NMR (126 MHz, $CDCl_3$): δ 156.24, 156.02, 151.52, 150.49, 149.90, 149.16, 138.78, 138.70, 138.21, 136.87, 130.28, 127.03, 123.86, 123.12, 121.99, 121.68, 121.35, 118.98, 74.29, 60.86, 31.41, 29.91, 25.34, 22.55, 13.93, 1.01; ESI/MS (m/z): Calcd $[M]^+$: 752.2, Found: 751.9.

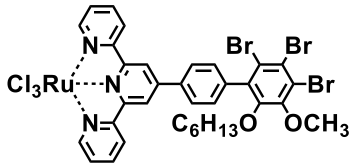

Monomer S6. A EtOH solution of **S5** (75 mg, 100 μmol) and $RuCl_3{\bullet}3H_2O$ (30 mg, 120 μmol) was refluxed for 12 h, then cooled to 25 °C and filtered to generate a brown powder (75 mg), which was washed (3X) with MeOH until the filtrate is clean and colorless; the solid was collected and dried *in vacuo* for 12 h and used directly for the next step without further purification: m. p. >320°C, *Anal*. Calcd. for: $C_{34}H_{30}Br_3Cl_3N_3O_2Ru$: C, 32.71; H, 2.42; N, 3.37. Found: C, 32.88; H, 2.36; N, 3.31.

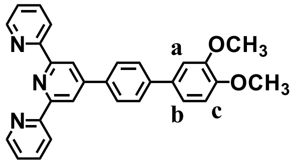

Monomer S7: To a solution of 4-Bromo-1,2-dimethoxybenzene (1 g, 2.25 mmol) and 4'-(4-boronatophenyl)[2, 2':6',2"]terpyridine (**1**; 1 g, 2.83 mmol) in THF (200 mL), aqueous Na_2CO_3 (7 mL, 1 M) was added. The mixture was freeze-pump-thawed and backfilled with Argon; then $Pd(PPh_3)_4$ (130 mg, 5% m/m) was added. After refluxing for 24 h under Argon, the mixture was cooled to 25 °C and poured into an aq. NH_4Cl solution. This aqueous layer was extracted with $CHCl_3$, then the combined organic phase was washed with brine and dried ($MgSO_4$). After concentration in vacuo, the residue was purified by flash column chromatography (Al_2O_3 200-300 mesh), eluting with $CHCl_3$:petroleum ether (2:1) to give **S7**, as a white solid: 1.1 g (70%). ^{1}H NMR (500 MHz, $CDCl_3$) δ 8.81(s, 2H, tpy-$H^{3',5'}$), 8.77-8.76(d, 2H, J=5 Hz tpy-$H^{6,6''}$), 8.72-8.70(d, 2H, J=10 Hz tpy-$H^{3,3''}$), 8.02-8.01(d, 2H, J=5 Hz, Ph-H^g), 7.93-7.90(t, 2H, J=5 Hz, tpy-$H^{4,4''}$), 7.74-7.72(d, 2H, J=5 Hz, Ph-H^h), 7.40-7.38(t, 2H, J=5 Hz, tpy-$H^{5,5''}$), 7.27-7.25(d, 1H, J=10 Hz, H^b), 7.21(s, 1H, H^a), 7.02-7.00(d, 1H, H^c), 4.02, 3.97. ^{13}C NMR (126 MHz, $CDCl_3$) δ 156.31, 155.98, 149.83, 149.29, 149.15, 148.97, 141.69, 136.89, 136.83, 133.41, 127.70, 127.27, 123.84, 121.41, 119.53, 118.65, 111.58, 110.39, 56.04. ESI/MS: Calculated $[M]^+$: 446.2, Found: 446.2.

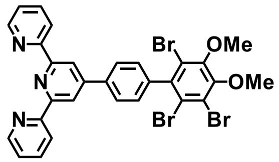

Monomer S8: The above **S7** (445 mg, 1 mmol) was dissolved in $CHCl_3$ (100 mL), then Br_2 (1.58 g, 10 mmol) was slowly added with stirring. After refluxing for 24 h, the mixture was cooled to 25 °C and poured into cold aq. $NaHSO_3$, then extracted with DCM (100 mL each, ×3). The organic extracts were combined and washed with a satd. NaCl solution. After in vacuo concentration, the residue was purified by flash column chromatography column (Al_2O_3, 200-300 mesh), with CH_2Cl_2: petroleum ether (1:3), as eluent, the desired monomer **S8** was obtained, as a white solid: 445 mg (65%). ^1H NMR (500 MHz, $CDCl_3$) δ 8.83(s, 2H, tpy-$H^{3',5'}$), 8.76-8.75(d, 2H, J=5 Hz tpy-$H^{6,6''}$), 8.73-8.71(d, 2H, J=10 Hz tpy-$H^{3,3''}$), 8.03-8.01((d, 2H, J=5 Hz, Ph-H^g), 7.93-7.90(t, 2H, J=5 Hz, tpy-$H^{4,4''}$), 7.40-7.38(t, 2H, J=5 Hz, tpy-$H^{5,5''}$), 7.34-7.32(d, 2H, J=5 Hz, Ph-H^h), 4.00, 3.98. ^{13}C NMR (126 MHz, $CDCl_3$) δ 156.19, 155.98, 151.57, 150.73, 149.90, 149.16, 142.72, 140.44, 138.44, 136.90, 129.72, 127.41, 123.88, 121.77, 121.39, 121.35, 119.48, 119.09, 60.98, 60.92. ESI/MS: Calculated $[M]^+$: 683.91, Found: 683.94.

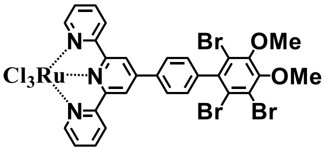

Monomer S9: A EtOH solution of **S8** (223 mg, 0.5 mmol) and $RuCl_3{\cdot}3H_2O$ (195 mg, 0.75 mmol) was refluxed for 12 h, then cooled to 25 °C and filtered to generate a brown powder (300 mg), which was washed (×3) with MeOH until the filtrate is clean and colorless; the solid was collected and dried in vacuo for 12 h and used directly for the next step without further purification.

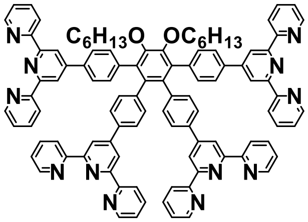

Monomer 3: 3 was synthesized according to the literature.[S2] ^1H NMR (500 MHz, $CDCl_3$) δ 8.74(s, 4H, tpyA $H^{3',5'}$), 8.70-8.69(d, J =5 Hz, 4H, tpyA $H^{6,6''}$), 8.65-8.63(d, J =10 Hz, 4H, tpyA $H^{3,3''}$), 8.59(s, 4H, tpyB $H^{3',5'}$, d, J =5 Hz, 4H, tpyB $H^{6,6''}$), 8.55-8.53(d, J =10 Hz, 4H, tpyB $H^{3,3''}$), 7.87-7.84(m, 4H, tpyA $H^{4,4''}$), 7.81-7.80(d, J =10 Hz, 4H, PhA H^g), 7.79-7.76 (m, 4H, tpyB $H^{4,4''}$), 7.57-7.55(d, J =10 Hz, 4H, PhB H^g), 7.39-7.37 (d, J =10 Hz, 4H, PhA H^h), 7.33-7.31(m, 4H, tpyA $H^{5,5''}$), 7.25-7.22 (m, 4H, tpyB $H^{5,5''}$), 7.06-7.05(d, J =10 Hz, 4H, PhB H^h), 3.88 (m, OCH_2, 4H), 1.79, 1.48, 1.15, 0.78(m, Halkyl, 22H). 13C NMR (101 MHz, $CDCl_3$) δ 156.39, 156.36, 155.78, 155.59, 150.08, 150.04, 149.61, 149.06, 148.94, 140.72, 138.28, 136.72, 136.57, 136.47, 136.14, 136.05, 135.21, 132.10, 131.72, 126.23, 126.00, 123.62,

123.45, 121.27, 121.17, 118.86, 118.69, 73.83, 31.62, 30.24, 25.63, 22.63, 14.01. ESI/MS (m/z): Calculated $[M+2H]^{2+}$: 753.9, Found: 753.9.

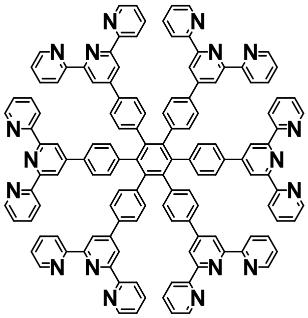

Monomer 3: 3 was synthesized according to the literature.[S3] ^{1}H NMR (400 MHz, $CDCl_3$) δ 8.59 (s, tpy- $H^{3',5'}$,12H), 8.57 (d, tpy-$H^{6,6''}$, 12H), 8.52 (d, J = 7.9 Hz, tpy-$H^{3,3''}$, 12H), 7.76 (td, J = 7.8, 1.6 Hz, Ph-H^g, 12H), 7.59 (d, J = 8.2 Hz, ph-H^h ,12H), 7.22 (dd, J = 6.8, 5.4 Hz, tpy-$H^{4,4''}$,12H), 7.16 (t, J = 8.2 Hz, tpy-$H^{5,5''}$, 12H).^{13}C NMR (101 MHz, $CDCl_3$) δ 167.11, 156.37, 155.60, 149.60, 148.94, 141.27, 140.29, 136.57, 131.90, 126.02, 123.44, 121.17, 118.75. ESI/MS (m/z): Calculated $[M+H]^+$: 1923.2, Found: 1923.2.

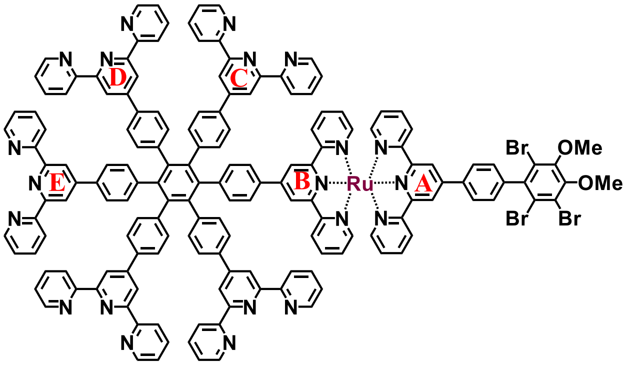

Complex B1: To a suspension of **3** (200 mg, 104 μmol) and **S9** (89 mg, 100 μmol) in $CHCl_3$/MeOH [200 mL, 1/1(v/v)], *N*-ethylmorpholine (5 drops), as a reductant, was added. The mixture was refluxed for 24 h giving a dark red solution, which was cooled to 25 °C and then the solvent was removed *in vacuo* giving a residue, which was extracted with $CHCl_3$ (3X). The combined organic phase was washed with brine and dried over anh. $MgSO_4$. After concentrating *in vacuo*, the residue was purified by flash column chromatography (Al_2O_3, 200-300 mesh) eluting with $CHCl_3$/MeOH (20/1) to give (70%) **6**, as a red powder. ^{1}H NMR (500 MHz, CDCl3) δ 9.09(s, 2H, A-tpy-$H^{3',5'}$), 8.84(s, 2H, B-tpy-$H^{3',5'}$), 8.74-8.73(d, 2H, A-tpy-$H^{3',3''}$), 8.60-8.57(m, 20H, C-tpy-$H^{3',5'}$, D-tpy-$H^{3',5'}$, E-tpy-$H^{3',5'}$, C-tpy-$H^{6,6''}$, D-tpy-$H^{6,6''}$, E-tpy-$H^{6,6''}$, B-tpy-$H^{3,3''}$), 8.51-8.48(m, 10H, C-tpy-$H^{3,3''}$, D-tpy-$H^{3,3''}$, E-tpy-$H^{3,3''}$), 8.31-8.29(d, 2H, J=10Hz, A-PH-

H^{g}), 7.95, 7.92(m, 4H, C-tpy-$H^{4,4''}$), 7.89-7.87(m, 2H, A-tpy-$H^{4,4''}$), 7.86-7.77(m, 10H, B-tpy-$H^{4,4''}$, D-tpy-$H^{4,4''}$, E-tpy-$H^{4,4''}$, B-PH-H^{g}), 7.67-7.66(d, 4H, J=5 Hz, C-PH-H^{g}), 7.59-7.57(d, 6H, J=5 Hz, D-PH-H^{g}, E-PH-H^{g}), 7.46-7.45(d, 2H, J= 5 Hz, A-PH-H^{h}), 7.39-7.37(m, 8H, B-PH-H^{h}, C-PH-H^{h}, E-PH-H^{h}), 7.33-7.26(m, 10H, A-tpy-$H^{6,6''}$, B-tpy-$H^{6,6''}$, E-tpy-$H^{5,5''}$ D-PH-H^{h}), 7.21-7.20(m, 8H, C-tpy-$H^{5,5''}$, D-tpy-$H^{5,5''}$), 7.14(m, 2H, B-tpy-$H^{5,5''}$), 7.01(m, 2H, A-tpy-$H^{5,5''}$), 3.97, 3.94. ^{13}C NMR (126 MHz, $CDCl_3$) δ 157.87, 157.55, 155.06, 154.98, 154.80, 154.74, 152.71, 152.03, 151.74, 151.35, 150.78, 149.85, 148.67, 148.20, 147.76, 145.87, 144.25, 141.71, 141.26, 140.37, 140.07, 138.88, 138.57, 138.21, 138.02, 135.99, 135.02, 132.75, 132.11, 131.95, 130.55, 128.01, 127.77, 126.06, 126.01, 124.52, 124.18, 122.36, 121.88, 119.17, 119.02. ESI/MS (m/z): Calculated $[M]^{2+}$: 1352.76, Found: 1352.78.

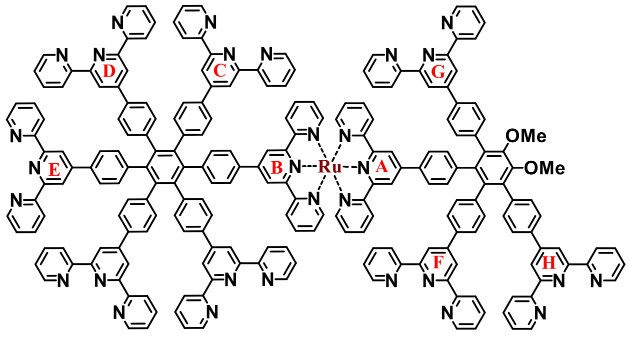

Ligand L1: To a solution of **B1** (60 mg, 16 μmol) and 4'-(4-boronatophenyl)[2,2':6',2'']terpyridine (**1**; 135 mg, 384 μmol) in DMSO (20 mL), aq. Na_2CO_3 (1 mL, 1 M) was added. The mixture was freeze-pump-thawed three (3X) and backfilled with Argon; then $Pd(PPh_3)_4$ (35 mg) was added. After refluxing for 72 h under Argon, the mixture was cooled to 25 °C and then poured into aq. NH_4Cl. The aqueous layer was extracted with $CHCl_3$, and then the combined organic phase was washed with brine and dried over anh. $MgSO_4$. After concentration *in vacuo*, the residue was purified by flash column chromatography (Al_2O_3, 200-300 mesh), eluting with $CHCl_3$:MeOH (40:1) to give (50 %) **L1**. ^{1}H NMR (400 MHz, $CDCl_3$) δ 8.75-8.74(m, 4H, A-tpy-$H^{3',5'}$, B-tpy-$H^{3',5'}$), 8.63(s, 2H, H-tpy-$H^{3',5'}$), 8.60-8.54(m, 12H, C-tpy-$H^{3',5'}$, D-tpy-$H^{3',5'}$, E-tpy-$H^{3',5'}$, F-tpy-$H^{3',5'}$, G-tpy-$H^{3',5'}$), 8.48-8.41(m, 38H, F-tpy-$H^{3',5'}$, C-tpy-$H^{6,6''}$, D-tpy-$H^{6,6''}$, E-tpy-$H^{6,6''}$, F-tpy-$H^{6,6''}$, G-tpy-$H^{6,6''}$, H-tpy-$H^{6,6''}$, A-tpy-$H^{3,3''}$, B-tpy-$H^{3,3''}$, C-tpy-$H^{3,3''}$, D-tpy-$H^{3,3''}$, E-tpy-$H^{3,3''}$, F-tpy-$H^{3,3''}$, G-tpy-$H^{3,3''}$, H-tpy-$H^{3,3''}$), 7.85-7.80(m, 6H, A-PH-H^{g}, B-PH-H^{g}, H-PH-H^{g}), 7.79-7.65(m, 32H, A-tpy-$H^{4,4''}$, B-tpy-$H^{4,4''}$, D-tpy-$H^{4,4''}$, C-tpy-$H^{4,4''}$, E-tpy-$H^{4,4''}$, F-tpy-$H^{4,4''}$, G-tpy-$H^{4,4''}$, G-PH-H^{g}, E-PH-H^{g}, F-PH-H^{g}), 7.59-7.54(m, 14H, C-PH-H^{g}, D-PH-H^{g}, H-tpy-$H^{4,4''}$, A-PH-H^{h}, B-PH-H^{h}), 7.42-7.40(m, 2H, H-PH-H^{h},), 7.36-7.26(m, 14H, C-PH-H^{h}, D-PH-H^{h}, E-PH-H^{h}, F-PH-H^{h}, G-PH-H^{h}), 7.25-7.19(m, 10H, C-tpy-$H^{5,5''}$, D-tpy-$H^{5,5''}$, F-tpy-$H^{5,5''}$), 7.16-7.15(m, 6H, F-tpy-$H^{5,5''}$, G-tpy-$H^{5,5''}$, H-tpy-$H^{5,5''}$), 7.10-7.07(m,

4H, A-tpy-$H^{6,6''}$, B-tpy-$H^{6,6''}$), 6.93-6.92(m, 4H, A-tpy-$H^{5,5''}$, B-tpy-$H^{5,5''}$), 3.75, 3.74. ESI/MS: Calculated $[M]^{2+}$: 1695.56, Found: 1695.54.

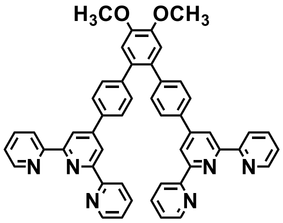

"V" Monomer S10. To a solution of 1,2-dibromo-4,5-dimethoxybenzene (1.48 g, 5 mmol) and 4'-(4-boronatophenyl)[2,2':6',2'']terpyridine (**1**; 3.54 g, 10.00 mmol) in THF (200 mL), aq. Na_2CO_3 (20 mL, 2 M) was added. The mixture was freeze-pump-thawed (3X) and backfilled with Argon; then $Pd(PPh_3)_4$ (100 mg) was added. After refluxing for 12 h under Argon, the mixture was cooled to 25 °C and poured into an aq. NH_4Cl solution. The aqueous layer was extracted with $CHCl_3$, and then the combined organic phase was washed with brine and dried with anh. $MgSO_4$. After concentration *in vacuo*, the residue was purified by flash column chromatography (Al_2O_3 200-300 mesh), eluting with $CHCl_3$: petroleum ether (2:1) to give the desired "**V**" ligand, as a white solid: 2.4 g (64%); ^1H NMR (400 MHz, $CDCl_3$): δ 8.76 (s, 4H), 8.70 (ddd, *J* = 4.8, 1.7, 0.8 Hz, 4H), 8.66 (d, *J* = 7.9 Hz, 4H), 7.86 (ddd, *J* = 8.3, 6.6, 2.8 Hz, 8H), 7.34 (m, 8H), 7.28 (s, 2H), 4.03 (s, 6H); ^{13}C NMR (101 MHz, $CDCl_3$) δ 156.31, 155.88, 149.77, 149.09, 148.54, 142.22, 136.82, 136.37, 132.42, 130.51, 127.05, 123.74, 121.34, 118.79, 113.70, 113.70, 56.19.

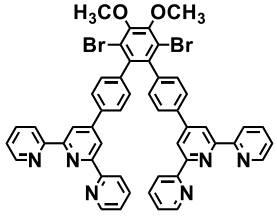

"V" Monomer S11. The above **S10** (102 mg, 200 μmol) was dissolved in $CHCl_3$ (100 mL), then Br_2 (1.58 g, 10 mmol) was slowly added with stirring. After refluxing for 24 h, the mixture was cooled to 25 °C and poured into cold aq. $NaHSO_3$, then extracted with DCM (100 mL each, X3). The organic extracts were combined and washed with a satd. NaCl solution. After *in vacuo* concentration, the residue was purified by flash column chromatography column (Al_2O_3, 200-300 mesh), with CH_2Cl_2 : petroleum ether (1:3), as a eluent, the desired monomer **S11** was obtained, as a white solid: 100 mg (67%), m. p. = 301 °C; ^1H NMR (500 MHz, $CDCl_3$): δ 8.73–8.64 (m, 8H), 8.63 (d, *J* = 7.9 Hz, 4H), 7.84 (t, *J* = 7.7 Hz, 4H), 7.79 (d, *J* = 8.1 Hz, 4H), 7.34–7.29 (m, 4H), 7.21 (d, *J* = 8.0 Hz, 4H), 4.06 (s, 6H); ^{13}C NMR (126

MHz, $CDCl_3$): δ 156.29, 155.83, 150.70, 149.69, 149.06, 140.57, 139.09, 137.20, 136.73, 130.67, 126.75, 123.67, 121.27, 119.50, 118.93, 60.91; 2D-COSY spectrum: ESI-MS: $[M + H]^+$ = 909.11 (found = 909.1).

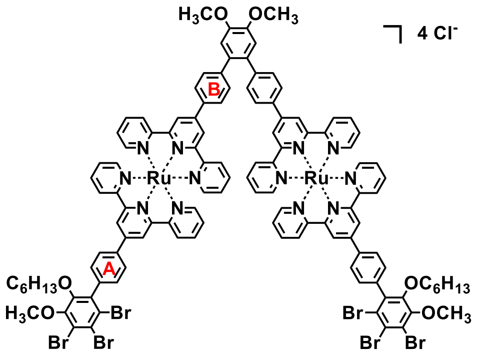

Complex B2. To a mixture of **S10** (37.2 mg, 50 μmol) and **S6** (105.6 mg, 110 μmol) in $CHCl_3$/MeOH (200 mL), a few drops of *N*-ethylmorpholine was added, as a catalyst. The mixture was refluxed for 24 h, then cooled to 25 °C and the solvent was removed *in vacuo* leaving a residue that was purified on an Al_2O_3 column with CH_2Cl_2/MeOH (40:1), as a eluent, then drying *in vacuo* to give (50%) pure complex **B2**, as a red powder: 62 mg: ^{1}H NMR (400 MHz, MeOD): δ 9.38 (s, tpy^B$H^{3',5'}$, 4H), 9.35 (s, tpy^A$H^{3',5,'}$ 4H), 8.94 (dd, J = 8.0, 4.5 Hz, tpy^A$H^{3,3''}$, tpy^B$H^{3,3''}$, 8H), 8.44 (d, J = 8.4 Hz, Ph^BH^g, 4H), 8.32 (d, J = 8.4 Hz, Ph^AH^g, 4H), 8.03 (ddd, J = 5.7, 4.8, 2.4 Hz, tpy^A$H^{4,4''}$, tpy^B$H^{4,4''}$, 8H), 7.71 (d, J = 8.3 Hz, Ph^BH^h, 4H), 7.66 (d, J = 8.3 Hz, Ph^AH^h, 4H), 7.58 (t, J = 6.2 Hz, tpy^A$H^{6,6''}$, tpy^B$H^{6,6''}$, 8H), 7.30 (m, tpy^A$H^{5,5''}$, tpy^B$H^{5,5''}$, 8H), 7.25 (s, PhH^J, 2H), 4.06 (s, OCH_3 , 6H), 3.98 (s, OCH_3,6H), 3.90 (t, J = 6.3 Hz, OCH_2R, 4H), 1.47 (d, J = 6.7 Hz, H^{alkyl}, 4H), 1.21 (m, H^{alkyl}, 12H), 0.82 (t, J = 7.0 Hz, H^{alkyl}, 6H); ^{13}C NMR (101 MHz, $CDCl_3$): δ 162.34, 162.27, 159.60, 159.49, 155.91, 155.79, 154.42, 153.24, 152.26, 152.07, 147.93, 144.16, 142.41, 142.05, 142.02, 142.00, 140.12, 138.63, 136.21, 135.06, 134.90, 131.60, 131.48, 131.35, 131.17, 128.67, 128.53, 126.72, 125.58, 125.22, 125.14, 125.05, 124.90, 77.54, 63.70, 35.03, 33.85, 33.39, 33.19, 30.55, 28.66, 26.27, 17.00; ESI/MS (m/z): Calcd $[M]^{4+}$: 613.52, Found: 613.64; $[M+Cl^-]^{3+}$: 829.68; Found: 829.78.

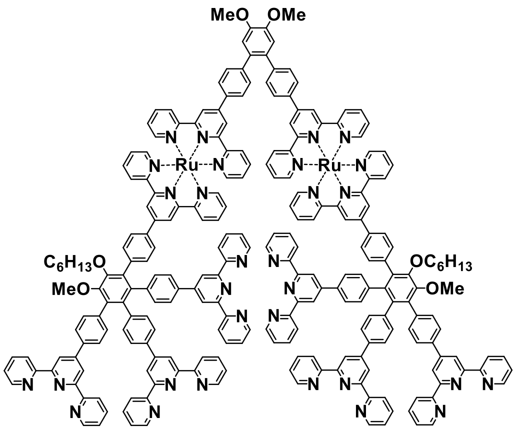

Ligand L2. To a solution of **B2** (25.85 mg, 10 μmol) and 4'-(4-boronato-phenyl)[2,2':6',2"]terpyridine (**1**; 70.8 mg, 200 μmol) in DMSO (20 mL), aqueous Na_2CO_3 (0.5 mL, 1 M) was added. The mixture was freeze-pump-thawed (3X) and backfilled with Argon; then $Pd(PPh_3)_4$ (10 mg) was added. After refluxing for 96 h under Argon, the mixture was cooled to 25 °C and then poured into aq. NH_4Cl. The aqueous layer was extracted with $CHCl_3$, and the combined organic phase was washed with brine and dried over anh $MgSO_4$. After concentration *in vacuo*, the residue was flash column chromatographed (Al_2O_3, 200-300 mesh) eluting with $CHCl_3$:MeOH (30:1) to give (62%) **L2**, as a red solid: 25 mg; ^{1}H NMR (500 MHz, $CDCl_3$,): δ 9.41 (s, tpy^B$H^{3',5'}$, 4H), 9.23 (d, J = 7.7 Hz, tpy^B$H^{3,3''}$, 4H), 9.02 (s, tpy^B$H^{3',5'}$, 4H), 8.77 (d, J = 8.7 Hz, tpy^A$H^{3,3''}$, 4H), 8.73 -8.69 (m, tpy^C$H^{3',5'}$, tpy^D$H^{3',5'}$, tpy^E$H^{3',5'}$, 12H), 8.64 (d, J = 7.7 Hz, tpy^C$H^{6,6''}$, tpy^D$H^{6,6''}$, tpy^E$H^{6,6''}$, 12H), 8.54 (ddd, J = 24.2, 15.2, 8.1 Hz, tpy^C$H^{3,3''}$, tpy^D$H^{3,3''}$, tpy^E$H^{3,3''}$, 12H), 8.32 (d, J = 7.6 Hz, Ph^BH^g, 4H), 8.18 (d, J = 7.6 Hz, Ph^AH^g, 4H), 7.86 (m, Ph^CH^g, Ph^DH^g, Ph^EH^g, 12H), 7.79 (m, tpy^B$H^{4,4''}$, tpy^A$H^{4,4''}$, 8H), 7.58 (dd, J = 20.8, 8.6 Hz, Ph^BH^h, Ph^AH^h, 8H), 7.38 (m, tpy^B$H^{6,6''}$, tpy^A$H^{6,6''}$, 8H), 7.24 (m, Ph^CH^h, Ph^DH^h, Ph^EH^h, 12H), 7.10 (m, tpy^B$H^{5,5''}$, tpy^A$H^{5,5''}$, tpy^C$H^{5,5''}$, tpy^D$H^{5,5''}$, tpy^E$H^{5,5''}$, 20H), 6.94 (d, J = 8.5 Hz, phH^J, 2H), 4.05 (s, OCH_3, 6H), 3.99 (s, OCH_3, 6H), 3.75 (d, OCH_2R, 4H), 1.43 (m, H^{alkyl}, 6H), 1.26 (d, J = 12.6 Hz, H^{alkyl}, 8H), 0.90 (d, J = 7.0 Hz, H^{alkyl}, 4H), 0.83 (t, J = 6.8 Hz, H^{alkyl}, 6H); ^{13}C NMR (126 MHz, $CDCl_3$): δ 158.18, 157.59, 156.34, 156.27, 155.81, 155.66, 155.61, 155.02, 149.05, 148.91, 138.24, 136.66, 132.01, 131.35, 127.96, 126.49, 126.04, 123.93, 123.67, 123.54, 121.31, 121.21, 118.93, 118.64, 118.37, 117.78, 116.39, 116.00, 60.84, 56.34, 31.91, 31.62, 31.59, 30.28, 29.68, 29.34, 25.64, 22.72, 14.14, 14.08; ESI/MS (m/z): Calcd $[M]^{4+}$: 957.5, Found: 957.5; $[M+Cl]^{3+}$:1288.6, Found:1288.6.

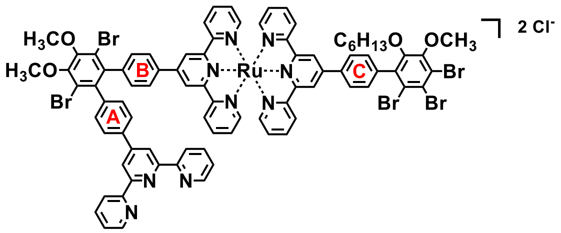

Complex B3. To a suspension of **S11** (48 mg, 50 μmol) and **S6** (91.1 mg, 100 μmol) in $CHCl_3$/MeOH (100 mL, 1:1(v/v)), 5 drops of *N*-ethylmorpholine, as a reductant, were added. The mixture was refluxed for 12 h becoming dark red over time. The mixture was cooled to 25 °C and then concentrated *in vacuo* to give a residue that was extracted (3X) with $CHCl_3$, and then the combined organic phase was washed with brine and dried with anh $MgSO_4$. After concentrated *in vacuo*, the residue was flash column chromatographed (Al_2O_3, 200-300 mesh) eluting with $CHCl_3$:MeOH (20:1) to give (30 %) **B3**, as a red powder: 35 mg, m.p. > 320 °C; ^{1}H NMR (500 MHz, $CDCl_3$): δ 9.39 (s, 2H), 9.17 (s, 2H), 9.13 (d, *J* = 8.1 Hz, 2H), 8.94 (d, *J* = 8.1 Hz, 2H), 8.69 (s, 2H), 8.64 (dd, *J* = 9.6, 6.7 Hz, 3H), 8.58 (d, *J* = 7.8 Hz, 2H), 8.27 (d, *J* = 7.7 Hz, 2H), 8.01 – 7.83 (m, 6H), 7.80 (d, *J* = 7.8 Hz, 2H), 7.58 (d, *J* = 7.8 Hz, 2H), 7.46 – 7.37 (m, 4H), 7.34 (t, *J* = 6.2 Hz, 4H), 7.29 – 7.20 (m, 6H), 7.22 – 7.10 (m, 2H), 4.08 (d, *J* = 4.9 Hz, 6H), 3.96 (s, 3H), 3.80 (t, *J* = 6.4 Hz, 2H), 0.90 (t, *J* = 6.4 Hz, 2H), 0.80 (t, *J* = 6.8 Hz, 3H); ^{13}C NMR (126 MHz, $CDCl_3$): δ 157.99, 155.90, 155.07, 152.62–151.50, 150.91, 150.51, 149.81, 149.33–149.00, 148.80, 148.50, 142.42, 140.77, 140.39, 139.28, 138.58, 137.24, 136.90, 135.54, 134.73, 131.68, 131.44–131.27, 131.03, 128.00, 127.70, 126.76, 125.83, 125.52, 124.05, 123.19, 122.16, 121.75, 121.55, 119.55, 119.26, 118.82, 114.06, 60.96, 33.81, 31.92, 31.43, 30.20, 29.92, 29.52, 29.34–29.27, 29.15, 28.95, 27.21, 25.37, 22.66, 14.09; ESI/MS m/z: Calcd $[M]^{2+}$: 882.5004, Found: 882.4975, Calcd $[M+Cl^-]^-$: 1797.9605, Found: 1797.9694.

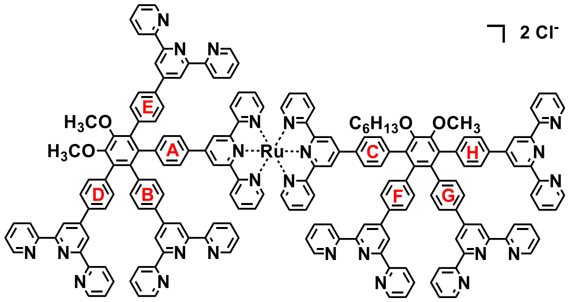

Ligand L3. To a solution of **B3** (32 mg, 20 μmol) and 4'-(4-boronatophenyl)[2,2':6',2"]terpyridine (**1**; 70.8 mg, 200 μmol) in DMSO (20 mL), aqueous Na_2CO_3 (0.5 mL, 1 M) was added. The mixture was freeze-pump-thawed (3X) and backfilled with Argon; then $Pd(PPh_3)_4$ (5 mg) was added. After refluxing

for 96 h under Argon, the mixture was cooled to 25 °C and poured into an aq. NH_4Cl solution. The aqueous layer was extracted with $CHCl_3$, and then the combined organic extract was washed with brine and dried over anh $MgSO_4$. After concentration *in vacuo,* the residue was flash column chromatographed (Al_2O_3, 200-300 mesh) eluting with $CHCl_3$: MeOH (40:1) to give (42%) **L3**, as a red solid: 20 mg, m. p. >320°C; ^{1}H NMR (400 MHz, $CDCl_3$): δ 9.06 (s, 2H), 8.87 (s, 2H), 8.78 (d, *J* = 8.0 Hz, 3H), 8.73 (d, *J* = 2.3 Hz, 4H), 8.71 – 8.61 (m, 17H), 8.60 – 8.43 (m, 20H), 8.21 (d, *J* = 8.4 Hz, 2H), 7.92 (d, *J* = 8.4 Hz, 3H), 7.89 – 7.69 (m, 24H), 7.61 – 7.56 (m, 5H), 7.53 (t, *J* = 6.1 Hz, 4H), 7.47 (d, *J* = 8.2 Hz, 3H), 7.45 – 7.39 (m, 6H), 7.39 – 7.29 (m, 11H), 7.26 – 7.19 (m, 8H), 7.12 (dt, *J* = 16.8, 5.4 Hz, 11H), 7.01 (dd, *J* = 10.8, 3.8 Hz, 3H), 6.89 (d, *J* = 8.7 Hz, 2H), 3.97 (t, *J* = 6.5 Hz, 2H), 3.81 (d, *J* = 3.2 Hz, 6H), 3.75 (s, 3H), 0.89 (q, *J* = 6.8 Hz, 6H), 0.82 (t, *J* = 6.8 Hz, 4H); ^{13}C NMR (126 MHz, $CDCl_3$): δ 156.30, 155.80, 148.99, 136.88–136.55, 131.36–128.79, 126.65–126.49, 123.71, 121.35, 118.73, 118.42, 77.26, 77.01, 76.76, 60.85, 50.84, 31.76, 30.82, 29.69, 29.35, 25.71–25.48, 22.68, 14.10, 1.01; ESI/MS (m/z): Calculated $[M]^{2+}$: 1453.16, Found: 1452.95

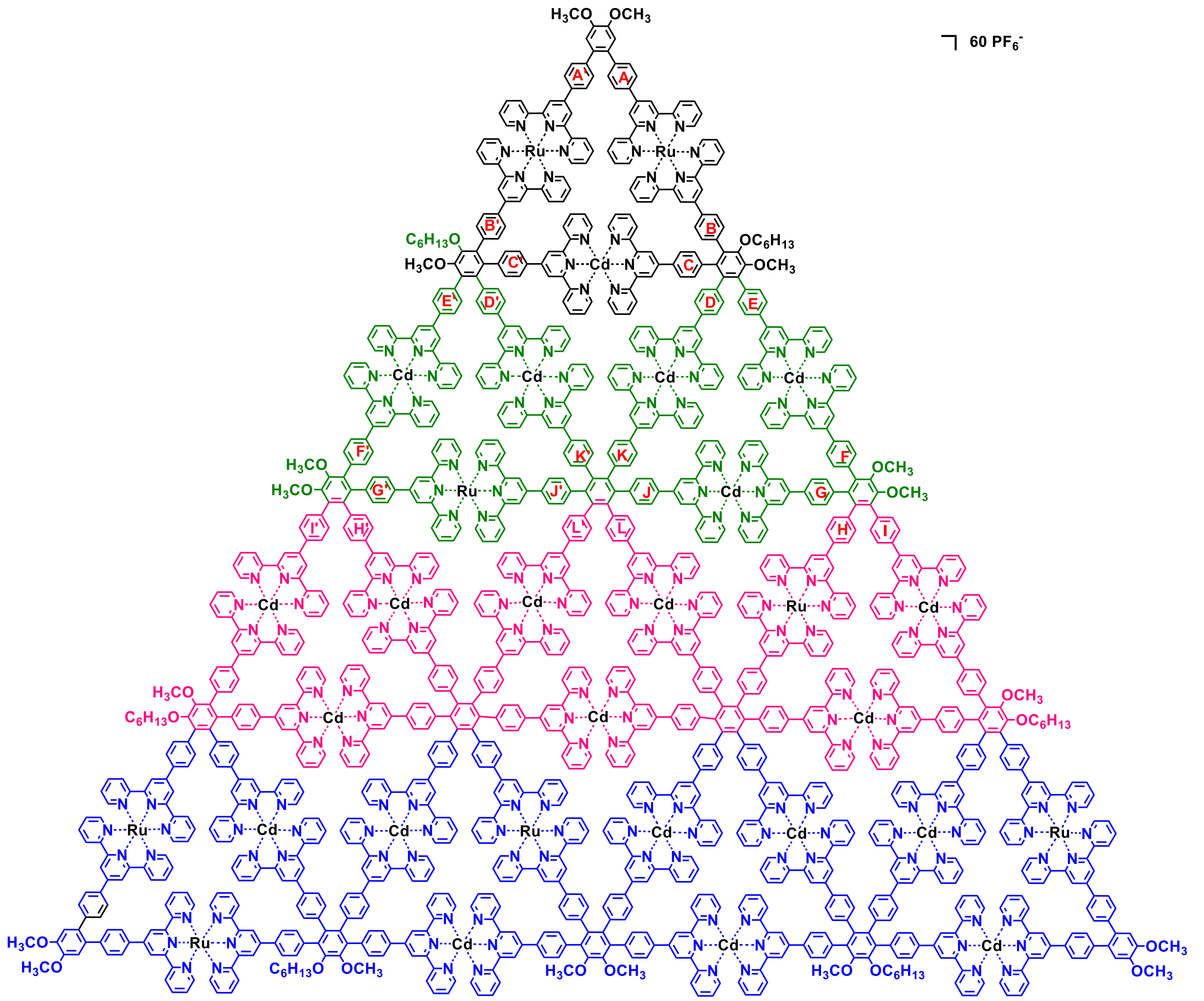

G3 Pascal Triangle (G3 **PT)**. To a solution of **L2**(5.00 mg, 1 μmol) and L1 (3.95 mg, 1 μmol) in $CHCl_3$/MeOH (10 mL, 1:1 v/v), a solution of $Cd(NO_3)_2$•$6H_2O$ (2.31 mg, 7.50 μmol) in MeOH (5 mL) was added dropwise, then the mixture was stirred at 25 °C for 8h. NH_4PF_6 (200 mg) was added resulting in the formation of an orange precipitate, which was filtered, then washed with water and MeOH to give (95%) the Pascal triangle (G3 **PT**): 8.5 mg; ^{1}H NMR (500 MHz, CD_3CN) δ 9.05 (m, 42H), 8.94 (m, 34H), 8.71-8.49 (m, 164H), 8.11 (m, 120H), 7.93-7.67 (m, 199H), 7.62–7.58 (m, 119H), 7.45 (m, 94H), 7.36 – 7.33-7.29 (m, 72H), 7.18-7.12(m, 73H), 7.07 – 6.95 (m, 48H), 4.15 – 4.12 (m, 12H), 4.07 (m, 18H), 4.00 (m, 10H), 3.93 (m, 24H), 1.30 (m, 76H), 0.91 (m, 24H); ESI-MS (m/z): 1898.20 [M-16 PF_6^-]$^{16+}$ (calcd *m/z*: 1900.18), 1780.04 [M-17 PF_6^-]$^{17+}$ (calcd m/z: 1779.88), 1673.49 [M-18PF_6^-]$^{18+}$ (calcd m/z: 1672.94), 1577.27 [M-19 PF_6^-]$^{19+}$ (calcd m/z: 1577.26), 1491.28 [M-20 PF_6^-]$^{20+}$ (calcd m/z: 1491.15), 1413.47 [M-

21 PF_6^-]$^{21+}$ (calcd m/z: 1413.24), 1342.58 [M-22 PF_6^-]$^{22+}$ (calcd m/z: 1342.41), 1277.56 [M-23 PF_6^-]$^{23+}$ (calcd m/z: 1277.74), 1218.59 [M-24 PF_6^-]$^{24+}$ (calcd. m/z: 1218.46), 1164.32 [M-25 PF_6^-]$^{25+}$(calcd *m/z*: 1163.93), 1113.54 [M-26 PF_6^-]$^{26+}$(calcd *m/z*: 1113.59).

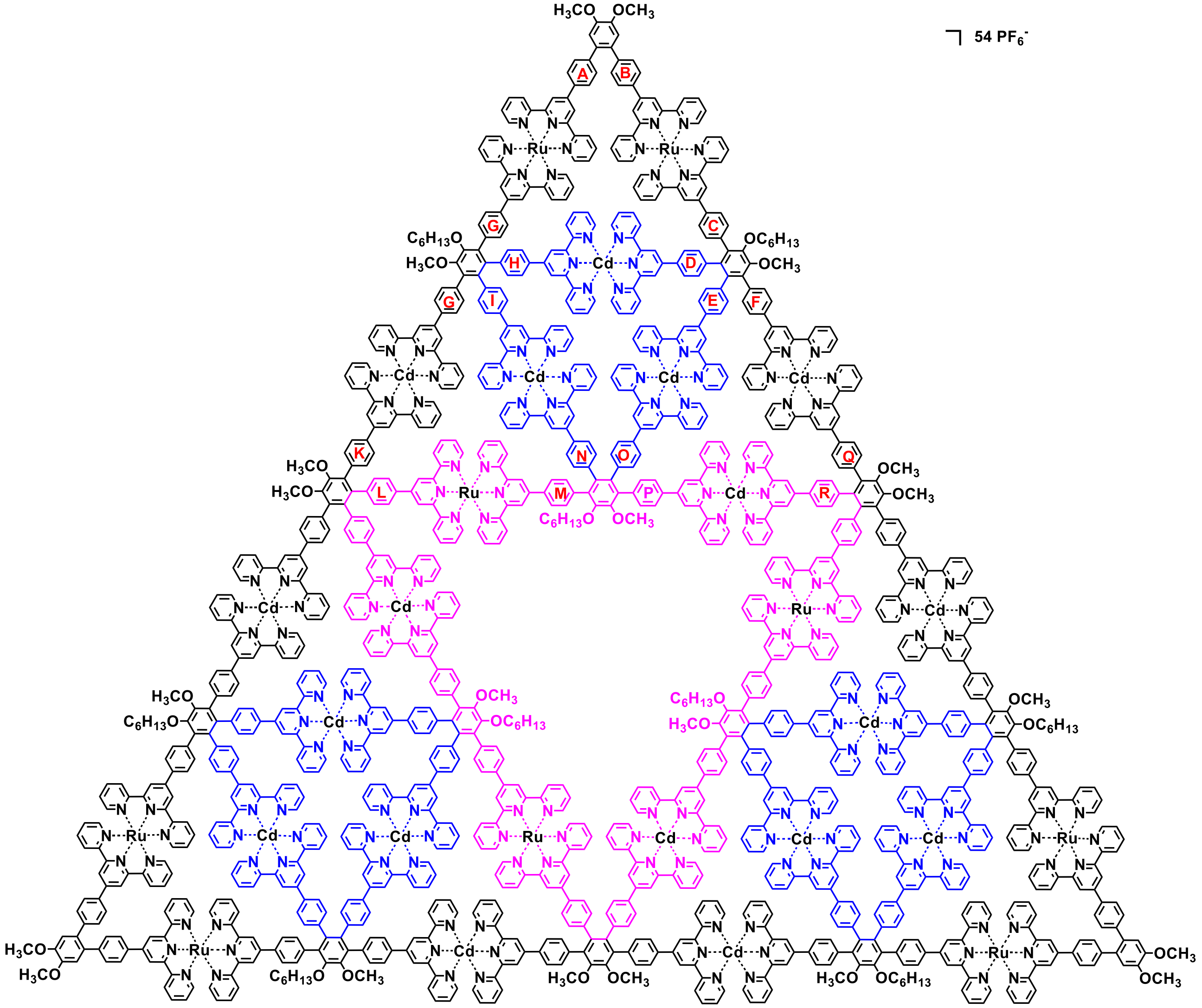

G2 Sierpinski Triangle (G2 **ST**). To a solution of ligand **L2** (3.956 mg, 1 μmol) and **L3** (3.01 mg, 1 μmol) in $CHCl_3$/MeOH (10 mL, 1:1 v/v), a solution of $Cd(NO_3)_2 \cdot 6H_2O$ (1.848 mg, 6 μmol) in MeOH (5 mL) was added dropwise; then the mixture was stirred at 60 °C for 8h. NH_4PF_6 (200 mg) was added to give an orange precipitate, which was filtrated, washed with water and MeOH to afford the desired triangle G2 **ST**, as an orange solid: 8.2 mg (92%); ^{1}H NMR (500 MHz, CD_3CN): δ 9.05 (m, 24H), 9.01 (m, 24H), 8.98 – 8.94 (m, 16H), 8.97 – 8.92 (m, 18H), 8.89 (m, 16H), 8.82 – 8.58 (m, 140H), 8.14 (m, 106H), 7.88 (m, 170H), 7.70 (m, 84H), 7.42 (m, 100H), 7.29 (m, 46H), 7.17 (m, 28H), 7.12 – 7.05 (m, 26H), 4.14 – 4.11 (m, 12H), 4.06 (m, 18H), 3.92 (m, 21H), 3.89 (m, 9H), 1.30 (m, 71H), 0.90 (m, 48H); ESI-MS (m/z):

2002.20 [M-14 PF_6^-]$^{14+}$ (calcd. m/z: 2002.33), 1859.26 [M-15 PF_6^-]$^{15+}$(calcd *m/z*:1859.18), 1734.22 [M-16 PF_6^-]$^{16+}$ (calcd *m/z*: 1733.93), 1623.59 [M-17 PF_6^-]$^{17+}$ (calcd m/z: 1623.40), 1525.42 [M-18PF_6^-]$^{18+}$ (calcd m/z: 1525.16), 1437.47 [M-19 PF_6^-]$^{19+}$ (calcd m/z: 1437.29), 1358.27 [M-20 PF_6^-]$^{20+}$ (calcd m/z: 1358.15), 1286.74 [M-21 PF_6^-]$^{21+}$ (calcd m/z: 1286.57), 1220.99 [M-22 PF_6^-]$^{22+}$ (calcd m/z: 1221.50), 1161.72 [M-23 PF_6^-]$^{23+}$ (calcd m/z: 1162.09

References

Jarozs, P.; Lotito, K.; Schneider, J.; Kumaresan, D.; Schmehl, R.; Eisenberg, R. *Inorg. Chem*. **2009**, *48*. 2420.

Schultz, A.; Li, X.; Barkakaty, B.; Moorefield, C. N.; Wesdemiotis, C.; Newkome, G. R. *J. Am. Chem. Soc*. **2012**, *134*, 7672.

Wang, J.-L., Li, X., Lu, X. C., Hsieh, I-F., Cao, Y., Moorefield, C. N., Wesdemiotis, C., Cheng, S. Z. D., Newkome, G. R., *J. Am. Chem. Soc*. **2011**, *133*, 11450-11453.

Ayme, J.-F.; Beves, J. E.; Leigh, D. A.; McBurney, R. T.; Rissanen, K.; Schultz, D. *Nat. Chem.* **2012**, *4*, 15.

Sun, B.; Wang, M.; Lou, Z.; Huang, M.; Xu, C.; Li, X.; Chen, L.-J.; Yu, Y.; Davis, G. L.; Xu, B.; Yang, H.-B.; Li, X. *J. Am. Chem. Soc.* **2015**, *137*, 1556.
